# Efficacy and safety of acupuncture for septic gastrointestinal dysfunction: a systematic review and meta-analysis of randomized controlled trials

**DOI:** 10.3389/fmed.2026.1680999

**Published:** 2026-02-26

**Authors:** Jian Xu, Jiahua Li, DingWei Deng, Junxuan Wu, BoJun Zheng, Jian Li

**Affiliations:** 1 The Second Clinical School of Medicine, Guangzhou University of Chinese Medicine, The Second Affiliated Hospital of Guangzhou University of Chinese Medicine, Guangzhou, Guangdong, China; 2 Guangdong Provincial Key Laboratory of Research on Emergency in Traditional Chinese Medicine, Guangzhou, Guangdong, China; 3 School of Pharmaceutical Sciences, Zhaoqing Medical College, Zhaoqing, Guangdong, China

**Keywords:** acupuncture, acute gastrointestinal injury, gastrointestinal dysfunction, meta-analysis, sepsis, systematic review

## Abstract

**Background:**

Septic gastrointestinal dysfunction (S-GID) lacks effective therapeutic approaches. Acupuncture has been widely used to treat S-GID; however, its efficacy and safety lack high-quality evidence-based support, particularly from randomized controlled trials (RCTs).

**Methods:**

A comprehensive search of PubMed, Embase, The Cochrane Library, and four other Chinese databases was conducted for all years up to September 2023 of acupuncture for S-GID. Additionally, research progress was reviewed in the Chinese Clinical Trials Registry and ClinicalTrials.gov. The analysis was conducted using RevMan5.3 and STAT13.1. Continuous data were evaluated by the mean difference (MD)/the standard mean difference (SMD) and 95% confidence intervals (CIs). Dichotomous data were used to calculate the relative risk (RR)/the odds ratio (OR) with 95% CI. The quality of the data was assessed using the Risk of Bias Tool 2 and the GRADEpro GDT tool.

**Results:**

Thirteen RCTs with 865 patients were included for the analysis. Compared with the group of the standard treatment, the combination of acupuncture and the standard treatment for S-GID effectively reduced the intra-abdominal pressure (IAP; SMD = −0.71; 95% CI: −1.01, −0.41, *p* < 0.001), the acute gastrointestinal injury grade (AGI; MD = −0.44; 95% CI: −0.65 to −0.23; *p* < 0.001), the Acute Physiology and Chronic Health Evaluation-II score (APACHE II; MD = −1.99; 95% CI: −3.04, −0.95, *p* < 0.001), and abdominal perimeter (AP; MD = −2.24; 95% CI: −3.49 to −1.00; *p* < 0.001), and increased the frequency of borborygmus per minute (FOB; MD = 0.85; 95% CI: 0.52–1.18; *p* < 0.001). No significant difference was found between these two groups in both mortality at day 28 (RR = −0.74; 95% CI: 0.49–1.11; *p* = 0.14) and the incidence of adverse events (OR = 1.01; 95% CI: 0.22–4.58; *p* = 0.99).

**Conclusion:**

This study indicated that, in S-GID patients, combining conventional treatment with acupuncture may reduce IAP, AP value, and AGI grade, increase FOB values, and lower the APACHE II score with good safety. However, the 28-day mortality data showed no significant difference, likely due to insufficient sample size. A multicenter, randomized, double-blind controlled study is required for further confirmation.

**Systematic review registration:**

https://www.crd.york.ac.uk/PROSPERO/view/CRD42022375480, identifier PROSPERO (CRD42022375480).

## 1 Introduction

Sepsis, a life-threatening organ dysfunction caused by a dysregulated host response to infection, along with septic shock, leads to high mortality globally (1, 2). Data from high-income countries showed 31.5 million sepsis cases and 5.3 million deaths among 19.4 million cases of severe sepsis annually during the period from 1979 to 2015 (3). Due to limited comparable data, the situation in low- and middle-income countries may be worse. However, hospital admissions and hospital mortality of sepsis and severe sepsis were still up to 17 and 26%, respectively (3).

Septic gastrointestinal dysfunction (S-GID) is a common complication of sepsis, playing a key role in the initiation and aggravation of sepsis. Early-stage sepsis may cause intestinal barrier dysfunction, gastrointestinal motility disorder, and translocation of intestinal flora, leading to multiple organ dysfunction syndrome (MODS). Once MODS is initiated, it will contribute to the induction and aggravation of gastrointestinal dysfunction, accelerating hospitalization and mortality rates (4, 5). Thus, the treatment of S-GID is of great significance for sepsis. Clinical treatments for S-GID include early enteral nutrition, gastric motility drugs, acid inhibitors, and antimicrobial agents to protect gastrointestinal mucosa, increase gastrointestinal motility, and improve intestinal flora (4, 6, 7). However, despite clinical application of therapies for S-GID, the case fatality of Sepsis-3 has been estimated to be over 30%, corresponding to 700,437 deaths in China (8). Thus, the urgent exploration of novel and supplemental treatments is required to improve clinical outcomes.

There is growing evidence that acupuncture, along with Western medicine, ameliorates S-GID. Several trials have shown a positive effect of acupuncture as an adjuvant therapy for sepsis patients (9, 10). Our recent RCT demonstrated that combination electroacupuncture with standard treatment (11) could improve clinical outcomes by regulating the immune system compared with standard treatment alone (11). Acupuncture also improves S-GID, according to many RCT results focusing on acupuncture or a combination with standard treatment. When acupuncture was used as an adjuvant therapy, S-GID patients were significantly alleviated from acute gastrointestinal injury (AGI) grade and digestive system functions (12). However, findings regarding its impact on mortality have been inconsistent. Meng et al. reported a lower mortality of S-AGI patients at day 28 when using acupuncture as an adjuvant therapy than standard treatment alone (12). However, the results reported in other RCTs are negative (13, 14). Thus, the definite therapeutic effect of acupuncture on AGI needs to be clarified. However, no systematic review of acupuncture on AGI has been conducted yet. In this systematic review and meta-analysis, we summarize the efficacy and safety of acupuncture for S-GID to inform clinical practice. The following questions have been addressed: (1) whether acupuncture as an adjunct treatment combined with conventional medicine is more effective than conventional medicine alone; (2) whether acupuncture as an adjunct treatment is safe when combined with conventional medicine.

## 2 Materials and methods

This study adhered to the Preferred Reporting Items for Systematic Reviews and Meta-Analyses (PRISMA) standards (Page et al.) (PRISMA checklist) (15), and the protocol was registered in PROSPERO (No. CRD42022375480).

### 2.1 Criteria for the inclusion of studies

#### 2.1.1 Studies

All RCTs investigating the efficacy and safety of acupuncture treatment for S-GID were included, irrespective of blinding or publication type, while pseudo- or quasi-RCTs were excluded. For instance, clinical trials in which participants were assigned to the experimental or control group based on factors such as admission order, hospital identification number, date of birth, and day of the week were not considered.

#### 2.1.2 Participants

The inclusion criteria for this study encompassed adults (≥18 years old) who had received a confirmed diagnosis of sepsis and gastrointestinal dysfunction.

The diagnosis of sepsis was established based on the definition and diagnostic criteria outlined in the 2016 International Guidelines for the Management of Sepsis, which referred to an imbalanced response to infection that leads to dysfunction in vital organ systems, as indicated by a Sequential Organ Failure Assessment (SOFA) score ≥2 (16). In essence, sepsis can be diagnosed when there is a confirmed or suspected infection accompanied by a SOFA score ≥2 (16).

The diagnosis of gastrointestinal dysfunction was established in accordance with the definition and diagnostic criteria outlined in the 2012 guideline from the European Society of Intensive Medicine (ESICM) Working Group (4). Aiming at the measurement of gastrointestinal function, the acute gastrointestinal injury (AGI) grade was used (4). The AGI grade has been widely used to assess gastrointestinal dysfunction in patients with acute critical illness. The specific criteria for AGI grading are detailed in Supplementary file S1. Fulfilling any grade of acute gastrointestinal injury (AGI) qualified for a diagnosis of gastrointestinal dysfunction.

#### 2.1.3 Interventions

Patients in the control group received standard treatment, including initial resuscitation, administration of antimicrobial agents, fluid therapy, early enteral nutrition, gastric motility drugs, acid inhibitors, and others. The definition of standard treatment was based on the guidelines provided by the “Surviving Sepsis Campaign: International Guidelines for Management of Sepsis and Septic Shock: 2016” and “Gastrointestinal function in intensive care patients: terminology, definitions, and management” (4, 17).

Conversely, the intervention group received acupuncture in addition to the standard treatment. The acupuncture treatment had no specific requirements regarding acupoint selection, number of acupoints used, duration, or frequency. If the intervention group received any additional interventions, the control group likewise received them. Trials that compared different forms of acupuncture or involved oral traditional Chinese medicine (TCM), such as Chinese herbal prescriptions or Chinese patent drugs, were excluded from this study.

Acupuncture is the insertion of needles, with or without manual or electrical stimulation, at specific acupuncture points, pain points, or trigger points. The following types of studies were not included in the trials: (1) Studies that only used micro-systems like ear acupuncture without body acupuncture; (2) studies that focused on drug injections at acupuncture points; (3) studies utilizing non-needle techniques such as instantaneous electrical stimulation, acupressure, laser acupuncture, or drug patch stimulation to activate acupuncture points; (4) studies involving oral administration of TCM, including Chinese herbal prescriptions or Chinese patent drugs.

#### 2.1.4 Outcome measures

##### 2.1.4.1 Primary outcomes

- Intra-abdominal pressure (IAP)
- Mortality on day 28
- Grade of acute gastrointestinal injury (AGI) (according to 2012 ESICM guidelines) (4)

##### 2.1.4.2 Secondary outcomes

- Acute Physiology and Chronic Health Evaluation-II score (APACHE II)
- Frequency of borborygmus (FOB)
- Abdominal perimeter (AP)
- Adverse events

### 2.2 Exclusion criteria

The exclusion criteria for articles were as follows: (1) redundant publications and (2) those lacking the essential outcome data needed for meta-analysis.

### 2.3 Search strategy

From the beginning until September 2023, seven databases were systematically searched, including the VIP information resource integration service platform (CQVIP), Wanfang Data Knowledge Service Platform, China National Knowledge Infrastructure (CNKI), Chinese Biomedical Literature Service System (Sinomed), Cochrane Central Register of Controlled Trials (CENTRAL), Embase, and PubMed. The search was conducted without language or publication status restrictions using a comprehensive strategy that combined subject terms and free words. Additionally, research progress was reviewed in the Chinese Clinical Trials Registry (CHiCTR) and ClinicalTrials.gov. Supplementary file S2 provides detailed information on the search strategy and results obtained from the bibliographic databases.

### 2.4 Data collection and analysis

#### 2.4.1 Selection of studies, data extraction, and management

To minimize redundancy, all records were imported into the reference management software (EndNote X9). Two independent reviewers assessed the eligibility of the studies based on predefined inclusion/exclusion criteria. Reviews and drug trials were excluded by assessing titles and abstracts for irrelevance. Full texts were examined before confirming inclusion, while papers with unclear titles or abstracts underwent additional evaluation by the reviewers. When authors published the same data in multiple studies, preference was given to the most recent or the one with the largest published sample size. A standardized form was used for data extraction to facilitate statistical analysis, which included (1) study identification number, (2) sample size, (3) initial patient characteristics (gender, age, severity of AGI, and APACHE II scores), (4) treatment specifics (acupoint selection, manual or electroacupuncture treatment, duration and frequency of administration), and (5) outcomes and adverse reactions. In case of any confusion or missing information, the authors of the original studies were consulted for clarification. Any discrepancies were resolved through discussion between the two reviewers or with another researcher.

#### 2.4.2 Assessment of the risk of bias

The Cochrane Collaboration Risk of Bias 2.0 (RoB 2), based on the Cochrane Handbook for Systematic Reviews, was independently employed by two reviewers to evaluate the methodological quality of all studies included in this review (18). In case of discordance, the final decision was reached through consultation with a third reviewer. The tool comprises the following categories: yes (Y), probably yes (PY), no (N), probably no (PN), and no information (NI). Potential bias may arise from various factors, including the randomization process, deviations from the intended interventions, missing outcome data, and measurement bias in the reporting of results.

#### 2.4.3 Data synthesis and analysis

The effect size was pooled using Review Manager Software (RevMan, v.5.3; The Cochrane Collaboration). For continuous data, a fixed-effect model was used to estimate the mean difference (MD) in the pooled data when outcomes were measured using standardized scales; otherwise, the standard mean difference (SMD) was computed. Additionally, 95% confidence intervals (CIs) were utilized. Dichotomous data are expressed as the relative risk (RR) with 95% CI. As the number of adverse events was usually low, the odds ratio (OR) was calculated instead of RR. An OR >1 indicated more events (for example, dropouts) in the acupuncture group (19). The statistical methods employed to assess heterogeneity included applying the χ^2^ test and the inconsistency index statistic (*I*^2^). In instances where there was significant variability (*I*^2^ > 50% or *p* < 0.05), a random-effects model was adopted; otherwise, a fixed-effects model was used. Subgroup, meta-regression, and sensitivity analyses were conducted to investigate potential factors contributing to the observed heterogeneity.

#### 2.4.4 Analysis of subgroup and meta-regression

These pre-established assumptions about subgroups were employed to analyze subgroup effects and meta-regression.

- APACHE-II score at baseline
- Method of acupuncture (manual or electroacupuncture)
- Intervention duration

#### 2.4.5 Sensitivity analysis

Given substantial heterogeneity among the included studies, a sensitivity analysis was conducted to identify potential sources of heterogeneity and evaluate the robustness and reliability of each step in the meta-analysis. Specifically, a sensitivity analysis was performed to assess whether there were any significant changes in both effect size estimates and heterogeneity when individual studies were excluded.

### 2.5 Assessment of publication biases

Publication bias was assessed using a funnel plot, provided that the number of experimental datasets exceeded 10. In cases where a slight asymmetry was observed in the funnel plot, Egger’s test was employed to confirm its existence. A *p* > 0.05 indicated the absence of any significant asymmetry.

### 2.6 Quality of evidence

The Grading of Recommendations Assessment, Development, and Evaluation (GRADE) methodology was employed to assess the certainty of evidence using GRADEpro GDT^1^ (20). RCT evidence was initially classified as high quality, but it may have been downgraded due to potential bias, inconsistency, indirectness, imprecision, or publication bias. The certainty of the evidence was categorized into four levels: very low, low, moderate, and high.

## 3 Results

### 3.1 Study descriptions

**Figure 1:**
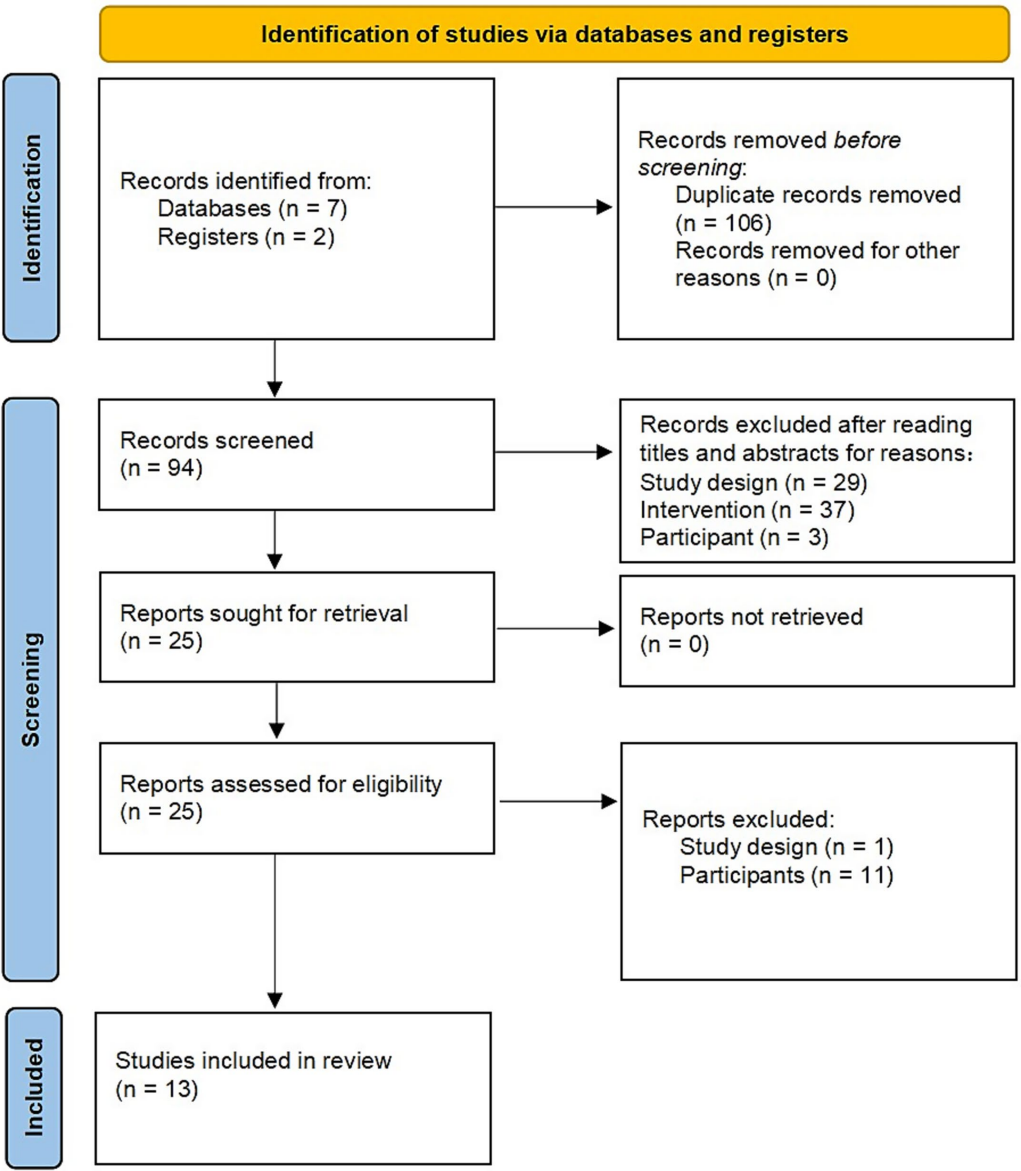
PRISMA flow diagram of the literature screening and selection process.

A total of 200 research articles related to the topic were retrieved, but after excluding 106 duplicate studies, 94 potentially relevant papers were included. After reviewing their titles and abstracts, 69 articles that did not meet the inclusion criteria were further excluded. The excluded studies are listed in Supplementary file S3 for more details. Subsequently, 25 potentially relevant studies were identified after a thorough review of their full texts. However, one of these studies was a review article, and the other described technological advancements. Because they did not meet the criteria for the RCT literature type, they were excluded from this systematic review and meta-analysis. Furthermore, 11 articles were excluded due to non-adherence to the Sepsis-3 criteria and the 2012 AGI grading standards for patient inclusion (Supplementary file S4). Finally, 13 RCTs were included in this study (12–14, 21–30). The selection process is illustrated in Figure 1.

### 3.2 Studies included in the analysis

**Figure 2:**
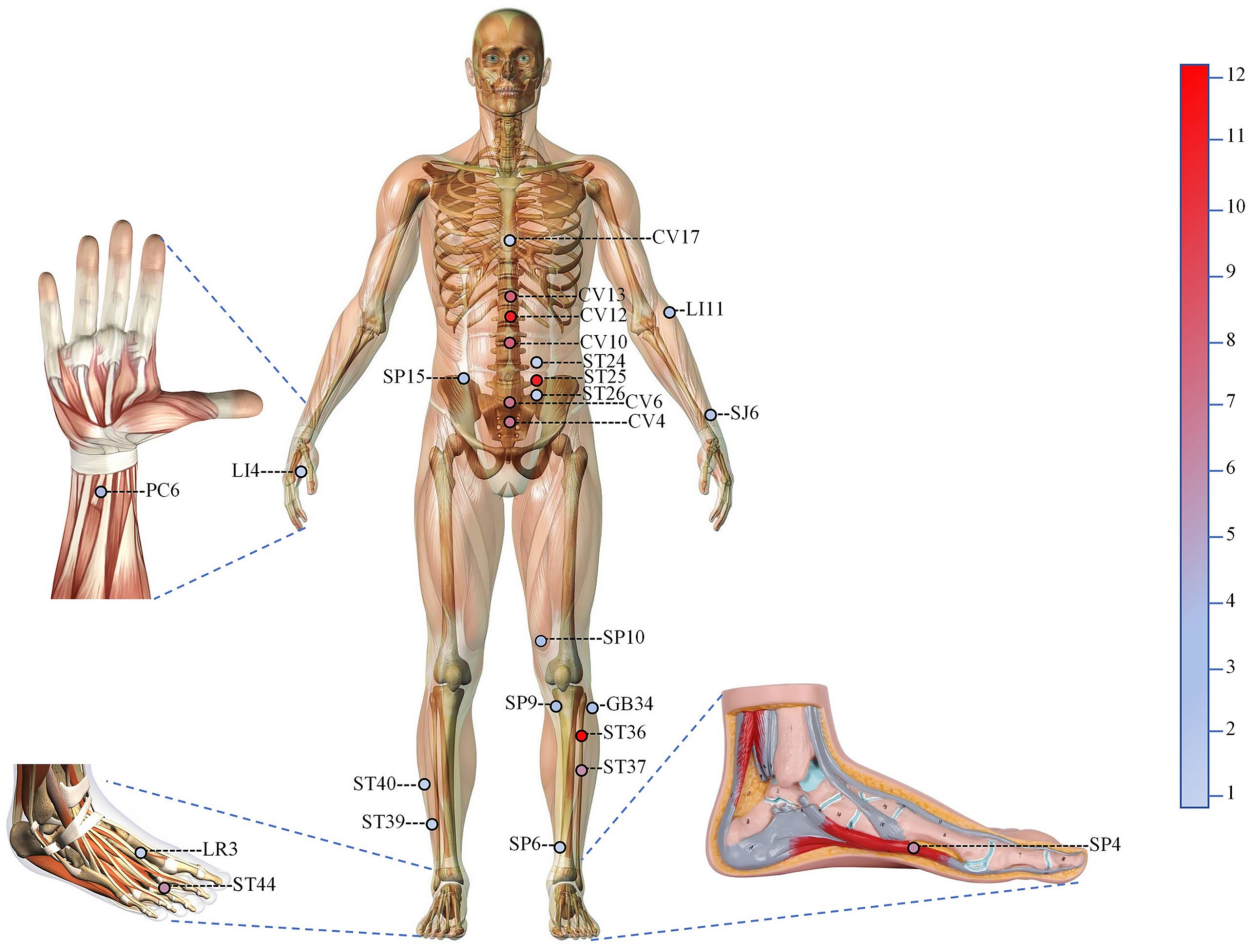
The locations of all relevant acupuncture points of included studies.

The baseline characteristics and details of acupuncture administration for all eligible studies are presented in Table 1. Among the 13 included studies, a total of 25 acupoints were involved, with frequencies ranging from 1 to 12 times. The top six most frequently used acupoints were Zusanli (ST36) (12 times), Tianshu (ST25) (12 times), Zhongwan (CV12) (12 times), Xiawan (CV10) (8 times), Shangwan (CV13) (7 times), and Qihai (CV6) (7 times). The frequency for each acupoint is presented in Table 2; Figure 2. The specific acupoints involved in each study are detailed in Supplementary file S5. The meta-analysis included 13 RCTs (12–14, 21–30), comprising 865 participants, conducted in Chinese hospitals between 2017 and 2022. Sample sizes ranged from 40 to 130, while the average age of the population varied from 51 to 80 years. Baseline APACHE II scores ranged from 14 to 26. Manual acupuncture (MA) was employed in 10 trials (13, 14, 21–24, 26–29), whereas electroacupuncture (EA) was utilized in three trials (12, 25, 30). Treatment duration spanned from 5 to 28 days. Five studies reported that their funding sources were national/provincial/hospital-based (12, 13, 22, 25, 26).

**Table 1:** Relevant characteristics of the included trials.

Study ID	Sample Size (I/C)	Age, Year (I/C)	No. of males (I/C)	APACHE II	AGI classification (I/C)	Co-intervention	Treatment	Comparison	Duration of the acupuncture treatment	Follow-up	Outcomes	Funding
Wei, 2017	41(20/21)	71.9 ± 15.2/68.3 ± 14.8	15/13	23.9 ± 4.1/25.5 ± 5.3	II:4; III:16/ II:6; III:15	ST	MA	–	28 d	28 d	①②③④⑤⑥⑦	NR
Meng, 2018	82 (41/41)	63.51 ± 14.54/61.32 ± 13.96	19/22	18.93 ± 3.98/18.66 ± 4.18	All degrees	ST	EA	–	5 d	28 d	①②⑦	By ZPATCM
Yang, 2018	60 (30/30)	66.5 ± 12.3/70.1 ± 9.9	18/16	23.37 ± 5.67/24.03 ± 5.25	II–III	ST	MA	–	7 d	28 d	①②④⑤⑥⑦	By SPNSF; SPKRDP
Wang XP, 2019	40 (20/20)	62.10 ± 15.59/62.55 ± 15.73	8/14	23.90 ± 4.87/24.40 ± 5.22	All degrees	ST	MA	-	7 d	7 d	①④⑤⑥⑦	NR
Liu et al., 2020	98 (49/49)	56 ± 6/56 ± 6	28/23	NR	All degrees	ST	MA	Domperidone	7 d	NR	①⑤⑦	By BMHRCP
Wang LJ, 2019	58 (29/29)	63.68 ± 13.58/64.75 ± 15.84	18/14	14.79 ± 3.69/13.25 ± 3.98	I:19; II:8/I:22; II:6	ST	MA	–	5 d	28 d	①②③④⑤⑦	NR
Li et al., 2021	57 (28/29)	73.75 ± 6.12/72.06 ± 7.58	14/16	21.30 ± 1.93/21.61 ± 2.02	AGI ≥ II	ST	MA	Gastrointestinal motility drugs	7 d	7 d	①④⑥	NR
Lian et al., 2021	70 (35/35)	80.71 ± 8.02/78.51 ± 9.61	22/19	20.94 ± 3.89/19.69 ± 3.55	II:17; III:18/ II:15; III:20	ST	EA	-	7 d	7 d	①③④	By STCMDTMIPCP and SUTCMAYITCWHFYP
Ban et al., 2022	73 (37/36)	70.22 ± 9.14/70.32 ± 8.58	19/18	21.16 ± 2.21/21.08 ± 2.38	II–III	ST	MA	Gastrointestinal motility drugs	7 d	7 d	①④⑥	By LTIEP
Chang, 2022	40 (20/20)	77.55 ± 7.90/73.00 ± 10.57	13/12	22.45 ± 7.45/21.00 ± 6.60	All degrees	ST	MA	-	7 d	14 d	③⑤⑦	NR
Cheng et al., 2022	63 (31/32)	77.71 ± 13.78/73.00 ± 14.32	11/13	17.71 ± 5.99/16.35 ± 7.04	I–III	ST	MA	-	7 d	7 d	③④⑦	NR
Peng et al., 2022	130 (65/65)	51.96 ± 5.42/51.42 ± 4.63	33/35	21.96 ± 4.24/21.91 ± 5.74	All degrees	ST	MA + Early enteral nutrition	Early enteral nutrition	14 d	14 d	①③④⑤⑥	NR
Sun et al., 2022	53 (27/26)	72.4 ± 14.63/73.81 ± 15.58	20/20	24.2 ± 2.68/23.12 ± 2.27	All degrees	ST	EA + Early enteral nutrition	Early enteral nutrition	14 d	28 d	①②③④⑦	NR

I, intervention group; C, control group; AGI: acute gastrointestinal injury; RCT, randomized controlled trial; ST, standard treatment, MA, manual acupuncture; EA, electroacupuncture; NR, not reported; outcomes: ①, intra-abdominal pressure (IAP); ②, mortality at day 28; ③, grade of acute gastrointestinal injury; ④, Acute Physiology and Chronic Health Evaluation-II (APACHE-II); ⑤, frequency of borborygmus (FOB); ⑥, abdominal perimeter (AP); ⑦, adverse events; NR: not reported; ZPATCM, the Zhejiang Provincial Administration of Traditional Chinese Medicine, China; SPNSF, Shandong Provincial Natural Science Foundation; SPKRDP, Shandong Provincial Key Research and Development Program; BMHRCP, Beijing Municipal Hospital Research Cultivation Program; STCMDTMIPCP, Shanghai Traditional Chinese Medicine Diagnosis and Treatment Model Innovation Pilot Construction Project; SUTCMAYITCWHFYP, Shanghai University of Traditional Chinese Medicine Affiliated Yueyang Integrated Traditional Chinese and Western Medicine Hospital Fund Youth Project; LTIEP, the Lanzhou Talent Innovation and Entrepreneurship Project.

**Table 2:** Summary of Acupoint Utilization in Included Studies.

No.	Acupoint name	Frequency	Reference numbers	Location
1	Zusanli (ST36)	12	(12–14, 21, 22, 24–30)	On the anterior aspect of the lower leg, 3 cun below ST35 (Dubi), one finger-breadth lateral to the anterior crest of the tibia.
2	Tianshu (ST25)	12	(13, 14, 21–30)	On the abdomen, 2 cun lateral to the center of the umbilicus.
3	Zhongwan (CV12)	12	(13, 14, 21–30)	On the upper abdomen, 4 cun above the center of the umbilicus, on the anterior median line.
4	Xiawan (CV10)	8	(13, 14, 21, 23–26, 29)	On the upper abdomen, 2 cun above the center of the umbilicus, on the anterior median line.
5	Shangwan (CV13)	7	(13, 14, 21, 25, 26, 29, 30)	On the upper abdomen, 5 cun above the center of the umbilicus, on the anterior median line.
6	Qihai (CV6)	7	(13, 23–27, 30)	On the lower abdomen, 1.5 cun below the center of the umbilicus, on the anterior median line.
7	Guanyuan (CV4)	7	(22–27, 30)	On the lower abdomen, 3 cun below the center of the umbilicus, on the anterior median line.
8	Shangjuxu (ST37)	6	(12, 22, 25, 27, 28, 30)	On the anterior aspect of the lower leg, 6 cun below ST35 (Dubi), 3 cun below ST36 (Zusanli).
9	Neiguan (PC6)	4	(13, 22, 27, 28)	On the anterior aspect of the forearm, 2 cun proximal to the palmar wrist crease, between the tendons of palmaris longus and flexor carpi radialis.
10	Yanglingquan (GB34)	3	(14, 21, 29)	On the lateral aspect of the lower leg, in the depression anterior and inferior to the head of the fibula.
11	Yinlingquan (SP9)	3	(27, 28, 30)	On the medial aspect of the lower leg, in the depression between the lower border of the medial condyle of the tibia and the medial border of the tibia.
12	Xiajuxu (ST39)	3	(22, 25, 28)	On the anterior aspect of the lower leg, 9 cun below ST35 (Dubi), 3 cun below ST37 (Shangjuxu).
13	Daheng (SP15)	2	(23, 25)	On the abdomen, 4 cun lateral to the center of the umbilicus.
14	Gongsun (SP4)	2	(24, 26)	On the medial side of the foot, in the depression distal and inferior to the base of the first metatarsal bone, at the junction of the red and white skin.
15	Neiting (ST44)	2	(27, 28)	At the elbow, midpoint of the line connecting LU5 (Chize) with the lateral epicondyle of the humerus.
16	Quchi (LI11)	1	(27)	On the dorsum of the foot, between the 2nd and 3rd toes, at the margin of the web, at the junction of the red and white skin.
17	Danzhong (CV17)	1	(27)	On the chest, level with the 4th intercostal space, on the anterior median line.
18	Fenglong (ST40)	1	(27)	On the lateral aspect of the lower leg, 8 cun superior to the tip of the external malleolus, one finger-breadth lateral to ST38 (Tiaokou)
19	Hegu (LI4)	1	(30)	On the dorsum of the hand, between the 1st and 2nd metacarpal bones, approximately at the midpoint of the 2nd metacarpal bone on the radial side.
20	Sanyinjiao (SP6)	1	(30)	On the medial aspect of the lower leg, 3 cun superior to the tip of the medial malleolus, posterior to the medial border of the tibia.
21	Taichong (LR3)	1	(30)	On the dorsum of the foot, in the depression proximal to the 1st and 2nd metatarsal bones, at the junction of the bases.
22	Huaroumen (ST24)	1	(23)	On the abdomen, 1 cun superior to the center of the umbilicus, 2 cun lateral to the anterior median line.
23	Wailing (ST26)	1	(23)	On the abdomen, 1 cun inferior to the center of the umbilicus, 2 cun lateral to the anterior median line.
24	Xuehai (SP10)	1	(27)	On the anterior aspect of the thigh, 2 cun superior to the medial end of the base of the patella, on the bulge of the vastus medialis muscle.
25	Zhigou (TE6)	1	(27)	On the posterior aspect of the forearm, 3 cun proximal to the dorsal wrist crease, midpoint of the interosseous space between the ulna and radius.

### 3.3 Risk of bias in the included studies

**Figure 3:**
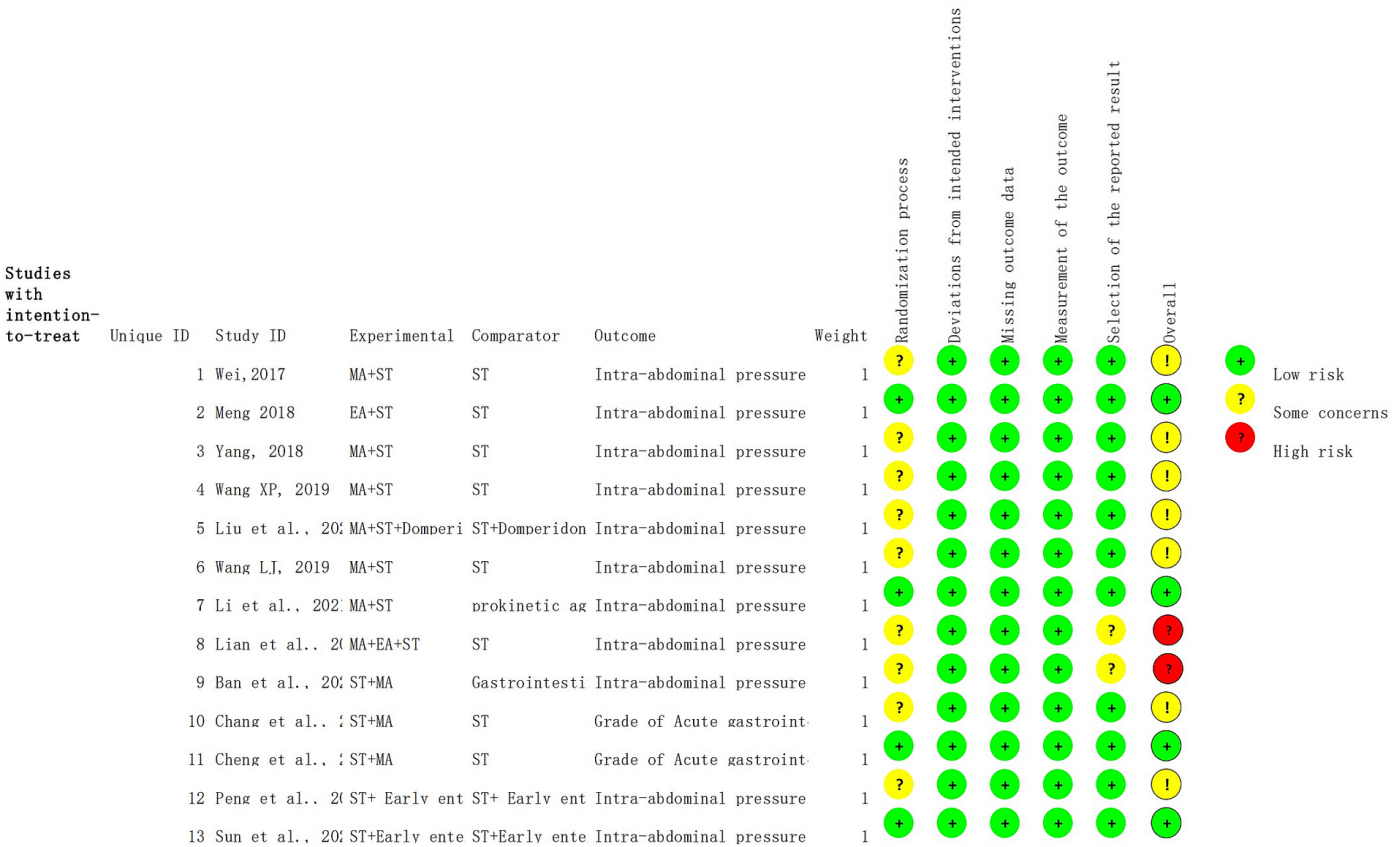
Risk of bias summary.

**Figure 4:**
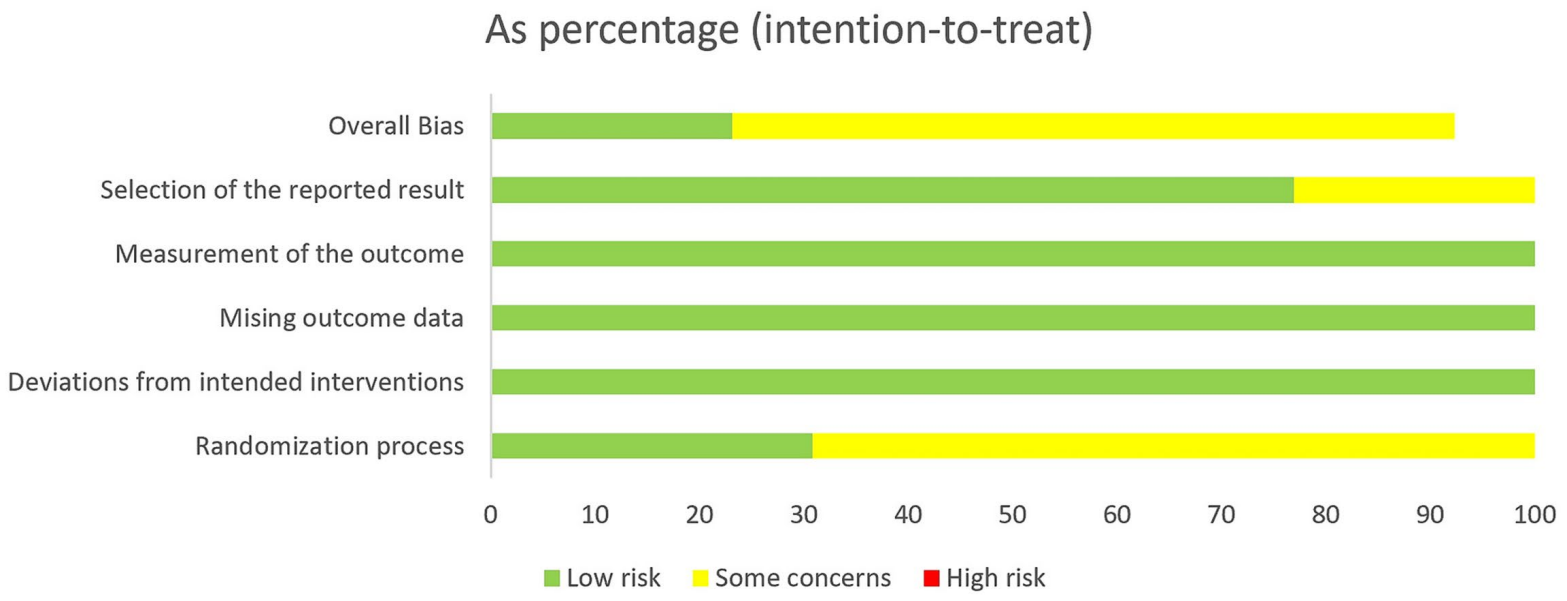
Risk of bias graph.

The risk of bias for the studies is depicted in Figures 3, 4. The Cochrane ROB 2.0 tool was used to assess risk of bias. All included studies were RCTs; 11 RCTs correctly implemented randomization methods, while only two RCTs reported a statement of randomization. Additionally, four articles described appropriate methods for concealing random sequences, while the remaining nine did not explicitly mention this approach. The baseline data showed consistency across all literature sources. Thus, these four articles were categorized as “Low risk” and classified the remaining nine as having “Some concerns.” Two of these articles mentioned adverse events in their methodology section but failed to report relevant data in their results section, indicating a potential risk of selective reporting; hence, they were also assigned to the “Some concerns” category. Other factors, such as deviations from intended interventions, missing outcome data, and outcome measurement, were judged as “Low risk.”

### 3.4 Effects of the interventions

#### 3.4.1 Primary outcomes

##### 3.4.1.1 Intra-abdominal pressure

**Figure 5:**
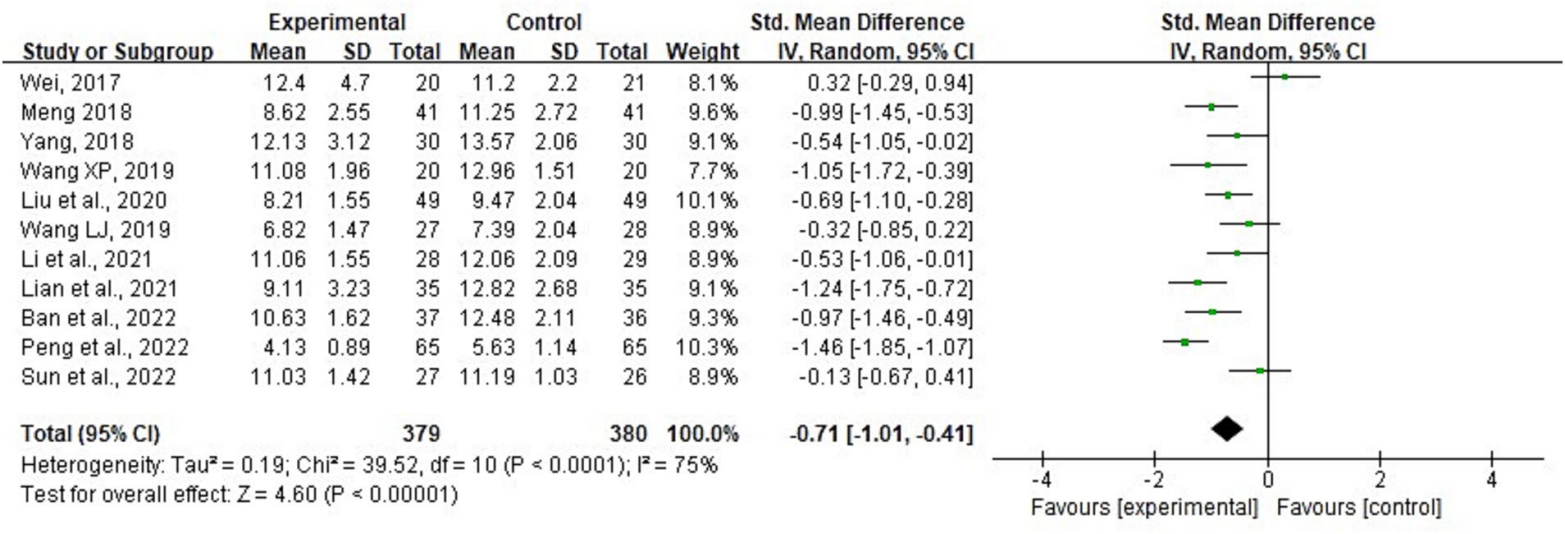
Forest plot and meta-analysis of intra-abdominal pressure.

**Figure 6:**
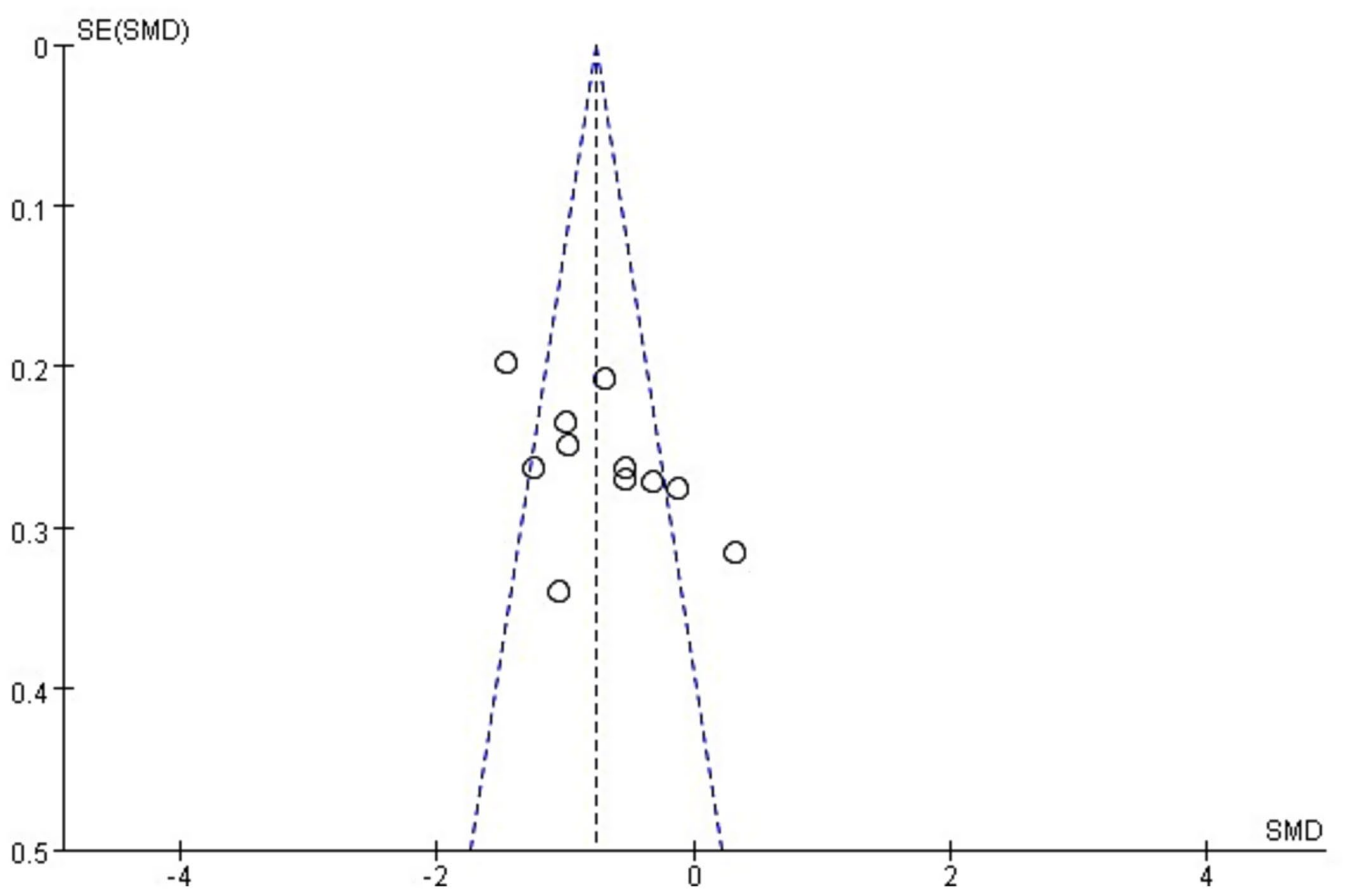
Funnel plot of intra-abdominal pressure.

A total of 11 studies involving 759 patients reported IAP (12–14, 21–26, 29, 30). The meta-analysis revealed significant heterogeneity in the index level of IAP (*p* < 0.001, *I*^2^ = 75%). Thus, a random-effects model was used for the meta-analysis. The results showed that with conventional medical treatment combined with acupuncture, the IAP value in AGI patients was statistically decreased (SMD = −0.71; 95% CI: −1.01, −0.41; *p* < 0.001) (Figure 5). Sensitivity analyses were performed by excluding each study separately. However, the significance of heterogeneity did not change, and the statistical significance of the results remained unchanged, indicating stability. Meta-regression analysis showed that heterogeneity was not associated with baseline APACHE II score (≥22 vs. <22), acupuncture method (manual acupuncture vs. electroacupuncture), or treatment duration (5, 7, 14, or 28 days), with corresponding values of 0.739, 0.835, and 0.233. Subgroup analyses based on baseline APACHE II score (≥22 vs. <22) and acupuncture methods (manual acupuncture or electroacupuncture) did not reveal significant differences for these factors (*p* = 0.38 and 0.75, respectively). However, subgroup analysis by treatment durations (5, 7, 14, or 28 days) showed a significant difference (*p* = 0.03). Furthermore, the heterogeneity observed in the subgroups with treatment durations of 5 and 7 days decreased, suggesting that treatment duration may contribute to the heterogeneity (Supplementary files S6.1–6.3). Figure 6 illustrates that the distribution of the included studies was generally symmetrical without obvious publication bias. Additionally, Egger’s test yielded non-significant results (*p* = 0.92).

##### 3.4.1.2 Mortality at day 28

**Figure 7:**
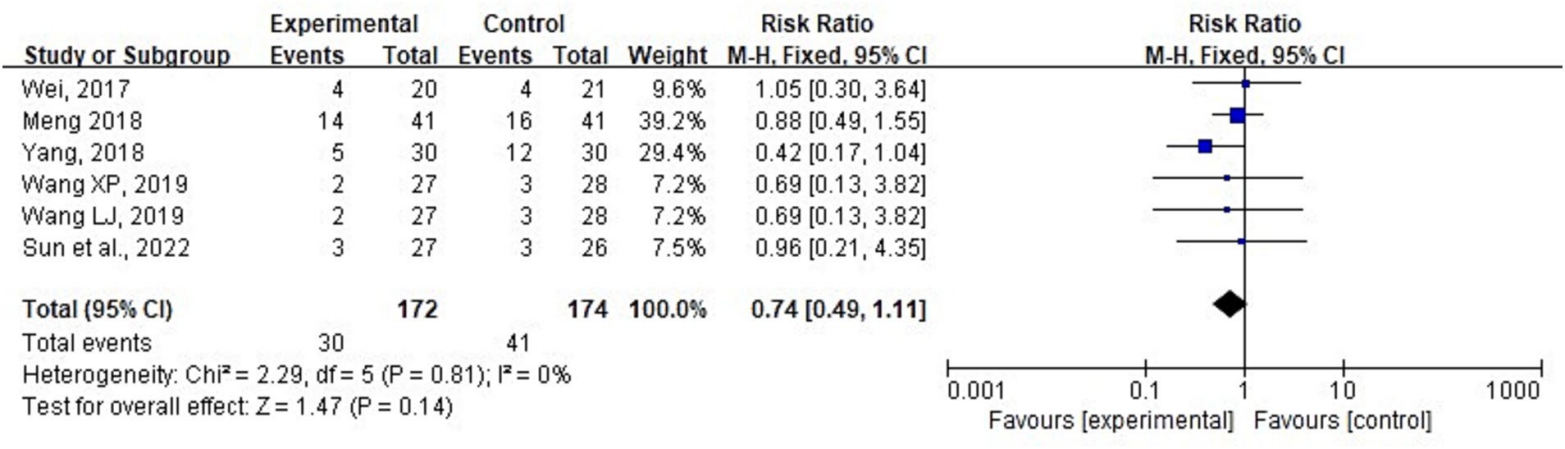
Forest plot and meta-analysis of mortality at day 28.

Six studies involving 346 patients were included in the analysis of mortality at day 28. Due to the minimal heterogeneity observed (*p* = 0.81, *I*^2^ = 0%), a fixed-effects model was employed for meta-analysis. Figure 7 illustrates that combining acupuncture with conventional medicine did not yield a statistically significant difference in mortality at day 28 compared with conventional medicine alone (RR = −0.74; 95% CI: 0.49–1.11; *p* = 0.14). Sensitivity analyses were performed by excluding each study separately. However, the significance of heterogeneity and the results did not change statistically, indicating stable results. Subgroup analyses based on different APACHE II baseline scores (≥22 or <22), treatment durations (5, 7, 14, or 28 days), and acupuncture methods also revealed that no significant differences were associated with these factors (*p* = 0.42, 0.63, and 0.36, respectively) (Supplementary files S6.4–6.6).

##### 3.4.1.3 AGI grade

**Figure 8:**
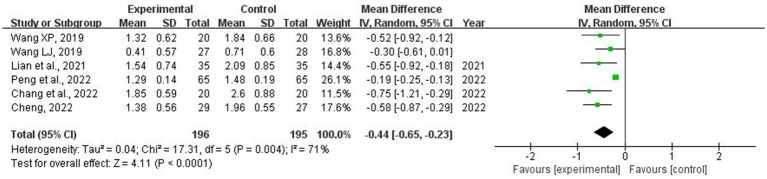
Forest plot and meta-analysis of acute gastrointestinal injury grade.

A total of six RCTs (14, 23, 25, 27–29) involving 391 patients were used for collecting data on AGI grade. The meta-analysis revealed significant heterogeneity (*p* = 0.004, *I*^2^ = 71%). Sensitivity analyses were conducted by systematically excluding individual studies. Upon removing the study conducted by “Peng” (29), the heterogeneity between studies was statistically reduced (*I*^2^ = 0%). As indicated in Table 1, the patients included in this study were the youngest and had received the longest duration of acupuncture treatment, which may have contributed to the heterogeneity. Consequently, a random-effects model was employed for the meta-analysis. The results demonstrated that combining acupuncture with conventional medical treatment led to a decrease in AGI grade levels compared with conventional medicine alone (MD = −0.44; 95% CI: −0.65 to −0.23; *p* < 0.001) (Figure 8). Meta-regression analysis revealed no significant association between heterogeneity and baseline APACHE II score (≥22 or <22), acupuncture method (manual acupuncture vs. electroacupuncture), or treatment duration (5, 7, 14, or 28 days), with corresponding *p*-values of 0.948, 0.660, and 0.280. Subgroup analysis based on different acupuncture methods (manual acupuncture vs. electroacupuncture) did not reveal any statistically significant differences (*p* = 0.56). However, subgroup analyses stratified by APACHE II scores (≥22 vs. <22) and treatment durations (5, 7, 14, or 28 days) showed significant differences (*p* = 0.03 and *p* < 0.001). These two subgroups exhibited decreased heterogeneity, suggesting that baseline APACHE II scores and treatment duration may be potential sources of heterogeneity (Supplementary files S6.7–6.9).

#### 3.4.2 Secondary outcomes

##### 3.4.2.1 Apache-ii

**Figure 9:**
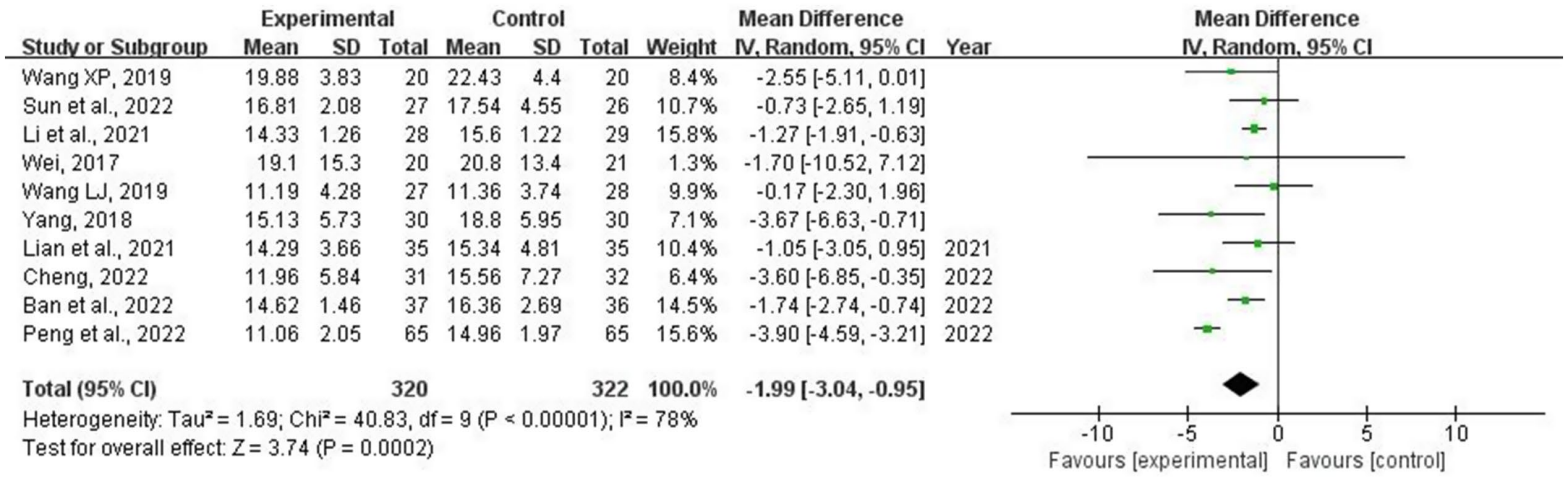
Forest plot and meta-analysis of the Acute Physiology and Chronic Health Evaluation (APACHE)-II.

**Figure 10:**
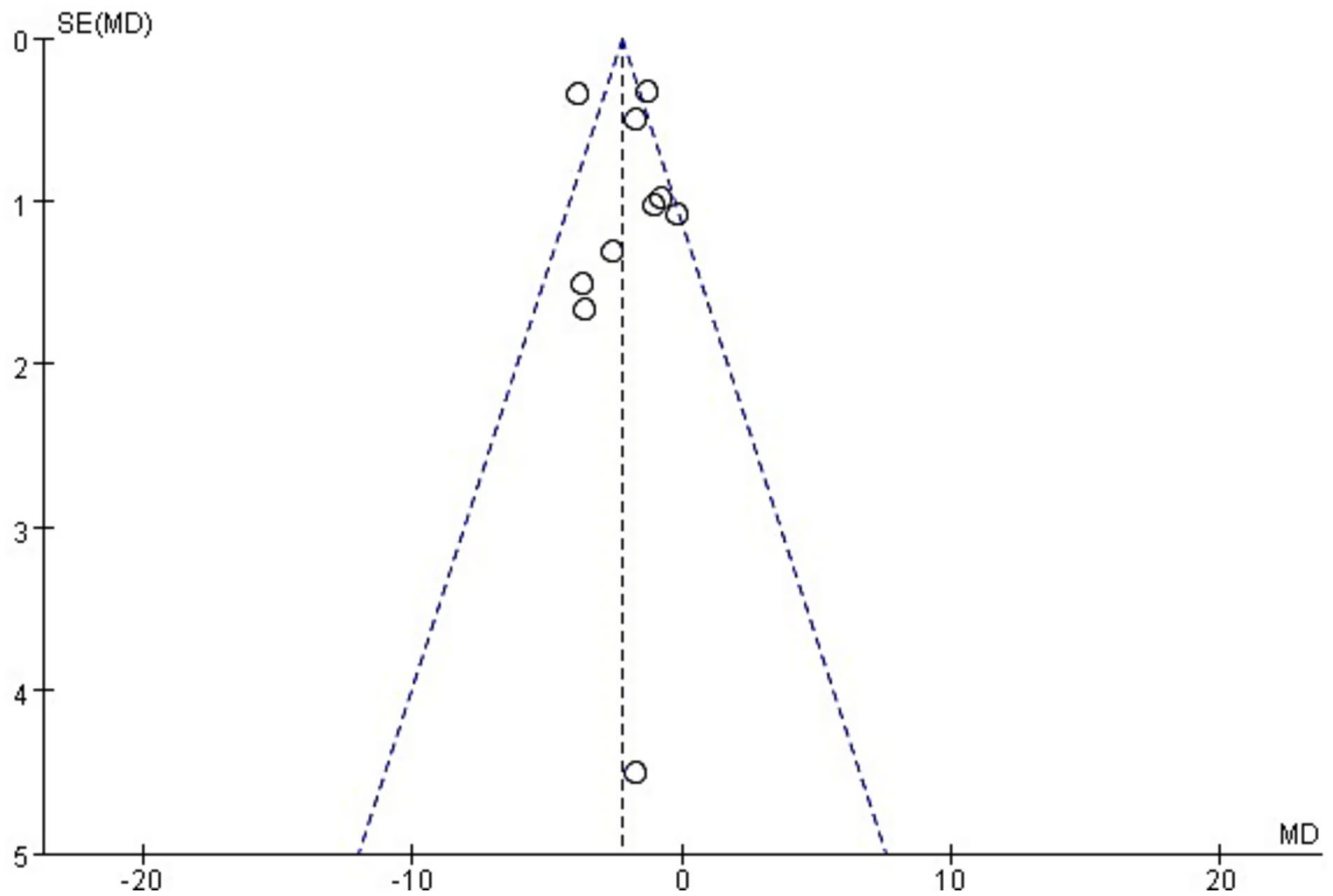
Funnel plot of the Acute Physiology and Chronic Health Evaluation (APACHE)-II.

The analysis included 10 studies (14, 21–26, 28–30) involving 642 patients. The meta-analysis revealed a significant heterogeneity in the APACHE II index (*p* < 0.001; *I*^2^ = 78%). Sensitivity analysis was performed by systematically excluding individual studies. After excluding the study conducted by “Peng” (29), the heterogeneity between studies was substantially reduced (*I*^2^ = 0%). As shown in Table 1, this study included the youngest patient group, which may have contributed to the observed heterogeneity. Therefore, a random-effects model was utilized for the meta-analysis. These findings indicated that combining acupuncture with conventional medicine treatment resulted in a significant decrease in APACHE II grade levels compared to using conventional medicine alone (MD = −1.99; 95% CI: −3.04, −0.95; *p* < 0.001) (Figure 9). Meta-regression analysis indicated that the observed heterogeneity was not attributable to the acupuncture method (manual acupuncture vs. electroacupuncture) or the treatment duration (5, 7, 14, or 28 days), with corresponding *p* values of 0.330 and 0.894. Subgroup analyses stratified by acupuncture techniques (manual acupuncture vs. electroacupuncture) and treatment durations (5, 7, 14, or 28 days) did not show significant differences with respect to these factors (*p* = 0.59 and 0.09, respectively). However, a reduction in heterogeneity was observed within the subgroups with a treatment duration of 7 days, suggesting that duration may contribute to heterogeneity (Supplementary files S6.10–6.11). Figure 10 depicts a symmetrical distribution of the included studies without indicating publication bias. Furthermore, Egger’s test yielded non-significant findings (*p* = 0.72).

##### 3.4.2.2 Frequency of borborygmus

**Figure 11:**
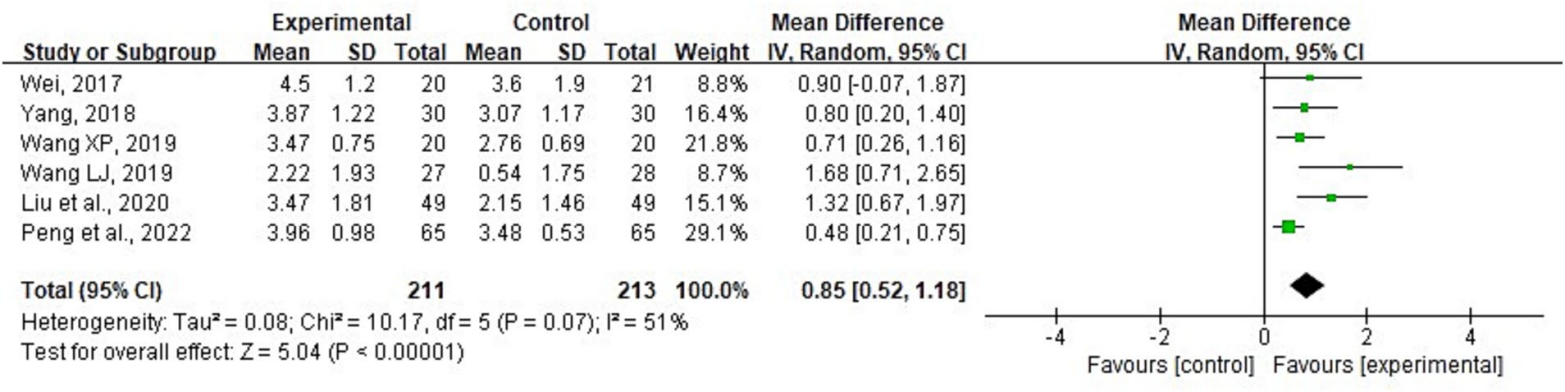
Forest plot and meta-analysis of the frequency of borborygmus per minute.

A total of six RCTs (13, 14, 21–23, 29) involving 424 patients provided FOB data. The meta-analysis revealed significant heterogeneity (*p* = 0.07; *I*^2^ = 51%). Sensitivity analyses were conducted by systematically excluding individual studies. Upon removing the studies conducted by “Wang and Liu, and Peng” (13, 23, 29), the heterogeneity between studies was statistically reduced (*I*^2^ = 35, 37, and 16%, respectively). However, the statistical significance of the results remained unchanged, indicating stability. Consequently, a random-effects model was employed for the meta-analysis. The results demonstrated that combining acupuncture with conventional medicine treatment led to an increase in FOB compared with conventional medicine alone (MD = 0.85; 95% CI: 0.52 to 1.18; *p* < 0.001) (Figure 11). Meta-regression showed no significant association of heterogeneity with either baseline APACHE II score (≥22 vs. <22; *p* = 0.976) or treatment duration (5, 7, 14, or 28 days; *p* = 0.418). Subgroup analyses based on baseline APACHE II scores (≥22 vs. <22) did not reveal any statistically significant differences (*p* = 0.09). However, subgroup analysis by treatment durations (5, 7, 14, or 28 days) revealed significant variations among subgroups (*p* = 0.05), suggesting that treatment duration may be a source of heterogeneity (Supplementary files S6.12–6.13). The MA was observed in all six studies; therefore, the inclusion of different acupuncture methods was omitted from the meta-regression and subgroup analyses.

##### 3.4.2.3 Abdominal perimeter

**Figure 12:**
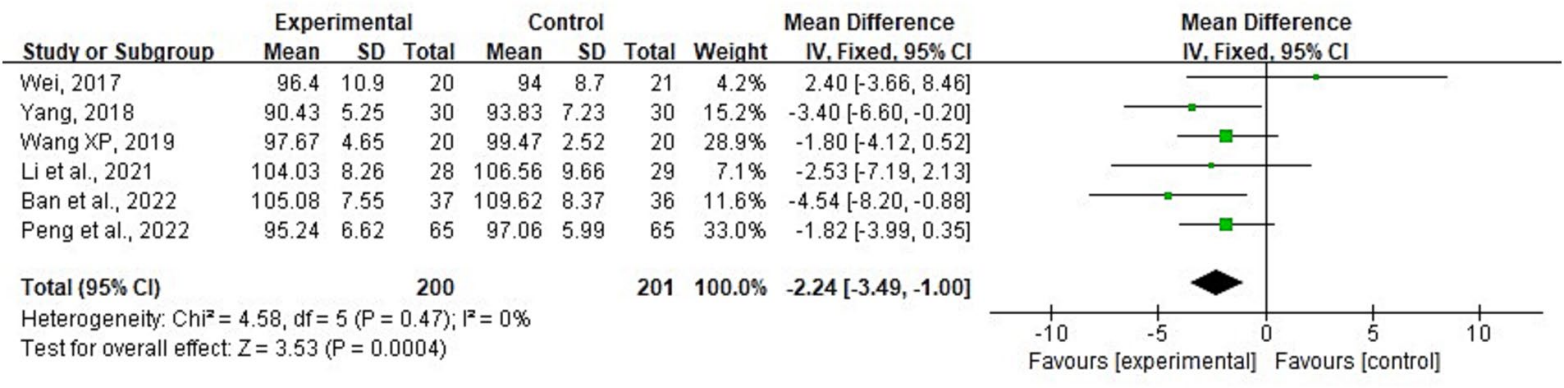
Forest plot and meta-analysis of the abdominal perimeter.

Six RCTs (14, 21, 22, 24, 26, 29) involving 401 patients were included in the analysis, providing data on changes in AP. The observed level of heterogeneity was minimal (*p* = 0.47; *I*^2^ = 0%), and a fixed-effects model was used for meta-analysis. Sensitivity analyses were performed by systematically excluding individual studies; however, the overall significance of the results remained stable. The findings indicated that combining acupuncture with conventional medicine resulted in a significant reduction in AP compared with conventional medicine alone (MD = −2.24; 95% CI: −3.49 to −1.00; *p* < 0.001) (Figure 12).

##### 3.4.2.4 Adverse events

**Figure 13:**
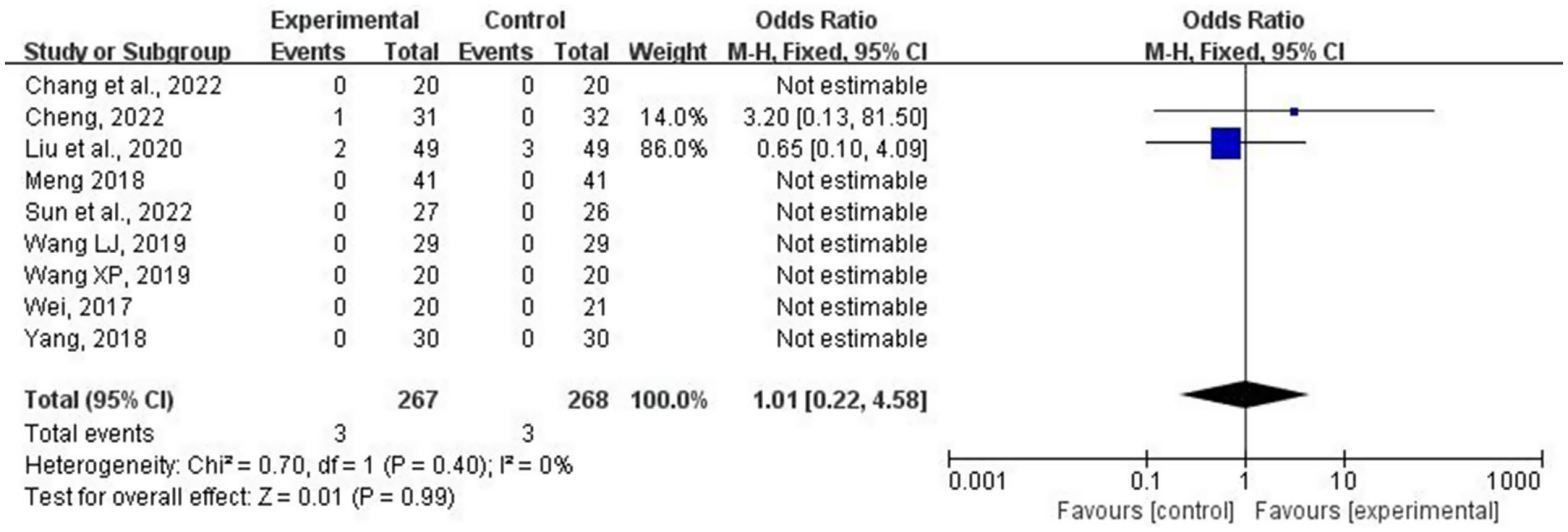
Forest plot and meta-analysis of the incidence of adverse events.

Four studies (24–26, 29) reported no adverse events, whereas nine trials (12–14, 21–23, 27, 28, 30) reported adverse events involving 535 patients. Specifically, seven studies reported no acupuncture-related accidents or adverse events. Liu et al. (13) reported three cases of adverse events in the control group: one case of drowsiness and two cases of diarrhea. In the treatment group, two cases of adverse events were reported involving drowsiness; however, these were tolerable and resolved without special intervention and did not require changes to treatment administration. Additionally, Cheng (28) documented a patient experiencing self-perceived abdominal distension that was initially intolerable but was alleviated after adjusting acupuncture depth with no subsequent impact on further treatments. As shown in Figure 13, the level of heterogeneity observed was minimal (*p* = 0.40, *I*^2^ = 0%), and a fixed-effects model was used for meta-analysis. The combination of acupuncture and conventional medicine did not significantly affect the incidence of adverse events compared to conventional medicine alone (OR = 1.01; 95% CI: 0.22–4.58; *p* = 0.99).

### 3.5 Assessment of the quality of evidence

The evidentiary quality of the outcomes varied from “moderate” to “very low.” The reason for downgrading was the unclear risk of bias and inconsistent results of the chosen studies, which can be attributed to substantial heterogeneity (Supplementary file S7).

## 4 Discussion

### 4.1 Summary of evidence

This systematic review aimed to compare the effectiveness and safety of combining acupuncture with conventional medicine versus using conventional medicine alone for S-AGI. It included 13 RCTs (12–14, 21–30) involving 865 participants for meta-analysis. Our study indicated that the combination of acupuncture and conventional medicine offered superior benefits in reducing IAP, AGI grade, APACHE II score, and AP. Moreover, it showed improvements in FOB compared with conventional medicine alone. However, a negative outcome was observed for 28-day mortality. Furthermore, meta-regression and subgroup analyses were performed to examine the effects of baseline APACHE II score, treatment duration, and different acupuncture methods (manual vs. electrical) for the treatment of S-AGI.

The severity of AGI, as defined by the AGI grading system (grades I-IV), is consistently and strongly associated with increased mortality across diverse populations of critically ill patients (31). The concept and grading system of the AGI grade were formally proposed and defined by the Working Group on Abdominal Problems of the European Society of Intensive Care Medicine (ESICM) in 2012 (4). Yszko et al. confirmed in a cohort study that a higher AGI score was associated with an increased 28-day mortality (32). Zhang et al. found that AGI grade independently predicted the odds of 28-day mortality in AGI patients in a prospective, single-center observational study that included 282 patients (31). Meta-analysis showed that acupuncture combined with conventional therapy was significantly better than conventional therapy alone in reducing the severity of AGI grade in S-GID patients. The sensitivity analysis suggested a robust result of lowering the AGI grade. However, the heterogeneity among studies was significant, which reduced the credibility of the results. Furthermore, subgroup analysis indicated that baseline APACHE II scores and treatment duration may be potential sources of heterogeneity.

We also observed that following the treatment of S-GID with acupuncture combined with conventional therapy, IAP and AP decreased significantly, whereas FOB increased significantly. IAP serves as a crucial objective indicator for the diagnosis and grading of AGI (4). As per the AGI grading criteria put forward by the European Society of Intensive Care Medicine, the level of IAP is directly used to define the severity of AGI (4). Clinical studies have demonstrated that AGI grading, of which IAP is an important component, is positively correlated with 28-day rates. Moreover, in multivariate analysis, IAP is an independent predictor of death (33). FOB and AP are simple, safe, and convenient indicators widely used to assess the gastrointestinal function of patients and evaluate the severity of AGI. The combination of IAP, gastrointestinal symptoms (such as FOB and AP), and feeding intolerance constitutes a feasible approach for assessing the severity of gastrointestinal dysfunction in critically ill patients (4). Our results indicated that acupuncture combined with conventional therapy was superior to conventional therapy alone in reducing IAP and AP and improving FOB in patients with S-GID. The sensitivity analysis indicated a robust reduction in IAP and AP and an improvement in FOB. However, we observed significant heterogeneity between the two outcome indicators (IAP and FOB), which reduced the credibility of the results. Conversely, no significant heterogeneity was observed in the outcome of AP. Therefore, we performed a meta-analysis using a random-effects model on outcomes of IAP and FOB. Furthermore, we conducted meta-regression and subgroup analyses stratified by APACHE II score at baseline among the included participants, the duration of acupuncture, and the method of acupuncture. Interestingly, the results indicated that the treatment duration was a potential source of heterogeneity in both outcome indicators (IAP and FOB). Thus, treatment duration may be a crucial factor influencing the efficacy of acupuncture in treating S-GID. Furthermore, upon reviewing the characteristics of the included studies, we found that the source of heterogeneity may arise from differences in participants’ routine treatments across studies.

The APACHE II score plays a crucial role in sepsis management by providing a reliable tool for prognostic assessment and risk stratification (34). It is a significant independent predictor of mortality in septic patients (35). Meta-analysis showed that acupuncture combined with conventional therapy was significantly better than conventional therapy alone in decreasing APACHE II score in S-GID patients. The sensitivity analysis suggested a robust result of lowering the AGI grade. However, there was significant heterogeneity among various studies, which also reduced the credibility of the results. Furthermore, subgroup analysis indicated that treatment duration may be a potential source of the heterogeneity.

Regrettably, the meta-analysis found that acupuncture combined with conventional therapy was not significantly better than conventional therapy alone in decreasing the 28-day mortality rate in S-GID patients. The sensitivity analysis showed that the meta-analysis result was not reversed. Moreover, subgroup analysis showed no significant difference in the 28-day mortality rate across subgroups. Considering that acupuncture significantly improves the severity of AGI and various gastrointestinal parameters, it theoretically should impact mortality. However, as noted in the review, the discordance between improved gastrointestinal parameters and unchanged 28-day mortality requires a critical interpretation. We propose that the lack of statistical significance in mortality may stem from several factors. First, it used small sample sizes, which may be insufficient to detect significant differences between groups. Second, sepsis is highly heterogeneous with complex subtypes and clinical variability, which might limit the generalized effect of acupuncture to specific subgroups or disease stages. Third, variations in acupuncture methods, treatment durations, and protocols could lead to inconsistent prognostic outcomes. Therefore, future research should concentrate on enlarging the sample size of randomized controlled trials (RCTs) for acupuncture treatment of S-GID. It should also carry out studies on different subtypes of S-GID using acupuncture to identify the responsive subtypes. Moreover, it should develop a reproducible and effective treatment protocol in accordance with traditional Chinese medicine theory to guarantee that its clinical value is not confined to short-term physiological effects.

However, when acupuncture was combined with conventional medicine, it effectively reduced the severity of AGI and AP in both APACHE II score subgroups, i.e., ≥22 vs. <22. Additionally, this combination therapy increased FOB. Notably, in the APACHE II score < 22 subgroup, acupuncture, in addition to conventional medicine, reduced IAP compared with conventional medicine alone; however, this effect was not observed in the APACHE II score ≥ 22 subgroup. It showed that acupuncture may be more effective in the S-GID subgroup with less severe illness, such as the subgroup with an APACHE II score < 22. However, further research is needed to confirm this.

Compared with conventional medicine alone, in the subgroups with an acupuncture duration of 5 days, the combination of acupuncture and conventional medicine increased FOB; however, it yielded negative results regarding AGI grade and APACHE II score. However, in the subgroups with an acupuncture duration of 7 days, positive results were observed for IAP, grade of AGI, FOB, and abdominal perimeter. This suggests that a minimum treatment duration of 7 days may be necessary for acupuncture to exhibit improved efficacy as an adjunctive therapy for S-AGI, which warrants further investigation. In the subgroups with an acupuncture duration of 14 days, positive results were found for IAP, AGI grade, and FOB; however, no advantages were observed regarding APACHE II score and abdominal perimeter. More regretfully, in the subgroups with an acupuncture duration of 28 days, no positive results were seen in any of the subgroups. Nonetheless, the explanation may include a limited number of studies (2, 2, and 1, respectively) within subgroups with acupuncture durations of 5, 14, or 28 days. Therefore, further research is necessary to ascertain the impact of acupuncture duration on the efficacy of S-AGI.

In the subgroup receiving manual acupuncture, the combination of acupuncture and conventional medicine resulted in a significant reduction in IAP, AGI grade, and APACHE II score compared with conventional medicine alone. Conversely, among those receiving electroacupuncture, the combination of acupuncture and conventional medicine led to a noteworthy decrease in IAP and AGI grades; however, no significant reduction in APACHE II score was observed when compared to conventional medicine alone. Notably, only three studies were included in the electroacupuncture group, highlighting the necessity for further research to validate these findings.

Regarding clinical safety, no significant increase in adverse events was reported during the treatment, and no serious adverse events occurred in either study group. Furthermore, among the studies that reported adverse events, most (7 out of 9) had no acupuncture-related accidents or adverse events. Although acupuncture seemed to be safe, the reporting of adverse events was inconsistent and often passive. Moreover, the extremely low incidence likely reflected underreporting, especially in critically ill ICU populations. This finding should be interpreted with caution. Additionally, four studies failed to report their safety results; thus, the safety profile of acupuncture was unclear in these studies. Therefore, the adverse events associated with acupuncture still need close monitoring by clinicians and standardized reporting.

To date, the specific mechanism underlying acupuncture treatment for S-GID has not been fully elucidated. Opioid use and stimulation of the parasympathetic nervous system might be involved in these mechanisms.

Opioids are extensively administered for analgesic purposes in critically ill patients; however, they are a major cause of enteroparalysis, manifesting as bowel distention and increased permeability of the colon, which can facilitate the translocation of enteric microorganisms into the bloodstream (36–38). Conversely, patients receiving acupuncture therapy exhibited reduced opioid use due to the release of endogenous opioid peptides, thereby decreasing the adverse reaction rate induced by opioids (39, 40). Thus, the beneficial effects of acupuncture therapy in S-GID may be partially ascribed to lower opioid consumption.

Stimulation of the parasympathetic nervous system (PNS) is closely associated with sepsis. Afferent signals elicited by acupoint stimulation directly modulate visceral autonomic activity following spinal cord integration. The PNS modified inflammation through cholinergic receptors of internal organs, macrophages, and lymphocytes (the cholinergic anti-inflammatory pathway) (41). Around a decade ago, researchers demonstrated that electroacupuncture at ST36 controlled systemic inflammation in sepsis via the vagus nerves modulating the production of catecholamines in the adrenal glands (42). Gastrointestinal diseases are mostly accompanied by PNS dysfunction. Current research has demonstrated that electroacupuncture at ST36 improves gut barrier dysfunction through a vagus-dependent mechanism in ischemic stroke (43), intestinal ischemia (44–46), prolonged hemorrhagic shock (47)^,^ and a colitis model (48).

In this review, the main acupuncture points selected from the included literature were local abdominal acupoints, while distal acupoints, mainly located in the Spleen and Stomach Meridians, served as adjunctive ones. The top six most frequently used acupoints were Zusanli (ST36, 12 times), Tianshu (ST25, 12 times), Zhongwan (CV12, 12 times), Xiawan (CV10, 8 times), Shangwan (CV13, 7 times), and Qihai (CV6, 7 times).

ST36 and ST25 are the two most commonly used and highly effective acupoints for gastrointestinal function modulation in acupuncture practice (49). Electrical stimulation at ST36 in a septic model could regulate inflammation and immune reactions (50–53). Zhang et al. found that stimulation at ST36 alleviated inflammatory cell infiltration in the ileal mucosa, increased the protein expression of occludin, reduced serum D-lactate levels, and enhanced the permeability of the intestinal barrier in septic rats (54). Mechanistically, in a cecal ligation and puncture (CLP)-induced sepsis model, its improvement of the intestinal mucosal barrier was attributed to the promotion of ghrelin secretion and upregulation of GSH-R expression (53). Moreover, the beneficial effect on the intestinal barrier was demonstrated to be partially spleen-dependent (50), which was further supported by the finding that electroacupuncture at ST36 reduced T-lymphocyte apoptosis and pyroptosis to blunt serum TNF-*α* levels (51). Besides, acupuncture treatment could inhibit apoptosis in intestinal tissue (52). Acupuncture at ST25 is involved in the regulation of S-GID. Stimulation at ST25 modulated T-cell immunity in the CLP model, as evidenced by reduced proportions of CD3 + CD8 + T cells and Th17 cells and increased proportions of CD3 + CD4 + T cells and Treg cells in mesenteric lymph nodes (50). Overall, the method of acupoint selection conformed to the principle of traditional Chinese medicine for invigorating the spleen and stomach.

### 4.2 Level of confidence in the evidence

The GRADE approach was employed to assess the level of confidence in the evidence presented in this study, which was categorized as having very low to moderate quality. It became clear that a significant risk of bias and inconsistency among studies contributed to the downgrading of the evidence. Furthermore, extra imprecision of the findings contributed to the downgrading of the evidence in subgroup analyses due to insufficient participant inclusion. Consequently, it is anticipated that incorporating larger RCTs with enhanced methodological quality will substantially impact the conclusions drawn from this research.

### 4.3 Risk of bias

The Cochrane ROB 2.0 tool was used to assess risk of bias. Among the 13 studies included in this analysis, only four (12, 24, 27, 30) adequately implemented concealed and blinded random allocation methods. This potential lack of methodological rigor may have led to an overestimation of the effectiveness of the treatment. Furthermore, two studies (25, 26) reported adverse events in their methodology section but did not provide relevant data in their results section, suggesting a potential risk of selective outcome reporting. Moreover, only one of the included studies (55) prospectively registered its study protocol on a clinical trial registry platform (the Chinese Clinical Trial Registry) before commencement. Considering these limitations, it is crucial to acknowledge that the observed effect size in this study might be overstated. Overall, caution should be exercised when evaluating and using these findings due to the constraints imposed by the methodology.

### 4.4 Heterogeneity among studies

Statistical heterogeneity arose from variations in clinical variations or methodological approaches across studies, potentially introducing bias (31). Identifying the sources of this heterogeneity is crucial for comprehending the study outcomes. To address this comprehensively in our systematic review, the specific population, interventions, comparators, outcomes, and study designs were meticulously defined. Additionally, subgroup analyses were conducted based on factors such as baseline APACHE II scores and the duration/methods of acupuncture administration to minimize potential clinical variations within the included studies. However, there were inevitably some clinical heterogeneities. First, although all patients included in the studies were diagnosed with S-AGI, there were variations in the severity of AGI among the final study population. Additionally, patients with clinically suspected S-AGI generally had different sources of infection, including lung, kidney, liver, and intestinal infections. Nevertheless, detailed information on the underlying sources of infection was lacking in the included studies, which also contributed to the unclear and inconsistent clinical characteristics. Sensitivity analysis results showed that heterogeneity was statistically reduced after excluding data from a specific study (29) involving the youngest participants. Second, the inclusion criteria for the intervention group combined acupuncture treatment with conventional drug therapy, which was used in the control group. However, specific details regarding the drugs administered in standard treatment were not provided in the original study. Furthermore, standard treatments for sepsis are multifaceted and depend on individual physician expertise. Given that medical practices vary across hospitals, we cannot be certain whether routine medication for sepsis was standardized or whether recommendations from clinical guidelines were consistently followed, leading to heterogeneity in treatment approaches. Regarding methodological differences between studies, two RCTs reported randomization without providing further details. Additionally, four articles described appropriate methods for concealing random sequences, whereas nine did not explicitly mention this aspect of randomization concealment. We believe that inadequate randomization procedures may have also contributed to potential heterogeneity between studies.

### 4.5 Publication bias

We examined publication bias in the IAP and APACHE II data because more than 10 studies were included. No obvious publication bias was observed. However, two of these articles (25, 26) reported adverse events in their methodology section but failed to report relevant data in their results section, indicating a potential risk of selective reporting. Moreover, only one of the included studies (12) prospectively registered its study protocol on a clinical trial registry platform (the Chinese Clinical Trial Registry) before commencement, and this study showed no obvious publication bias. Consequently, we were unable to detect selective reporting of results because the other 12 trials in this analysis did not provide information on the clinical trial registry or the study protocol. Considering these factors, we cannot disregard the presence of publishing bias.

### 4.6 Implications for clinical practice and future research

Owing to the limited availability of robust, high-quality evidence, this study was unable to draw definitive conclusions regarding acupuncture’s therapeutic effectiveness or safety profile. Considering the current uncertainty surrounding its clinical benefits, healthcare providers are recommended to exercise caution and adopt a more deliberate, individualized approach when considering acupuncture as a treatment option until the outcomes of future rigorous clinical trials are known. Moreover, although certain studies have reported adverse reactions, the overall safety profile of acupuncture is still insufficiently characterized; therefore, clinicians should remain vigilant and closely monitor for any potential adverse effects during treatment.

Currently, more high-quality studies are being conducted or planned to investigate the effectiveness of acupuncture for S-AGI, and future research should consider the methodological evaluation carried out in this study, which is as follows:

- Before including the first case, the protocol should be registered on a publicly accessible clinical trial website^2^^,^^3^ to reduce selective bias.
- Determining the sample size requires greater rigor and scientific precision.
- Correct randomization methods and detailed procedures for generating randomly assigned sequences should be adopted and reported.
- Although blinding patients in acupuncture treatment is challenging, blinding outcome assessors and statisticians should be implemented to minimize bias in outcome evaluation.
- Given the complexity of sepsis, particularly with different infection sites and etiologies that can lead to heterogeneity in prognosis, it is necessary to provide detailed information on infection sites, etiologies, and other factors to reduce clinical heterogeneity.
- To achieve optimal therapeutic effects, it is recommended that acupuncture be administered for more than 7 days.
- It is essential to thoroughly document any adverse events during the trial process to evaluate the safety profile of acupuncture.
- Following completion of clinical trials, follow-up evaluations should be conducted to assess the long-term efficacy of acupuncture.
- Considering geographical and ethnic diversity within study populations is crucial for assessing the wide applicability of acupuncture.

## 5 Conclusion

In summary, our findings indicated that acupuncture combined with conventional therapy yielded significantly greater efficacy compared with conventional therapy alone in the S-GID treatment, as evidenced by reduced IAP, AGI grade severity, AP, and APACHE II score, increased FOB, and a favorable safety profile.

However, the data on 28-day mortality did not show significant differences, which might be attributed to the insufficient sample size. Moreover, given the substandard quality of the original study, a larger number of high-quality clinical studies are needed to substantiate the clinical efficacy and safety of acupuncture in S-GID.

## Data Availability

The original contributions presented in the study are included in the article/Supplementary material, further inquiries can be directed to the corresponding author.

## Footnotes

1
https://gradepro.org/

2
https://www.chictr.org.cn

3
https://clinicaltrials.gov

## Abbreviations

AGI: acute gastrointestinal injury
AP: abdominal perimeter measurements
APACHE II: Acute Physiology and Chronic Health Evaluation II score
BMHRCP: Beijing Municipal Hospital Research Cultivation Program
CENTRA: Cochrane Central Register of Controlled Trial
CHiCTR: the Chinese Clinical Trials Registry
CI: confidence interval
Cqvip: VIP information resource integration service platform
EA: electro-acupuncture
FOB: frequency of borborygmus
*I* ^2^: index statistic
IAP: intra-abdominal pressure
LTIEP: the Lanzhou Talent Innovation and Entrepreneurship Project
MA: manual acupuncture
MD: Mean difference
MODS: multiple organ dysfunction syndrome
PRISMA: Preferred Reporting Items for Systematic Reviews and Meta-Analyses
RCTs: randomized controlled trials
RoB 2: The Cochrane Collaboration Risk of Bias 2.0
RR: relative risk
S-GID: Septic gastrointestinal dysfunction
Sinomed: Chinese biomedical literature service system
SMD: standard mean difference
SPKRDP: Shandong Provincial Key Research and Development Program
SPNSF: Shandong Provincial Natural Science Foundation
STCMDTMIPCP: Shanghai Traditional Chinese Medicine Diagnosis and Treatment Model Innovation Pilot Construction Project
SUTCMAYITCWHFYP: Shanghai University of Traditional Chinese Medicine Affiliated Yueyang Integrated Traditional Chinese and Western Medicine Hospital Fund Youth Project
TCM: Traditional Chinese Medicine
ZPATCM: the Zhejiang Provincial Administration of Traditional Chinese Medicine, China
CNKI: China National Knowledge Infrastructure
