# Supplementary material for: Efficacy and safety of acupuncture for septic gastrointestinal dysfunction: a systematic review and meta-analysis of randomized controlled trials
Source: Front Med (Lausanne). 2026 Feb 26;13:1680999. doi: 10.3389/fmed.2026.1680999 (PMC12979551; doi:10.3389/fmed.2026.1680999)
Supplement: Supplementary file 1 [file Data_Sheet_1.pdf]

Supplementary material

Table of contents

目录

PRISMA 2020 checklist 1
Supplementary File S1. Definition of AGI grade. 7
Supplementary File S2. Search strategies for databases. 8
Supplementary File S3. A list of excluded studies by reading title and abstract. 33
Supplementary File S4. A list of excluded studies by reading full text. 38
Supplementary File S5. Acupuncture prescription in the included studies. 39
Supplementary File S6. Results of subgroup analysis 42
Supplementary File S7. Quality of evidence 50

PRISMA 2020 checklist

Section and Topic	Item #	Checklist item	Location where item is reported
**TITLE**			
Title	1	Identify the report as a systematic review.	P1
**ABSTRACT**			
Abstract	2	See the PRISMA 2020 for Abstracts checklist.	P2
**INTRODUCTION**			
Rationale	3	Describe the rationale for the review in the context of existing knowledge.	P3-4

Section and Topic	Item #	Checklist item	Location where item is reported
Objectives	4	Provide an explicit statement of the objective(s) or question(s) the review addresses.	P4
**METHODS**			
Eligibility criteria	5	Specify the inclusion and exclusion criteria for the review and how studies were grouped for the syntheses.	P4-8
Information sources	6	Specify all databases, registers, websites, organisations, reference lists and other sources searched or consulted to identify studies. Specify the date when each source was last searched or consulted.	P6
Search strategy	7	Present the full search strategies for all databases, registers and websites, including any filters and limits used.	P6, Supplementary File S2
Selection process	8	Specify the methods used to decide whether a study met the inclusion criteria of the review, including how many reviewers screened each record and each report retrieved, whether they worked independently, and if applicable, details of automation tools used in the process.	P6
Data collection process	9	Specify the methods used to collect data from reports, including how many reviewers collected data from each report, whether they worked independently, any processes for obtaining or confirming data from study investigators, and if applicable, details of automation tools used in the process.	P6
Data items	10a	List and define all outcomes for which data were sought. Specify whether all results that were compatible with each outcome domain in each study were sought (e.g. for all	P4-5

Section and Topic	Item #	Checklist item	Location where item is reported
		measures, time points, analyses), and if not, the methods used to decide which results to collect.	
	10b	List and define all other variables for which data were sought (e.g. participant and intervention characteristics, funding sources). Describe any assumptions made about any missing or unclear information.	P5-6
Study risk of bias assessment	11	Specify the methods used to assess risk of bias in the included studies, including details of the tool(s) used, how many reviewers assessed each study and whether they worked independently, and if applicable, details of automation tools used in the process.	P6
Effect measures	12	Specify for each outcome the effect measure(s) (e.g. risk ratio, mean difference) used in the synthesis or presentation of results.	P7
Synthesis methods	13a	Describe the processes used to decide which studies were eligible for each synthesis (e.g. tabulating the study intervention characteristics and comparing against the planned groups for each synthesis (item #5)).	P7
	13b	Describe any methods required to prepare the data for presentation or synthesis, such as handling of missing summary statistics, or data conversions.	P7
	13c	Describe any methods used to tabulate or visually display results of individual studies and syntheses.	P7
	13d	Describe any methods used to synthesize results and provide a rationale for the choice(s). If meta-analysis was performed, describe the model(s), method(s) to identify the presence and extent of statistical heterogeneity, and software package(s) used.	P7

Section and Topic	Item #	Checklist item	Location where item is reported
	13e	Describe any methods used to explore possible causes of heterogeneity among study results (e.g. subgroup analysis, meta-regression).	P7
	13f	Describe any sensitivity analyses conducted to assess robustness of the synthesized results.	P7-8
Reporting bias assessment	14	Describe any methods used to assess risk of bias due to missing results in a synthesis (arising from reporting biases).	P8
Certainty assessment	15	Describe any methods used to assess certainty (or confidence) in the body of evidence for an outcome.	P8
**RESULTS**			
Study selection	16a	Describe the results of the search and selection process, from the number of records identified in the search to the number of studies included in the review, ideally using a flow diagram.	P8-9
	16b	Cite studies that might appear to meet the inclusion criteria, but which were excluded, and explain why they were excluded.	P9 and Supplementary File S3 and S4
Study characteristics	17	Cite each included study and present its characteristics.	P8-15
Risk of bias in studies	18	Present assessments of risk of bias for each included study.	P16

Section and Topic	Item #	Checklist item	Location where item is reported
Results of individual studies	19	For all outcomes, present, for each study: (a) summary statistics for each group (where appropriate) and (b) an effect estimate and its precision (e.g. confidence/credible interval), ideally using structured tables or plots.	Table 1 and 2
Results of syntheses	20a	For each synthesis, briefly summarise the characteristics and risk of bias among contributing studies.	P16-19
	20b	Present results of all statistical syntheses conducted. If meta-analysis was done, present for each the summary estimate and its precision (e.g. confidence/credible interval) and measures of statistical heterogeneity. If comparing groups, describe the direction of the effect.	P16-19
	20c	Present results of all investigations of possible causes of heterogeneity among study results.	P16-19
	20d	Present results of all sensitivity analyses conducted to assess the robustness of the synthesized results.	P16-19
Reporting biases	21	Present assessments of risk of bias due to missing results (arising from reporting biases) for each synthesis assessed.	P16, P18
Certainty of evidence	22	Present assessments of certainty (or confidence) in the body of evidence for each outcome assessed.	P20
**DISCUSSION**			
Discussion	23a	Provide a general interpretation of the results in the context of other evidence.	P20-25
	23b	Discuss any limitations of the evidence included in the review.	P20-25

Section and Topic	Item #	Checklist item	Location where item is reported
	23c	Discuss any limitations of the review processes used.	P20-25
	23d	Discuss implications of the results for practice, policy, and future research.	P25-26
**OTHER INFORMATION**			
Registration and protocol	24a	Provide registration information for the review, including register name and registration number, or state that the review was not registered.	P4
	24b	Indicate where the review protocol can be accessed, or state that a protocol was not prepared.	P4
	24c	Describe and explain any amendments to information provided at registration or in the protocol.	P4
Support	25	Describe sources of financial or non-financial support for the review, and the role of the funders or sponsors in the review.	P29
Competing interests	26	Declare any competing interests of review authors.	P29
Availability of data, code and other materials	27	Report which of the following are publicly available and where they can be found: template data collection forms; data extracted from included studies; data used for all analyses; analytic code; any other materials used in the review.	The Supplementary Material

From: Page MJ, McKenzie JE, Bossuyt PM, Boutron I, Hoffmann TC, Mulrow CD, et al. The PRISMA 2020 statement: an updated guideline for reporting systematic reviews. BMJ 2021;372:n71. doi: 10.1136/bmj.n71

For more information, visit: http://www.prisma-statement.org/

Supplementary File S1. Definition of AGI grade.

Table s1 Definition of AGI grade

AGI grade	Definiction	Examples
Grade I (risk of developing GI dysfunction or failure)	The function of the GI tract is partially impaired, expressed as GI symptoms related to a known cause and perceived as transient, which expectedly has temporary and self-limiting nature.	Postoperative nausea and/or vomiting during the first days after abdominal surgery, postoperative absence of bowel sounds, diminished bowel motility in the early phase of shock.
Grade II (gastrointestinal dysfunction)	The GI tract is not able to perform digestion and absorption adequately to satisfy the nutrient and fluid requirements of the body.	Gastroparesis with high gastric residuals or reflux, paralysis of the lower GI tract, diarrhoea, intra-abdominal hypertension (IAH) grade I (intra-abdominal pressure (IAP)12–15 mmHg), visible blood in gastric contentor stool. Feeding intolerance is present if at least 20 kcal/kg BW/day via enteral route cannot bereached within 72 h of feeding attempt.
Grade III (gastrointestinal failure)	Loss of GI function, where restoration of GI function is not achieved despite interventions and the general condition is not improving.	Gastroparesis with high gastric residuals or reflux, paralysis of the lower GI tract, diarrhoea, intra-abdominal hypertension (IAH) grade I (intra-abdominal pressure (IAP) 12–15 mmHg), visible blood in gastric content or stool. Feeding intolerance is present if at least 20 kcal/kg /day via enteral route cannot be reached within 72 h of feeding attempt.
Grade IV (gastrointestinal failure with severe impact on distant organ function	AGI has pro-gressed to become directly and immediately life-threatening, with worsening of MODS and shock.	Bowel ischaemia with necrosis, GI bleeding leading to haemorrhagic shock, Ogilvie's syndrome, abdominal compartment syndrome(ACS) requiring decompression.

Tips: 1. Intra-abdominal hypertension: The intra abdominal pressure continuously or repeatedly rises to ≥ 12 mmHg.

2. Abdominal compartment syndrome (ACS): A continuous intra-abdominal pressure > 20 mmHg (with or without an abdominal perfusion pressure < 60 mmHg), along with the presence of new organ dysfunction/failure.

3. Grading of intra-abdominal hypertension: Grade I: Intra-abdominal pressure ranges from 12 to 15 mmHg; Grade II: Intra-abdominal pressure ranges from 16 to 20 mmHg; Grade III: Intra-abdominal pressure ranges from 21 to 25 mmHg;

Grade IV: Intra-abdominal pressure > 25 mmHg.

4. High gastric residuals: It is defined as a single gastric fluid aspiration exceeding 250 ml or 500 ml/6 h.

Supplementary File S2. Search strategies for databases.

2.1 PubMed (https://pubmed.ncbi.nlm.nih.gov/advanced/)
Search date:September 24, 2023 Beijing time

Search	Add to Query box	Query	Iterms found	Time
#5	(((Disease, Gastrointestinal[Title/Abstract]) OR (Diseases, Gastrointestinal[Title/Abstract]) OR (Gastrointestinal Disease[Title/Abstract]) OR (Gastrointestinal Disorders[Title/Abstract]) OR (Gastrointestinal Disorder[Title/Abstract]) OR (Functional Gastrointestinal Disorders[Title/Abstract]) OR (Functional Gastrointestinal Disorder[Title/Abstract]) OR (Gastrointestinal Disorder, Functional[Title/Abstract]) OR (Gastrointestinal Disorders, Functional[Title/Abstract]) OR (Cholera Infantum[Title/Abstract]) OR (gastrointestinal dysfunction[Title/Abstract]) OR (acute gastrointestinal injury[Title/Abstract]) OR ("Gastrointestinal Diseases"[Mesh])) AND ((Bloodstream Infection[Title/Abstract]) OR (Bloodstream Infections[Title/Abstract]) OR (Infection, Bloodstream[Title/Abstract]) OR (Pyemia[Title/Abstract]) OR (Pyemias[Title/Abstract]) OR (Pyohemia[Title/Abstract]) OR (Pyohemias[Title/Abstract]) OR (Pyaemia[Title/Abstract]) OR (Pyaemias[Title/Abstract]) OR (Septicemia[Title/Abstract]) OR (Septicemias[Title/Abstract]) OR	("disease gastrointestinal"[Title/Abstract] OR "diseases gastrointestinal"[Title/Abstract] OR "gastrointestinal disease"[Title/Abstract] OR "gastrointestinal disorders"[Title/Abstract] OR "gastrointestinal disorder"[Title/Abstract] OR "functional gastrointestinal disorders"[Title/Abstract] OR "functional gastrointestinal disorder"[Title/Abstract] OR "gastrointestinal disorder functional"[Title/Abstract] OR "gastrointestinal disorders functional"[Title/Abstract] OR "cholera infantum"[Title/Abstract] OR "gastrointestinal dysfunction"[Title/Abstract] OR "acute gastrointestinal injury"[Title/Abstract] OR "Gastrointestinal Diseases"[MeSH Terms]) AND ("bloodstream infection"[Title/Abstract] OR "bloodstream infections"[Title/Abstract] OR "infection bloodstream"[Title/Abstract] OR "Pyemia"[Title/Abstract] OR "Pyemias"[Title/Abstract] OR "Pyohemia"[Title/Abstract] OR "Pyohemias"[Title/Abstract] OR "Pyaemia"[Title/Abstract] OR "Septicemia"[Title/Abstract] OR "Septicemias"[Title/Abstract] OR "poisoning blood"[Title/Abstract] OR	7	23:46:14

(Poisoning, Blood[Title/Abstract]) OR (Blood Poisoning[Title/Abstract]) OR (Blood Poisonings[Title/Abstract]) OR (Poisonings, Blood[Title/Abstract]) OR (Severe Sepsis[Title/Abstract]) OR (Sepsis, Severe[Title/Abstract]) OR (Sepsis[Mesh]))) AND ((Pharmacopuncture[Title/Abstract]) OR (Acupuncture Treatment[Title/Abstract]) OR (Acupuncture Treatments[Title/Abstract]) OR (Treatment, Acupuncture[Title/Abstract]) OR (Therapy, Acupuncture[Title/Abstract]) OR (Pharmacoacupuncture Treatment[Title/Abstract]) OR (Treatment, Pharmacoacupuncture[Title/Abstract]) OR (Pharmacoacupuncture Therapy[Title/Abstract]) OR (Therapy, Pharmacoacupuncture[Title/Abstract]) OR (Acupotomy[Title/Abstract]) OR (Acupotomies[Title/Abstract]) OR ("Acupuncture Therapy"[Mesh]) OR ("acupuncture"[MeSH Terms]))	"blood poisoning"[Title/Abstract] OR "blood poisonings"[Title/Abstract] OR (("poisoned"[All Fields] OR "Poisoning"[MeSH Terms] OR "Poisoning"[All Fields] OR "Poisonings"[All Fields] OR "Poisoning"[MeSH Subheading] OR "poisonous"[All Fields] OR "poisons"[Pharmacological Action] OR "poisons"[MeSH Terms] OR "poisons"[All Fields] OR "poison"[All Fields]) AND "Blood"[Title/Abstract]) OR "severe sepsis"[Title/Abstract] OR "sepsis severe"[Title/Abstract] OR "Sepsis"[MeSH Terms]) AND ("Pharmacopuncture"[Title/Abstract] OR "acupuncture treatment"[Title/Abstract] OR "acupuncture treatments"[Title/Abstract] OR "treatment acupuncture"[Title/Abstract] OR "therapy acupuncture"[Title/Abstract] OR "pharmacoacupuncture treatment"[Title/Abstract] OR (("therapeutics"[MeSH Terms] OR "therapeutics"[All Fields] OR "Treatments"[All Fields] OR "Therapy"[MeSH Subheading] OR "Therapy"[All Fields] OR "Treatment"[All Fields] OR "treatment s"[All Fields]) AND "Pharmacoacupuncture"[Title/Abstract]) OR "pharmacoacupuncture therapy"[Title/Abstract] OR (("therapeutics"[MeSH Terms] OR "therapeutics"[All Fields] OR "therapies"[All Fields] OR "Therapy"[MeSH Subheading] OR "Therapy"[All Fields] OR "therapy s"[All Fields] OR "therapys"[All Fields]) AND "Pharmacoacupuncture"[Title/Abstract]) OR "Acupotomy"[Title/Abstract] OR "Acupotomies"[Title/Abstract] OR "Acupuncture Therapy"[MeSH Terms] OR "Acupuncture"[MeSH Terms])

#4	(Pharmacopuncture[Title/Abstract]) OR (Acupuncture Treatment[Title/Abstract]) OR (Acupuncture Treatments[Title/Abstract]) OR (Treatment, Acupuncture[Title/Abstract]) OR (Therapy, Acupuncture[Title/Abstract]) OR (Pharmacoacupuncture Treatment[Title/Abstract]) OR (Treatment, Pharmacoacupuncture[Title/Abstract]) OR (Pharmacoacupuncture Therapy[Title/Abstract]) OR (Therapy, Pharmacoacupuncture[Title/Abstract]) OR (Acupotomy[Title/Abstract]) OR (Acupotomies[Title/Abstract]) OR ("Acupuncture Therapy"[Mesh]) OR ("acupuncture"[MeSH Terms])	"Pharmacopuncture"[Title/Abstract] OR "acupuncture treatment"[Title/Abstract] OR "acupuncture treatments"[Title/Abstract] OR "treatment acupuncture"[Title/Abstract] OR "therapy acupuncture"[Title/Abstract] OR "pharmacoacupuncture treatment"[Title/Abstract] OR (("therapeutics"[MeSH Terms] OR "therapeutics"[All Fields] OR "Treatments"[All Fields] OR "Therapy"[MeSH Subheading] OR "Therapy"[All Fields] OR "Treatment"[All Fields] OR "treatment s"[All Fields]) AND "Pharmacoacupuncture"[Title/Abstract]) OR "pharmacoacupuncture therapy"[Title/Abstract] OR (("therapeutics"[MeSH Terms] OR "therapeutics"[All Fields] OR "therapies"[All Fields] OR "Therapy"[MeSH Subheading] OR "Therapy"[All Fields] OR "therapy s"[All Fields] OR "therapys"[All Fields]) AND "Pharmacoacupuncture"[Title/Abstract]) OR "Acupotomy"[Title/Abstract] OR "Acupotomies"[Title/Abstract] OR "Acupuncture Therapy"[MeSH Terms] OR "Acupuncture"[MeSH Terms]	31,244	23:46:05
#3	((Disease, Gastrointestinal[Title/Abstract]) OR (Diseases, Gastrointestinal[Title/Abstract]) OR (Gastrointestinal Disease[Title/Abstract]) OR (Gastrointestinal Disorders[Title/Abstract]) OR (Gastrointestinal Disorder[Title/Abstract]) OR (Functional Gastrointestinal Disorders[Title/Abstract]) OR (Functional Gastrointestinal Disorder[Title/Abstract]) OR (Gastrointestinal Disorder,	("disease gastrointestinal"[Title/Abstract] OR "diseases gastrointestinal"[Title/Abstract] OR "gastrointestinal disease"[Title/Abstract] OR "gastrointestinal disorders"[Title/Abstract] OR "gastrointestinal disorder"[Title/Abstract] OR "functional gastrointestinal disorders"[Title/Abstract] OR "functional gastrointestinal disorder"[Title/Abstract] OR "gastrointestinal disorder	6,556	23:43:21

	Functional[Title/Abstract]) OR (Gastrointestinal Disorders, Functional[Title/Abstract]) OR (Cholera Infantum[Title/Abstract]) OR (gastrointestinal dysfunction[Title/Abstract]) OR (acute gastrointestinal injury[Title/Abstract]) OR ("Gastrointestinal Diseases"[Mesh])) AND ((Bloodstream Infection[Title/Abstract]) OR (Bloodstream Infections[Title/Abstract]) OR (Infection, Bloodstream[Title/Abstract]) OR (Pyemia[Title/Abstract]) OR (Pyemias[Title/Abstract]) OR (Pyohemia[Title/Abstract]) OR (Pyohemias[Title/Abstract]) OR (Pyaemia[Title/Abstract]) OR (Pyaemias[Title/Abstract]) OR (Septicemia[Title/Abstract]) OR (Septicemias[Title/Abstract]) OR (Poisoning, Blood[Title/Abstract]) OR (Blood Poisoning[Title/Abstract]) OR (Blood Poisonings[Title/Abstract]) OR (Poisonings, Blood[Title/Abstract]) OR (Severe Sepsis[Title/Abstract]) OR (Sepsis, Severe[Title/Abstract]) OR (Sepsis[Mesh]))	functional"[Title/Abstract] OR "gastrointestinal disorders functional"[Title/Abstract] OR "cholera infantum"[Title/Abstract] OR "gastrointestinal dysfunction"[Title/Abstract] OR "acute gastrointestinal injury"[Title/Abstract] OR "Gastrointestinal Diseases"[MeSH Terms]) AND ("bloodstream infection"[Title/Abstract] OR "bloodstream infections"[Title/Abstract] OR "infection bloodstream"[Title/Abstract] OR "Pyemia"[Title/Abstract] OR "Pyemias"[Title/Abstract] OR "Pyohemia"[Title/Abstract] OR "Pyohemias"[Title/Abstract] OR "Pyaemia"[Title/Abstract] OR "Septicemia"[Title/Abstract] OR "Septicemias"[Title/Abstract] OR "poisoning blood"[Title/Abstract] OR "blood poisoning"[Title/Abstract] OR "blood poisonings"[Title/Abstract] OR (("poisoned"[All Fields] OR "Poisoning"[MeSH Terms] OR "Poisoning"[All Fields] OR "Poisonings"[All Fields] OR "Poisoning"[MeSH Subheading] OR "poisonous"[All Fields] OR "poisons"[Pharmacological Action] OR "poisons"[MeSH Terms] OR "poisons"[All Fields] OR "poison"[All Fields]) AND "Blood"[Title/Abstract]) OR "severe sepsis"[Title/Abstract] OR "sepsis severe"[Title/Abstract] OR "Sepsis"[MeSH Terms])		
#2	(Disease, Gastrointestinal[Title/Abstract]) OR (Diseases, Gastrointestinal[Title/Abstract]) OR (Gastrointestinal Disease[Title/Abstract]) OR (Gastrointestinal Disorders[Title/Abstract]) OR (Gastrointestinal	"disease gastrointestinal"[Title/Abstract] OR "diseases gastrointestinal"[Title/Abstract] OR "gastrointestinal disease"[Title/Abstract] OR "gastrointestinal disorders"[Title/Abstract] OR "gastrointestinal	1,088,683	23:06:24

	Disorder[Title/Abstract]) OR (Functional Gastrointestinal Disorders[Title/Abstract]) OR (Functional Gastrointestinal Disorder[Title/Abstract]) OR (Gastrointestinal Disorder, Functional[Title/Abstract]) OR (Gastrointestinal Disorders, Functional[Title/Abstract]) OR (Cholera Infantum[Title/Abstract]) OR (gastrointestinal dysfunction[Title/Abstract]) OR (acute gastrointestinal injury[Title/Abstract]) OR ("Gastrointestinal Diseases"[Mesh])	disorder"[Title/Abstract] OR "functional gastrointestinal disorders"[Title/Abstract] OR "functional gastrointestinal disorder"[Title/Abstract] OR "gastrointestinal disorder functional"[Title/Abstract] OR "gastrointestinal disorders functional"[Title/Abstract] OR "cholera infantum"[Title/Abstract] OR "gastrointestinal dysfunction"[Title/Abstract] OR "acute gastrointestinal injury"[Title/Abstract] OR "Gastrointestinal Diseases"[MeSH Terms]		
#1	(Bloodstream Infection[Title/Abstract]) OR (Bloodstream Infections[Title/Abstract]) OR (Infection, Bloodstream[Title/Abstract]) OR (Pyemia[Title/Abstract]) OR (Pyemias[Title/Abstract]) OR (Pyohemia[Title/Abstract]) OR (Pyohemias[Title/Abstract]) OR (Pyaemia[Title/Abstract]) OR (Pyaemias[Title/Abstract]) OR (Septicemia[Title/Abstract]) OR (Septicemias[Title/Abstract]) OR (Poisoning, Blood[Title/Abstract]) OR (Blood Poisoning[Title/Abstract]) OR (Blood Poisonings[Title/Abstract]) OR (Poisonings, Blood[Title/Abstract]) OR (Severe Sepsis[Title/Abstract]) OR (Sepsis, Severe[Title/Abstract]) OR (Sepsis[Mesh])	"bloodstream infection"[Title/Abstract] OR "bloodstream infections"[Title/Abstract] OR "infection bloodstream"[Title/Abstract] OR "Pyemia"[Title/Abstract] OR "Pyemias"[Title/Abstract] OR "Pyohemia"[Title/Abstract] OR "Pyohemias"[Title/Abstract] OR "Pyaemia"[Title/Abstract] OR "Septicemia"[Title/Abstract] OR "Septicemias"[Title/Abstract] OR "poisoning blood"[Title/Abstract] OR "blood poisoning"[Title/Abstract] OR "blood poisonings"[Title/Abstract] OR (("poisoned"[All Fields] OR "Poisoning"[MeSH Terms] OR "Poisoning"[All Fields] OR "Poisonings"[All Fields] OR "Poisoning"[MeSH Subheading] OR "poisonous"[All Fields] OR "poisons"[Pharmacological Action] OR "poisons"[MeSH Terms] OR "poisons"[All Fields] OR "poison"[All Fields]) AND "Blood"[Title/Abstract]) OR "severe sepsis"[Title/Abstract] OR "sepsis severe"[Title/Abstract] OR "Sepsis"[MeSH Terms]	189,199	22:56:23

NIH National Library of Medicine
National Center for Biotechnology Information

Log in

PubMed Advanced Search Builder

PubMed®
User Guide

Add terms to the query box

All Fields | Enter a search term | ADD

Show Index

Query box

Enter / edit your search query here

Search

History and Search Details

Download Delete

Search	Actions	Details	Query	Results	Time
#5	•••	! >	Search: (((Disease, Gastrointestinal[Title/Abstract]) OR (Diseases, Gastrointestinal[Title/Abstract]) OR (Gastrointestinal Disease[Title/Abstract]) OR (Gastrointestinal Disorders[Title/Abstract]) OR (Gastrointestinal Disorder[Title/Abstract]) OR (Functional Gastrointestinal Disorders[Title/Abstract]) OR (Functional Gastrointestinal Disorder[Title/Abstract]) OR (Gastrointestinal Disorder, Functional[Title/Abstract]) OR (Gastrointestinal Disorders, Functional[Title/Abstract]) OR (Cholera Infantum[Title/Abstract]) OR (gastrointestinal dysfunction[Title/Abstract]) OR (acute gastrointestinal injury[Title/Abstract]) OR ("Gastrointestinal Diseases"[Mesh])) AND ((Bloodstream Infection[Title/Abstract]) OR (Bloodstream Infections[Title/Abstract]) OR (Infection, Bloodstream[Title/Abstract]) OR (Pyemia[Title/Abstract]) OR (Pyemias[Title/Abstract]) OR (Pyohemia[Title/Abstract]) OR (Pyohemias[Title/Abstract]) OR (Pyaemia[Title/Abstract]) OR (Pyaemias[Title/Abstract]) OR (Septicemia[Title/Abstract]) OR (Septicemias[Title/Abstract]) OR (Poisoning, Blood[Title/Abstract]) OR (Blood Poisoning[Title/Abstract]) OR (Blood Poisonings[Title/Abstract]) OR (Poisonings, Blood[Title/Abstract]) OR (Severe Sepsis[Title/Abstract]) OR (Sepsis, Severe[Title/Abstract]) OR (Sepsis[Mesh]))) AND ((Pharmacopuncture[Title/Abstract]) OR (Acupuncture Treatment[Title/Abstract]) OR (Acupuncture Treatments[Title/Abstract]) OR (Treatment, Acupuncture[Title/Abstract]) OR (Therapy, Acupuncture[Title/Abstract]) OR (Pharmacoacupuncture Treatment[Title/Abstract]) OR (Treatment, Pharmacoacupuncture[Title/Abstract]) OR (Pharmacoacupuncture Therapy[Title/Abstract]) OR (Therapy, Pharmacoacupuncture[Title/Abstract]) OR (Acupotomy[Title/Abstract]) OR (Acupotomies[Title/Abstract]) OR ("Acupuncture Therapy"[Mesh]) OR ("acupuncture"[MeSH Terms]))	7	11:46:14
#4	•••	>	Search: (Pharmacopuncture[Title/Abstract]) OR (Acupuncture Treatment[Title/Abstract]) OR (Acupuncture Treatments[Title/Abstract]) OR (Treatment, Acupuncture[Title/Abstract]) OR (Therapy, Acupuncture[Title/Abstract]) OR (Pharmacoacupuncture Treatment[Title/Abstract]) OR (Treatment, Pharmacoacupuncture[Title/Abstract]) OR (Pharmacoacupuncture Therapy[Title/Abstract]) OR (Therapy, Pharmacoacupuncture[Title/Abstract]) OR (Acupotomy[Title/Abstract]) OR (Acupotomies[Title/Abstract]) OR ("Acupuncture Therapy"[Mesh]) OR ("acupuncture"[MeSH Terms])	31,244	11:46:05

Search	Actions	Details	Query	Results	Time
#3	•••	❗ >	Search: ((Disease, Gastrointestinal[Title/Abstract]) OR (Diseases, Gastrointestinal[Title/Abstract]) OR (Gastrointestinal Disease[Title/Abstract]) OR (Gastrointestinal Disorders[Title/Abstract]) OR (Gastrointestinal Disorder[Title/Abstract]) OR (Functional Gastrointestinal Disorders[Title/Abstract]) OR (Functional Gastrointestinal Disorder[Title/Abstract]) OR (Gastrointestinal Disorder, Functional[Title/Abstract]) OR (Gastrointestinal Disorders, Functional[Title/Abstract]) OR (Cholera Infantum[Title/Abstract]) OR (gastrointestinal dysfunction[Title/Abstract]) OR (acute gastrointestinal injury[Title/Abstract]) OR ("Gastrointestinal Diseases"[Mesh])) AND ((Bloodstream Infection[Title/Abstract]) OR (Bloodstream Infections[Title/Abstract]) OR (Infection, Bloodstream[Title/Abstract]) OR (Pyemia[Title/Abstract]) OR (Pyemias[Title/Abstract]) OR (Pyohemia[Title/Abstract]) OR (Pyohemias[Title/Abstract]) OR (Pyaemia[Title/Abstract]) OR (Pyaemias[Title/Abstract]) OR (Septicemia[Title/Abstract]) OR (Septicemias[Title/Abstract]) OR (Poisoning, Blood[Title/Abstract]) OR (Blood Poisoning[Title/Abstract]) OR (Blood Poisonings[Title/Abstract]) OR (Poisonings, Blood[Title/Abstract]) OR (Severe Sepsis[Title/Abstract]) OR (Sepsis, Severe[Title/Abstract]) OR (Sepsis[Mesh]))	6,556	11:43:21
#2	•••	>	Search: (Disease, Gastrointestinal[Title/Abstract]) OR (Diseases, Gastrointestinal[Title/Abstract]) OR (Gastrointestinal Disease[Title/Abstract]) OR (Gastrointestinal Disorders[Title/Abstract]) OR (Gastrointestinal Disorder[Title/Abstract]) OR (Functional Gastrointestinal Disorders[Title/Abstract]) OR (Functional Gastrointestinal Disorder[Title/Abstract]) OR (Gastrointestinal Disorder, Functional[Title/Abstract]) OR (Gastrointestinal Disorders, Functional[Title/Abstract]) OR (Cholera Infantum[Title/Abstract]) OR (gastrointestinal dysfunction[Title/Abstract]) OR (acute gastrointestinal injury[Title/Abstract]) OR ("Gastrointestinal Diseases"[Mesh])	1,088,683	11:06:24
#1	•••	❗ >	Search: (Bloodstream Infection[Title/Abstract]) OR (Bloodstream Infections[Title/Abstract]) OR (Infection, Bloodstream[Title/Abstract]) OR (Pyemia[Title/Abstract]) OR (Pyemias[Title/Abstract]) OR (Pyohemia[Title/Abstract]) OR (Pyohemias[Title/Abstract]) OR (Pyaemia[Title/Abstract]) OR (Pyaemias[Title/Abstract]) OR (Septicemia[Title/Abstract]) OR (Septicemias[Title/Abstract]) OR (Poisoning, Blood[Title/Abstract]) OR (Blood Poisoning[Title/Abstract]) OR (Blood Poisonings[Title/Abstract]) OR (Poisonings, Blood[Title/Abstract]) OR (Severe Sepsis[Title/Abstract]) OR (Sepsis, Severe[Title/Abstract]) OR (Sepsis[Mesh])	189,199	10:56:23

Showing 1 to 5 of 5 entries

(1) Ban L, Pu Y, Huang H, You B, Chen W, Wang Y. Acupuncture Enhances Gastrointestinal Motility and Improves Autonomic Nervous Function in Patients with Septic Gastrointestinal Dysfunction. Comput Math Methods Med. 2022 Sep 21;2022:1653290. doi: 10.1155/2022/1653290. PMID: 36188104; PMCID: PMC9519294.

(2) Meng JB, Jiao YN, Zhang G, Xu XJ, Ji CL, Hu MH, Lai ZZ, Zhang M. Electroacupuncture Improves Intestinal Dysfunction in Septic Patients: A Randomised Controlled Trial. Biomed Res Int. 2018 Jun 26;2018:8293594. doi: 10.1155/2018/8293594. PMID: 30046610; PMCID: PMC6038666.

(3) Li HF, Hu GQ, Liu WW. [Clinical trials of acupuncture of Jiaji (EX-B2) for treatment of gastrointestinal dysfunction in sepsis patients]. Zhen Ci Yan Jiu. 2019 Jan 25;44(1):43-6. Chinese. doi: 10.13702/j.1000-0607.170579. PMID:30773861.

(4) Li HF, Hu GQ, Liu WW, Chen W. [Clinical observation on the inflammatory indexes in septic gastrointestinal dysfunction treated with acupuncture at Jiaji (EX-B 2)]. Zhongguo Zhen Jiu. 2019 Oct 12;39(10):1055-8. Chinese. doi: 10.13703/j.0255-2930.2019.10.006. PMID: 31621256.

(5) Liu H, Zhu J, Ni HB, Hu XX. [Transcutaneous electrical acupoint stimulation for early enteral nutrition tolerance in patients with sepsis of gastrointestinal dysfunction: a multi-center randomized controlled

trial]. Zhongguo Zhen Jiu. 2020 Mar 12;40(3):229-33. Chinese. doi: 10.13703/j.0255-2930.20190426-0003. PMID: 32270631.
(6) Zhao F, Zeng J, Xian S, Lin X, Liu K, Lu L, Lin G, Wang S. Acupuncture improves paralytic ileus secondary to sepsis: a case report. Acupunct Med. 2019 Dec;37(6):372-374. doi: 10.1177/0964528419883279. Epub 2019 Nov 13. PMID: 31722545.
(7) Pacheco-Cerrato IM, Cobos-Moreno P, Castro-Avalos MA, Fernandez-la-Villa J, Morán-Cortés JF, Moran JM. Comment on "Electroacupuncture Improves Intestinal ysfunction in Septic Patients: A Randomised Controlled Trial". Biomed Res Int. 2021 Feb 17;2021:6487272. doi: 10.1155/2021/6487272. PMID: 33688494; PMCID: PMC7914076.

2.2 Embase (https://www.embase.com/)
Search date:September 25, 2023

No.	Query	Results
#5	#3 AND #4	6
#4	pharmacopuncture:ti,ab,kw OR 'acupuncture treatment':ti,ab,kw OR 'acupuncture treatments':ti,ab,kw OR 'treatment, acupuncture':ti,ab,kw OR 'therapy, acupuncture':ti,ab,kw OR 'pharmacoacupuncture treatment':ti,ab,kw OR 'treatment, pharmacoacupuncture':ti,ab,kw OR 'pharmacoacupuncture therapy':ti,ab,kw OR 'therapy, pharmacoacupuncture':ti,ab,kw	39,873

	OR acupotomy:ti,ab,kw OR acupotomies:ti,ab,kw OR 'acupuncture therapy':ti,ab,kw OR acupuncture:ti,ab,kw	
#3	#1 AND #2	461
#2	'disease, gastrointestinal':ti,ab,kw OR 'diseases, gastrointestinal':ti,ab,kw OR 'gastrointestinal disorders':ti,ab,kw OR 'gastrointestinal disorder':ti,ab,kw OR 'functional gastrointestinal disorders':ti,ab,kw OR 'gastrointestinal disease':ti,ab,kw OR 'functional gastrointestinal disorder':ti,ab,kw OR 'gastrointestinal disorder, functional':ti,ab,kw OR 'gastrointestinal disorders, functional':ti,ab,kw OR 'cholera	34,594

	infantum':ti,ab,kw OR 'gastrointestinal dysfunction':ti,ab,kw OR 'acute gastrointestinal injury':ti,ab,kw OR 'gastrointestinal diseases':ti,ab,kw	
#1	'bloodstream infection':ti,ab,kw OR 'bloodstream infections':ti,ab,kw OR 'infection, bloodstream':ti,ab,kw OR pyemia:ti,ab,kw OR pyemias:ti,ab,kw OR pyohemia:ti,ab,kw OR pyohemias:ti,ab,kw OR pyaemia:ti,ab,kw OR pyaemias:ti,ab,kw OR septicemia:ti,ab,kw OR septicemias:ti,ab,kw OR 'poisoning, blood':ti,ab,kw OR 'blood poisoning':ti,ab,kw OR 'blood poisonings':ti,ab,kw OR 'poisonings, blood':ti,ab,kw OR 'severe sepsis':ti,ab,kw OR 'sepsis, severe':ti,ab,kw OR sepsis:ti,ab,kw	228,213

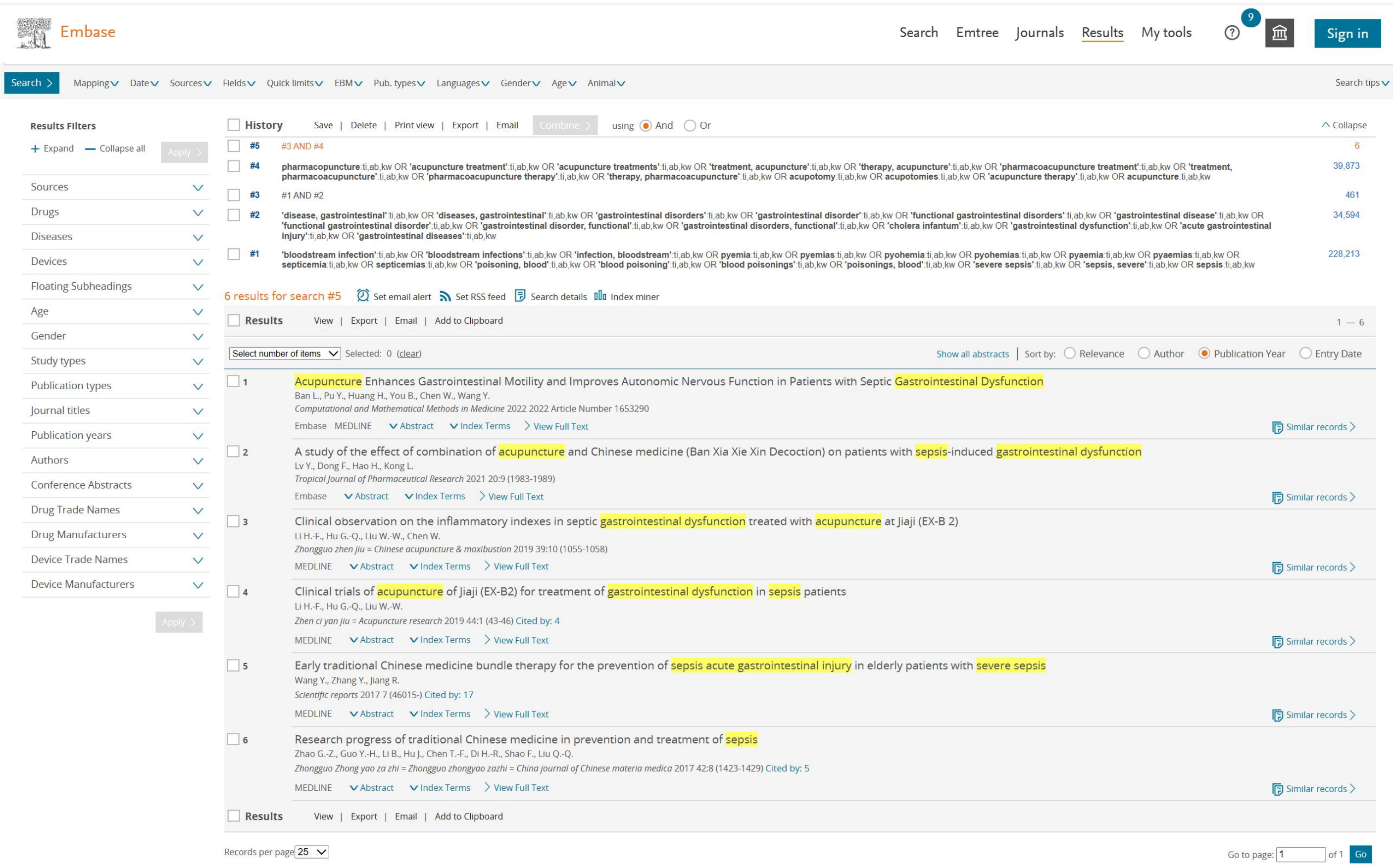

(1) Ban, L., et al. (2022). "Acupuncture Enhances Gastrointestinal Motility and Improves Autonomic Nervous Function in Patients with Septic Gastrointestinal Dysfunction." Comput Math Methods Med 2022.

(2) Li, H. F., et al. (2019). "Clinical trials of acupuncture of Jiaji (EX-B2) for treatment of gastrointestinal dysfunction in sepsis patients." Zhen Ci Yan Jiu 44(1): 43-46.

(3) Li, H. F., et al. (2019). "Clinical observation on the inflammatory indexes in septic gastrointestinal dysfunction treated with acupuncture at Jiaji (EX-B 2)." Zhongguo Zhen Jiu 39(10): 1055-1058.

(4) Lv, Y., et al. (2021). "A study of the effect of combination of acupuncture and Chinese medicine (Ban Xia Xie Xin Decoction) on patients with sepsis-induced gastrointestinal dysfunction." Tropical Journal of Pharmaceutical Research 20(9): 1983-1989.

(5) Wang, Y., et al. (2017). "Early traditional Chinese medicine bundle therapy for the prevention of sepsis acute gastrointestinal injury in elderly patients with severe sepsis." Scientific reports 7: 46015.

(6) Zhao, G. Z., et al. (2017). "Research progress of traditional Chinese medicine in prevention and treatment of sepsis." Zhongguo Zhong yao za zhi = Zhongguo zhongyao zazhi = China journal of Chinese materia medica 42(8): 1423-1429.

2.3 Cochrane Library (https://www.cochranelibrary.com/)

Search date:September 25, 2023 Beijing time

ID	Search	Hits
#1	(Bloodstream Infection OR Bloodstream Infections OR Infection, Bloodstream OR Pyemia OR Pyemias OR Pyohemia OR Pyohemias OR Pyaemia OR Pyaemias	7745

	OR Septicemia OR Septicemias OR Poisoning, Blood OR Blood Poisoning OR Blood Poisonings OR Poisonings, Blood OR Severe Sepsis OR Sepsis, Severe):ti,ab,kw	
#2	MeSH descriptor: [Sepsis] explode all trees	6810
#3	#1 OR #2	12539
#4	MeSH descriptor: [Gastrointestinal Diseases] explode all trees	46173
#5	(Disease, Gastrointestinal OR Diseases, Gastrointestinal OR Gastrointestinal Disease OR Gastrointestinal Disorders OR Gastrointestinal Disorder OR Functional Gastrointestinal Disorders OR Functional Gastrointestinal Disorder OR Gastrointestinal Disorder, Functional OR Gastrointestinal Disorders, Functional OR Cholera Infantum OR gastrointestinal dysfunction OR acute gastrointestinal injury):ti,ab,kw	26848
#6	#4 OR #5	66700
#7	MeSH descriptor: [Acupuncture] explode all trees	713
#8	MeSH descriptor: [Acupuncture Therapy] explode all trees	6467
#9	(Pharmacopuncture OR Acupuncture Treatment OR Acupuncture Treatments OR Treatment, Acupuncture OR Therapy, Acupuncture OR Pharmacoacupuncture	19153

	Treatment OR Treatment, Pharmacoacupuncture OR Pharmacoacupuncture Therapy OR Therapy, Pharmacoacupuncture OR Acupotomy OR Acupotomies OR Acupuncture Therapy OR acupuncture):ti,ab,kw	
#10	#7 OR #8 OR #9	19571
#11	#3 AND #6 AND #10	10

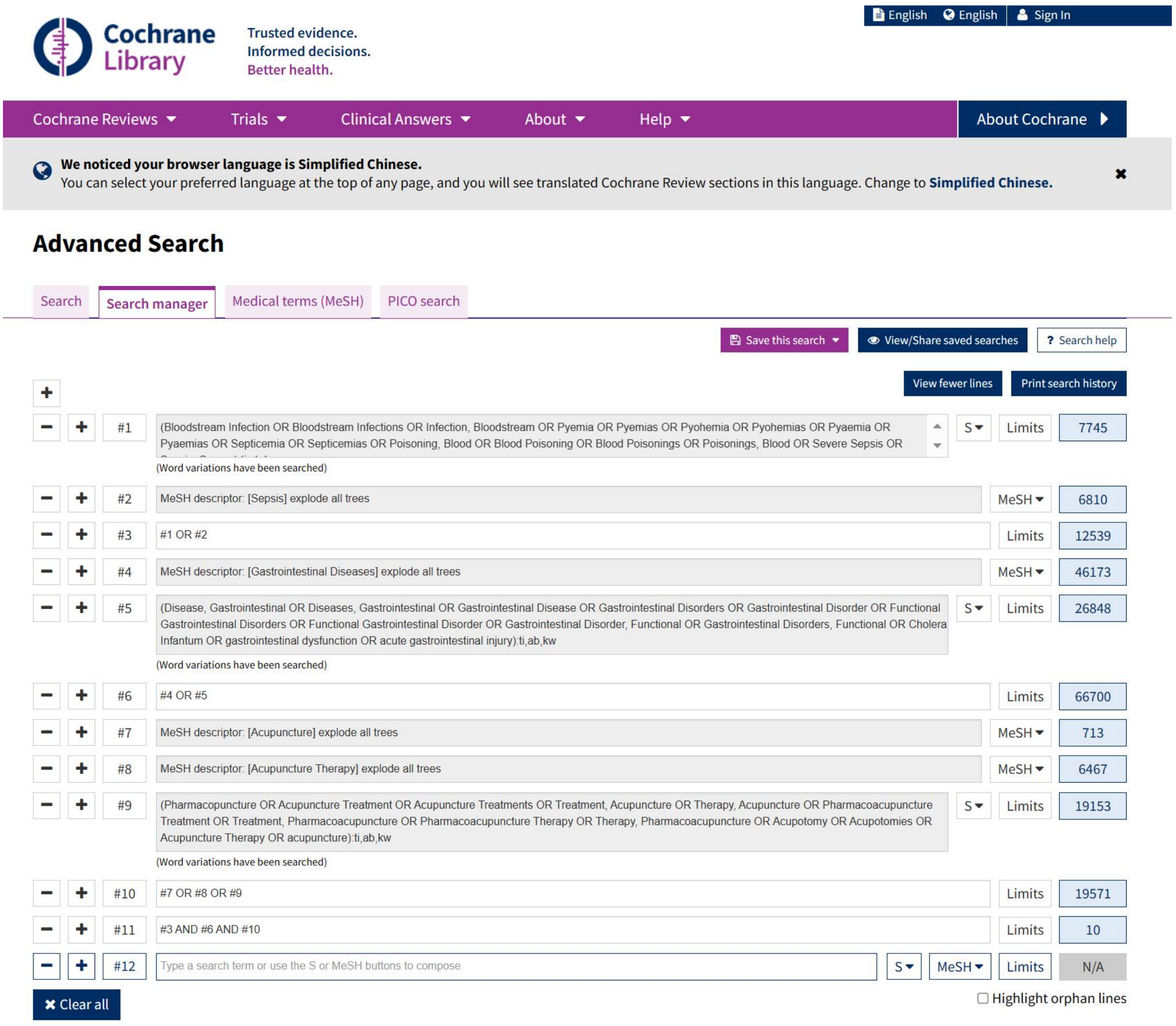

(1) HF Li, GQ Hu, WW Liu. Clinical trials of acupuncture of Jiaji (EX-B2) for treatment of gastrointestinal dysfunction in sepsis patients. Zhen CI yan jiu = acupuncture research, 2019, 44(1), 43 - 46.

(2) HF Li, GQ Hu, WW Liu, W Chen. Clinical observation on the inflammatory indexes in septic gastrointestinal dysfunction treated with acupuncture at Jiaji (EX-B 2). Zhongguo zhen jiu [Chinese acupuncture & moxibustion], 2019, 39(10), 1055 - 1058.
(3) L Ban, Y Pu, H Huang, B You, W Chen, Y Wang. Acupuncture Enhances Gastrointestinal Motility and Improves Autonomic Nervous Function in Patients with Septic Gastrointestinal Dysfunction. Computational and mathematical methods in medicine, 2022, 2022, 1653290.
(4) Q Fan, C Lei, Y Wang, N Yu, L Wang, J Fu, H Dong, Z Lu, L Xiong. Transcutaneous Electrical Acupoint Stimulation Combined With Auricular Acupressure Reduces Postoperative Delirium Among Elderly Patients Following Major Abdominal Surgery: a Randomized Clinical Trial. Frontiers in medicine, 2022, 9, 855296.
(5) H Liu, J Zhu, HB Ni, XX Hu. Transcutaneous electrical acupoint stimulation for early enteral nutrition tolerance in patients with sepsis of gastrointestinal dysfunction: a multi-center randomized controlled trial. Zhongguo zhen jiu [Chinese acupuncture & moxibustion], 2020, 40(3), 229 - 233.
(6) Y Lv, F Dong, H Hao, L Kong. A study of the effect of combination of acupuncture and Chinese medicine (Ban Xia Xie Xin Decoction) on patients with sepsis-induced gastrointestinal dysfunction. Tropical journal of pharmaceutical research, 2021, 20(9), 1983 - 1989.
(7) TD Martin, MS Green, MT Whitehead, TP Scheett, MJ Webster, GM Hudson. Six weeks of oral Echinacea purpurea supplementation does not enhance the production of serum erythropoietin or erythropoietic status in recreationally active males with above-average aerobic fitness. Applied physiology, nutrition & metabolism, 2019, 44(7), 791-795.
(8) The adaptive protection of acupuncture against gastrointestinal failure in patients with sepsis. ChiCTR-IOR-16009022. https://trialsearch.who.int/Trial2.aspx?TrialID=ChiCTR-IOR-16009022, 2016.
(9) IM Pacheco-Cerrato, P Cobos-Moreno, MA Castro-Avalos, J Fernandez-La-Villa, JF Moran-Cortes, JM Moran . Comment on "electroacupuncture Improves Intestinal Dysfunction in Septic Patients: a Randomised Controlled Trial" . BioMed research international, 2021, 2021, 6487272.
(10) B Meng, YN Jiao, G Zhang, XJ Xu, CL Ji, MH Hu, ZZ Lai, M Zhang. Electroacupuncture Improves Intestinal Dysfunction in Septic Patients: a Randomised Controlled Trial.JBioMed research international, 2018, 2018, 8293594.

2.4 CNKI (https://www.cnki.net/)

Search date:September 27, 2023 Beijing time

Search strategies: (TKA='急性胃肠损伤' OR TKA='胃肠功能障碍') AND (SU='脓毒症' OR TKA='脓毒血症' OR TKA='脓血症' OR TKA='败血病' OR TKA='败血症') AND (SU='针灸' OR TKA='针灸' OR TKA='针刺')

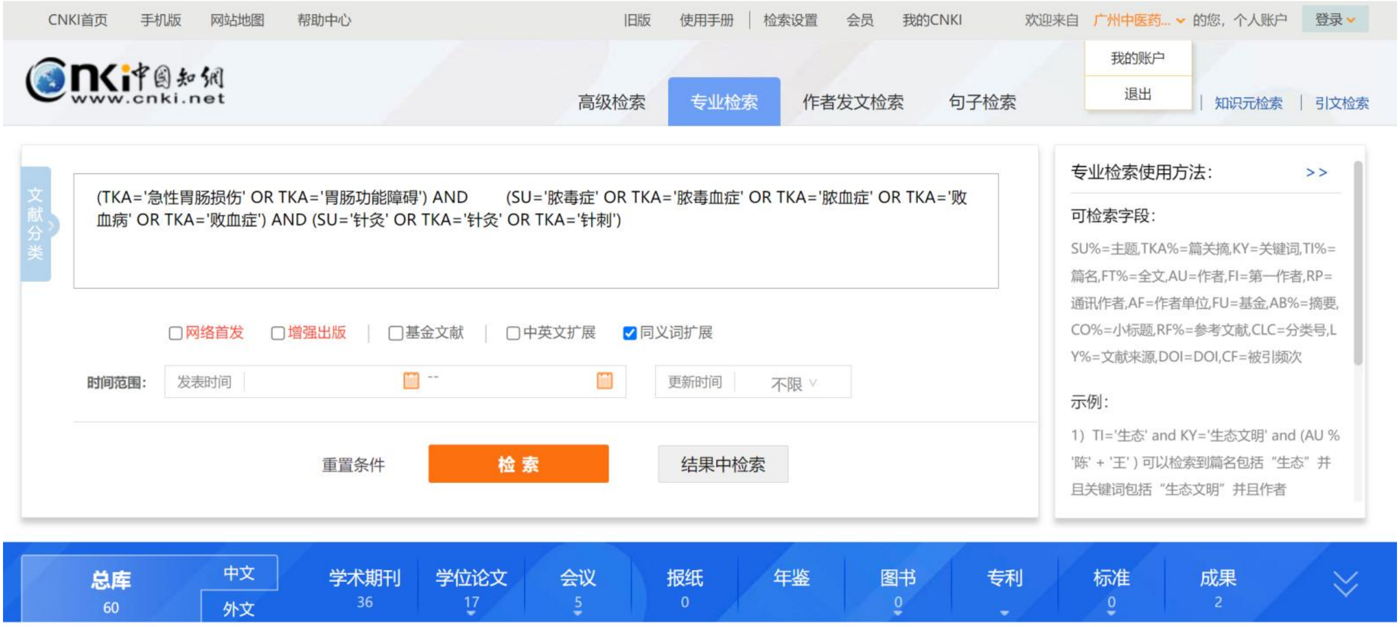

[1]葛旭,吴迪,王国兴等.生大黄灌肠联合针灸治疗脓毒症患者胃肠功能的临床观察[J].世界中西医结合杂志,2023,18(07):1391-1395+1400.DOI:10.13935/j.cnki.sjzx.230720.

[2]张笑言. 基于“肺与大肠相表里”理论针刺治疗脓毒症胃肠功能障碍的临床研究[D].黑龙江中医药大学,2023.DOI:10.27127/d.cnki.ghlzu.2023.000743.

[3]于佳琪,梁群,刘雨默等.中医药治疗脓毒症胃肠功能障碍的研究进展[J].中国中医急症,2023,32(03):545-547+551.

[4]袁红,徐培鑫,李航宇等.穴位注射治疗急性胰腺炎胃肠功能障碍的研究进展[J].中外医学研究,2022,20(36):165-168.DOI:10.14033/j.cnki.cfmr.2022.36.043.

[5]彭小菊,钟迪,何琪.早期肠内营养配合复元针法治疗脓毒症急性胃肠损伤的疗效观察[J].中国中西医结合消化杂志,2022,30(12):838-843.

[6]郭文辉,于秋香,孙玮婷等.中医外治法在 ICU 内的应用研究进展[J].世界科学技术-中医药现代化,2022,24(03):1278-1285.

[7]高江宝,罗伟君,李炜明等.基于肺与大肠相表里探讨早期防治胃肠功能障碍对重症肺炎的影响[J].江西中医药大学学报,2021,33(06):29-32.

[8]连佳明,钱义明,李璟等.益气健脾针法修复急性胃肠损伤肠道屏障的临床研究[J].中国中医急症,2021,30(12):2119-2122.

[9]陈周燕,闫如玉,杨晋翔.脓毒症胃肠功能障碍的研究进展[J].实用中医内科杂志,2021,35(11):108-111.DOI:10.13729/j.issn.1671-7813.Z20201302.

[10]王婷玉,孟捷.针灸辅助治疗脓毒症胃肠功能障碍疗效 Meta 分析[J].辽宁中医药大学学报,2022,24(02):165-170.DOI:10.13194/j.issn.1673-842x.2022.02.037.

[11]程璐. 针刺治疗脓毒症急性胃肠损伤的随机对照研究[D].北京中医药大学,2022.DOI:10.26973/d.cnki.gbjzu.2021.000715.

[12]陈嘉琪,钱义明,钱风华等.基于脑肠肽探讨针刺治疗急性胃肠损伤的作用机制[J].实用临床医药杂志,2021,25(18):120-123.

[13]连佳明,钱义明,钱风华等.针刺治疗脓毒症急性胃肠损伤的研究进展[J].中医药导报,2021,27(07):148-151.DOI:10.13862/j.cnki.cn43-1446/r.2021.07.027.

[14] 王柳，李淑芳．中医药治疗脓毒症胃肠功能障碍研究进展[J].新中医,2021,53(13):1-4.DOI:10.13457/j.cnki.jncm.2021.13.001.
[15] 常乐．脓毒症胃肠功能障碍证型分布及针刺疗效研究[D].天津中医药大学,2022.DOI:10.27368/d.cnki.gtzyy.2021.000307.
[16]李亚莉,褚玉茹,于乃浩等.针灸改善脓毒症患者胃肠功能障碍的临床观察[J].天津中医药,2021,38(04):479-482.
[17]孙一凡. 基于气机升降理论运用电针干预脓毒症患者急性胃肠损伤的临床研究[D].南京中医药大学,2022.DOI:10.27253/d.cnki.gnjzu.2021.000220.
[18] 刘欢． 电针对重症患者胃肠功能障碍的疗效观察[D].南京中医药大学,2022.DOI:10.27253/d.cnki.gnjzu.2021.000724.
[19]陈红,欧阳红莲,潘卫红等.中医外治法在脓毒症胃肠功能障碍中的应用进展[J].当代临床医刊,2021,34(01):85-86+58.
[20]孙一凡,戴林峰,袁思成等.针刺治疗脓毒症胃肠功能障碍临床研究进展[J].中国中医药图书情报杂志,2020,44(06):68-70.
[21]胡国强, 脊神经定位结合针刺华佗夹脊穴取穴治疗脓毒血症胃肠功能障碍临床疗效研究. 浙江省,长兴县中医院,2020-10-10.
[22]徐畅,梁建峰,李智勇等.老年脓毒症胃肠功能障碍的中医证素分布及其针刺疗效观察[J].世界中医药,2020,15(16):2499-2502.
[23]王晶. 针刺联合大承气汤对脓毒症毒热内盛证肠道菌群的影响[D].山东中医药大学,2022.DOI:10.27282/d.cnki.gsdzu.2020.000320.
[24]赵国桢. 基于中医药治疗脓毒症的临床实践指南制定及其再评价研究[D].北京中医药大学,2021.DOI:10.26973/d.cnki.gbjzu.2020.000911.
[25]徐武兵,韩春林,泽让吉波等.中医外治法治疗脓毒症胃肠功能损伤进展[J].基层医学论坛,2020,24(11):1591-1593.DOI:10.19435/j.1672-1721.2020.11.073.
[26]刘超,肖阳春,梁立新等.针刺联合多潘立酮治疗脓毒症急性胃肠损伤疗效观察[J].上海针灸杂志,2020,39(01):6-10.DOI:10.13460/j.issn.1005-0957.2020.01.0006.
[27]张俭,黄竞,常卫东等. 针刺疗法治疗脓毒症临床疗效和安全性的 Meta 分析[C]//世界中医药学会联合会老年医学专业委员会,中国中西医结合学会慢病防治与管理专业委员会.世界中医药学会联合会老年医学专业委员会、中国中西医结合学会慢病防治与管理专业委员会 2019 学术年会论文摘要集.世界中医药学会联合会老年医学专业委员会,2019:1.DOI:10.26914/c.cnkihy.2019.073587.
[28]孙德阳,杨洋,杜纯鹏等.电针治疗脓毒症胃肠功能障碍的临床研究[J].针灸临床杂志,2019,35(10):47-50.
[29]李海峰,胡国强,刘雯雯等.针刺夹脊穴对脓毒症胃肠功能障碍炎性反应指标的影响[J].中国针灸,2019,39(10):1055-1058.DOI:10.13703/j.0255-2930.2019.10.006.
[30]刘凯，孙宏，李冀等．腹部电针对脓毒症胃肠功能障碍的影响[J].上海针灸杂志,2019,38(10):1118-1121.DOI:10.13460/j.issn.1005-0957.2019.10.1118.
[31]周添奕,钱风华,黄馨云等.基于数据挖掘探究危重症患者急性胃肠损伤的中医针灸诊治规律[J].世界科学技术-中医药现代化,2019,21(08):1606-1614.
[32]陈威,李海峰,胡国强.针刺夹脊穴对脓毒血症腹腔内压、胃腔残留量疗效评估[J].上海针灸杂志,2019,38(06):601-606.DOI:10.13460/j.issn.1005-0957.2019.06.0601.
[33]肖秋生.中药灌肠方+针刺联合常规疗法治疗 ICU 脓毒症胃肠功能障碍随机平行对照研究[J].实用中医内科杂志,2019,33(05):74-77.DOI:10.13729/j.issn.1671-7813.z20190026.
[34]张俭，黄竞，常卫东等．中医综合疗法治疗脓毒症胃肠功能障碍临床研究[J].新中医,2019,51(06):154-158.DOI:10.13457/j.cnki.jncm.2019.06.046.
[35]王晓鹏. 针刺辅助治疗脓毒症胃肠功能障碍的随机对照临床研究[D].北京中医药大学,2019.
[36]袁金霞,赖芳,杜炯栋等.运用晁恩祥教授经验针药结合防治脓毒症胃肠功能障碍的临床探讨[J].中国中医急症,2019,28(04):664-667.

[37]袁金霞. 针药结合法对脓毒症急性呼吸窘迫综合症患者胃肠功能影响的临床研究[D].广州中医药大学,2022.DOI:10.27044/d.cnki.ggzzu.2019.001287.
[38]王丽娟. 腹针治疗脓毒症急性胃肠损伤的临床研究[D].广州中医药大学,2020.DOI:10.27044/d.cnki.ggzzu.2019.000892.
[39]周建明,廖吕钊,王希等.脓毒症急性胃肠损伤下胃肠激素胃动素和Ghrelin变化及针刺对其影响的研究进展[J].全科医学临床与教育,2019,17(03):252-254.DOI:10.13558/j.cnki.issn1672-3686.2019.03.019.
[40]李海峰,胡国强,刘雯雯.针刺华佗夹脊穴治疗脓毒血症胃肠功能障碍临床疗效观察[J].针刺研究,2019,44(01):43-46.DOI:10.13702/j.1000-0607.170579.
[41]王丽娟,李健,李小娟.针刺疗法治疗脓毒症临床疗效和安全性的Meta分析[J].中医药导报,2018,24(23):86-90.DOI:10.13862/j.cnki.cn43-1446/r.2018.23.027.
[42]王丽娟,李健. 针刺疗法用于治疗脓毒症的随机对照试验的Meta分析[C]//中国中西医结合学会慢病防治与管理专业筹备委员会,世界中医药学会联合会老年医学专业委员会.中国中西医结合学会慢病防治与管理专业委员会成立大会暨世界中医药学会联合会老年医学专业委员会第七届学术年会——新时代·推进老年慢病防治与管理论文集.中国中西医结合学会慢病防治与管理专业委员会成立大会暨世界中医药学会联合会老年医学专业委员会第七届学术年会——新时代·推进老年慢病防治与管理论文集,2018:1.DOI:10.26914/c.cnkihy.2018.012083.
[43]张俭,黄竞,常卫东等. 中医综合疗法对脓毒症胃肠功能障碍干预的临床研究[C]//中国中西医结合学会慢病防治与管理专业筹备委员会,世界中医药学会联合会老年医学专业委员会.中国中西医结合学会慢病防治与管理专业委员会成立大会暨世界中医药学会联合会老年医学专业委员会第七届学术年会——新时代·推进老年慢病防治与管理论文集.中国中西医结合学会慢病防治与管理专业委员会成立大会暨世界中医药学会联合会老年医学专业委员会第七届学术年会——新时代·推进老年慢病防治与管理论文集,2018:1.DOI:10.26914/c.cnkihy.2018.012084.
[44]张艳秋,马柯,董振飞.脓毒症胃肠功能损伤中医外治法近况[J].时珍国医国药,2018,29(06):1438-1439.
[45]刘丹蕾. 非药物疗法对机械通气患者急性胃肠损伤的影响[D].南方医科大学,2019.
[46]黄文婷. 穴位艾灸治疗脓毒症胃肠功能损伤的临床观察[D].北京中医药大学,2018.
[47]丁杰.中医药治疗脓毒症胃肠功能障碍概况[J].中外女性健康研究,2017(17):4-5+16.
[48]刘清泉,陈仁波,李博. 中医药补充抗生素治疗脓毒症的专家共识[C]//中国科学技术协会,吉林省人民政府.第十九届中国科协年会——分12标准引领中医药学术创新发展高峰论坛论文集.第十九届中国科协年会——分12标准引领中医药学术创新发展高峰论坛论文集,2017:1.
[49]常卫东. 中医综合疗法治疗脓毒症胃肠功能障碍的临床研究[D].广州中医药大学,2018.
[50]韦志友. 复元针法治疗脓毒症患者急性胃肠损伤的临床观察[D].北京中医药大学,2017.
[51]赵国桢,郭玉红,李博等.中医药防治脓毒症的研究进展[J].中国中药杂志,2017,42(08):1423-1429.DOI:10.19540/j.cnki.cjcmm.2017.0038.
[52]于文琦. 艾灸足三里穴缓解卒中后疲劳的临床研究[D].广州中医药大学,2018.
[53]陈分乔, 中医集束化治疗防治脓毒症肠功能障碍的临床研究. 河北省,河北省中医院,2016-12-22.
[54]王益斐. 中医药集束化措施对预防严重脓毒症患者急性胃肠损伤的临床研究[D].浙江中医药大学,2022.DOI:10.27465/d.cnki.gzzyc.2016.000207.
[55]黄展明. 宣肺利气法针刺对胃肠癌术后胃肠功能恢复的临床观察[D].广州中医药大学,2017.
[56]张利娟,张广清,吴巧媚等.脓毒症肠功能障碍的中医外治法研究进展[J].江西中医药,2015,46(11):67-69.
[57]王益斐,智屹惠,江荣林等. 早期中医药集束化措施对预防脓毒症急性胃肠损伤的临床观察[C]//中华医学会,中华医学会重症医学分会.中华医学会第二届重症心脏全国学术大会暨第三届西湖重症医学论坛、2015年浙江省重症医学学术年会论文汇编.中华医学会第二届重症心脏全国学术大会暨第三届西湖重症医学论坛,2015:10.

[58]虞意华,金肖青,俞迈红等.针灸对老年严重脓毒症患者胃肠功能及胃肠激素影响的临床研究[J].中华中医药学刊,2015,33(08):1953-1956.DOI:10.13193/j.issn.1673-7717.2015.08.048.
[59]蔡莉娟,丁学军,刘文兵等.电针对脓毒症患者胃肠功能障碍的干预作用[J].中国中医急症,2014,23(02):268-270.
[60]王晶晶,孔祥照,张晓璇.针刺足三里对脓毒症胃肠功能障碍患者腹内压的影响[J].中国中医急症,2012,21(11):1834-1835.

2.5 Wanfang data(https://www.wanfangdata.com.cn/)

Search date:September 26, 2023 Beijing time

Search strategies:（主题:(针灸) or 题名或关键词:(针灸) or 摘要:(针灸) or 题名或关键词:(针刺) or 关键词:(针刺)) and (主题:(脓毒症) or 题名或关键词:(脓毒血症) or 关键词:(脓毒血症) or 题名或关键词:(脓血症) or 摘要:(脓血症) or 题名或关键词:(败血病) or 关键词:(败血病) or 题名或关键词:(败血症) or 关键词:(败血症)) and （ 题名或关键词:(急性胃肠损伤) or 关键词:(急性胃肠损伤) or 题名或关键词:(胃肠功能障碍) or 关键词:(胃肠功能衰竭) ）

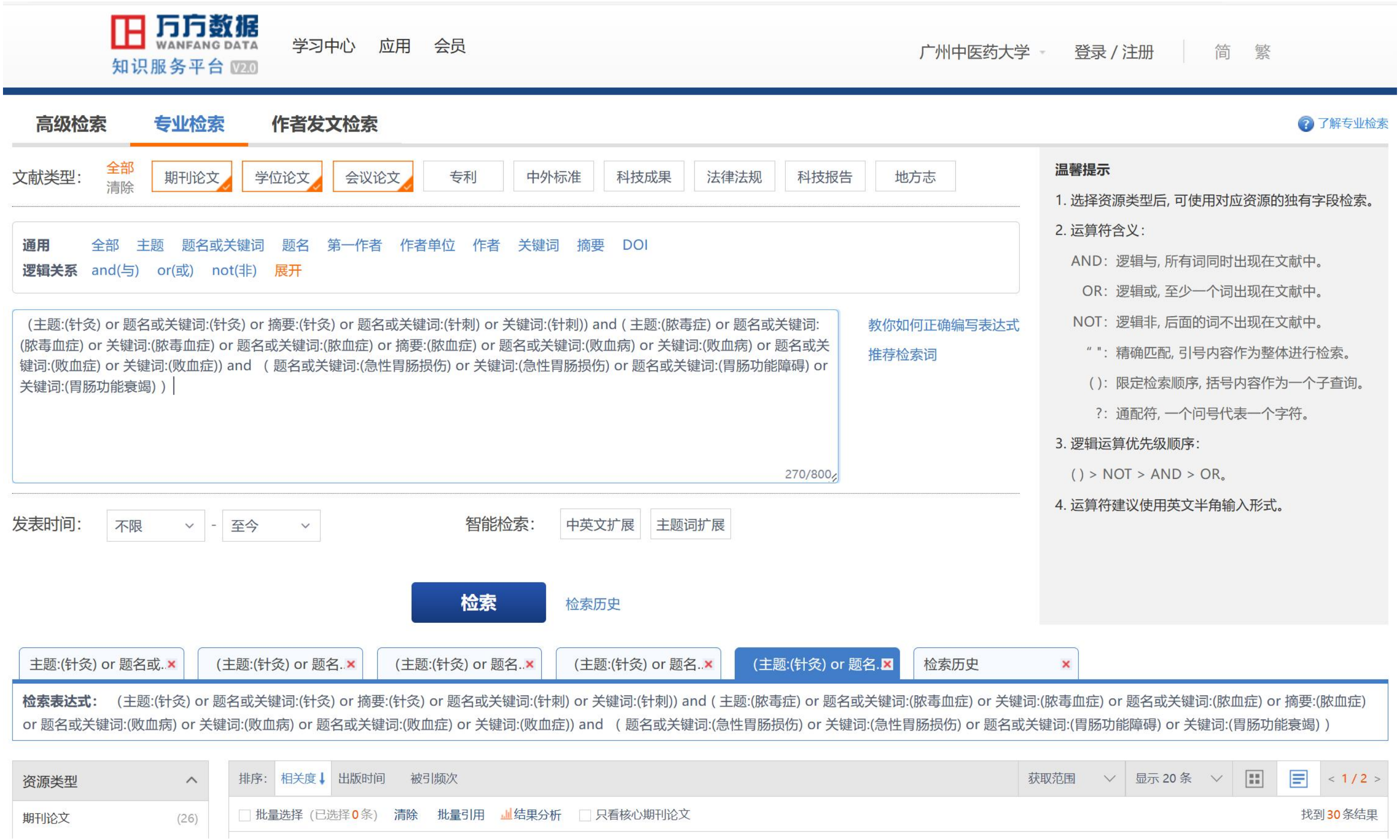

[1] 李亚莉,褚玉茹,于乃浩,等. 针灸改善脓毒症患者胃肠功能障碍的临床观察[J]. 天津中医药,2021,38(4):479-482. DOI:10.11656/j.issn.1672-1519.2021.04.15.
[2] 王婷玉,孟捷. 针灸辅助治疗脓毒症胃肠功能障碍疗效 Meta 分析[J]. 辽宁中医药大学学报,2022,24(2):165-170. DOI:10.13194/j.issn.1673-842x.2022.02.037.
[3] 刘超,肖阳春,梁立新,等. 针刺联合多潘立酮治疗脓毒症急性胃肠损伤疗效观察[J]. 上海针灸杂志,2020,39(1):6-10. DOI:10.13460/j.issn.1005-0957.2020.01.0006.
[4] 于佳琪,梁群,刘雨默,等. 中医药治疗脓毒症胃肠功能障碍的研究进展[J]. 中国中医急症,2023,32(3):545-547,551. DOI:10.3969/j.issn.1004-745X.2023.03.044.
[5] 孙一凡,戴林峰,袁思成,等. 针刺治疗脓毒症胃肠功能障碍临床研究进展[J]. 中国中医药图书情报杂志,2020,44(6):68-70. DOI:10.3969/j.issn.2095-5707.2020.06.017.
[6] 陈嘉琪,钱义明,钱风华,等. 基于脑肠肽探讨针刺治疗急性胃肠损伤的作用机制[J]. 实用临床医药杂志,2021,25(18):120-123. DOI:10.7619/jcmp.20211167.
[7] 田锴熙,刘毅. 针刺疗法治疗脓毒症胃肠功能障碍的研究进展[J]. 世界最新医学信息文摘（连

续型电子期刊）,2023,23(53):91-97. DOI:10.3969/j.issn.1671-3141.2023.053.016.
[8] 肖秋生. 中药灌肠方+针刺联合常规疗法治疗 ICU 脓毒症胃肠功能障碍随机平行对照研究[J]. 实用中医内科杂志,2019,33(5):74-76,封 3. DOI:10.13729/j.issn.1671-7813.Z20190026.
[9] 连佳明,钱义明,钱风华,等. 针刺治疗脓毒症急性胃肠损伤的研究进展[J]. 中医药导报,2021,27(7):148-151.
[10] 王晶晶,孔祥照,张晓璇. 针刺足三里对脓毒症胃肠功能障碍患者腹内压的影响[J]. 中国中医急症,2012,21(11):1834-1835. DOI:10.3969/j.issn.1004-745X.2012.11.065.
[11] 周建明,廖吕钊,王希,等. 脓毒症急性胃肠损伤下胃肠激素胃动素和 Ghrelin 变化及针刺对其影响的研究进展[J]. 全科医学临床与教育,2019,17(3):252-254. DOI:10.13558/j.cnki.issn1672-3686.2019.03.019.
[12] 李海峰,胡国强,刘雯雯. 针刺华佗夹脊穴治疗脓毒血症胃肠功能障碍临床疗效观察[J]. 针刺研究,2019,44(1):43-46. DOI:10.13702/j.1000-0607.170579.
[13] 徐畅,梁建峰,李智勇,等. 老年脓毒症胃肠功能障碍的中医证素分布 及其针刺疗效观察[J]. 世界中医药,2020,15(16):2499-2502. DOI:10.3969/j.issn.1673-7202.2020.16.032.
[14] 方军,李兰,李冰冰,等. 针灸对严重脓毒症患者胃肠功能障碍及胃肠激素影响分析[J]. 湖南中医药大学学报,2016,36(0):11.
[15] 王玉玉. 中医特色疗法治疗脓毒症胃肠功能障碍的研究进展[J]. 医药前沿,2017,7(32):6-8. DOI:10.3969/j.issn.2095-1752.2017.32.003.
[16] 许永豪,罗艳霞,陈巧. 大承气汤保留灌肠对脓毒症胃肠功能障碍患者炎症反应的影响[J]. 中西医结合研究,2021,13(6):398-401. DOI:10.3969/j.issn.1674-4616.2021.06.010.
[17] 陈红,欧阳红莲,潘卫红,等. 中医外治法在脓毒症胃肠功能障碍中的应用进展[J]. 当代临床医刊,2021,34(1):85-86,58. DOI:10.3969/j.issn.2095-9559.2021.01.056.
[18] 魏英凯. 针刺联合神阙穴贴敷治疗脓毒症胃肠功能障碍疗效观察[J]. 健康必读,2021(34):86.
[19] 连佳明,钱义明,李璟,等. 益气健脾针法修复急性胃肠损伤肠道屏障的临床研究[J]. 中国中医急症,2021,30(12):2119-2122. DOI:10.3969/j.issn.1004-745X.2021.12.012.
[20] 刘凯,孙宏,李冀,等. 腹部电针对脓毒症胃肠功能障碍的影响[J]. 上海针灸杂志,2019,38(10):1118-1121. DOI:10.13460/j.issn.1005-0957.2019.10.1118.
[21] 李海峰,胡国强,刘雯雯,等. 针刺夹脊穴对脓毒症胃肠功能障碍炎性反应指标的影响[J]. 中国针灸,2019,39(10):1055-1058. DOI:10.13703/j.0255-2930.2019.10.006.
[22] 高江宝,罗伟君,李炜明,等. 基于肺与大肠相表里探讨早期防治胃肠功能障碍对重症肺炎的影响[J]. 江西中医药大学学报,2021,33(6):29-32.
[23] 杨卉卉. 针刺治疗脓毒症患者脾胃虚弱型胃肠功能障碍的临床研究[D]. 山东:山东中医药大学,2018.
[24] 蔡莉娟,丁学军,刘文兵,等. 电针对脓毒症患者胃肠功能障碍的干预作用[J]. 中国中医急症,2014,23(2):268-270. DOI:10.3969/j.issn.1004-745X.2014.02.034.
[25] 杨金亮,齐文升,杨秀捷,等. 通调平衡针刺法治疗脓毒症胃肠功能障碍的理论研究[C]. //世界中医药学会联合会医院感染管理专业委员会第二届学术年会论文集. 2017:7-11.
[26] 丁杰. 中医药治疗脓毒症胃肠功能障碍概况[J]. 中外女性健康研究,2017(17):4-5,16. DOI:10.3969/j.issn.2096-0417.2017.17.003.
[27] 陈威,李海峰,胡国强. 针刺夹脊穴对脓毒血症腹腔内压、胃腔残留量疗效评估[J]. 上海针灸杂志,2019,38(6):601-606.
[28] 刘丹蕾. 非药物疗法对机械通气患者急性胃肠损伤的影响[D]. 广东:南方医科大学,2018. DOI:10.7666/d.Y3475187.
[29] 王益斐,智屹惠,江荣林,等. 早期中医药集束化措施对预防脓毒症急性胃肠损伤的临床观察[C]. //中华医学会第 10 次全国重症医学大会论文集. 2016:349-349.
[30] 张艳秋,马柯,董振飞. 脓毒症胃肠功能损伤中医外治法近况[J]. 时珍国医国

药,2018,29(6):1438-1439. DOI:10.3969/j.issn.1008-0805.2018.06.058.

2.6 Cqvip（http://www.cqvip.com/）

Search date:September 27, 2023 Beijing time

Search strategies: (M= (针灸 OR 针刺) OR R= (针灸 OR 针刺)) AND (M= (脓毒症 OR 脓毒血症 OR 脓血症 OR 败血病 OR 败血症) OR R= (脓毒症 OR 脓毒血症 OR 脓血症 OR 败血病 OR 败血症)) AND (M= (急性胃肠损伤 OR 胃肠功能障碍) OR R= (急性胃肠损伤 OR 胃肠功能障碍))

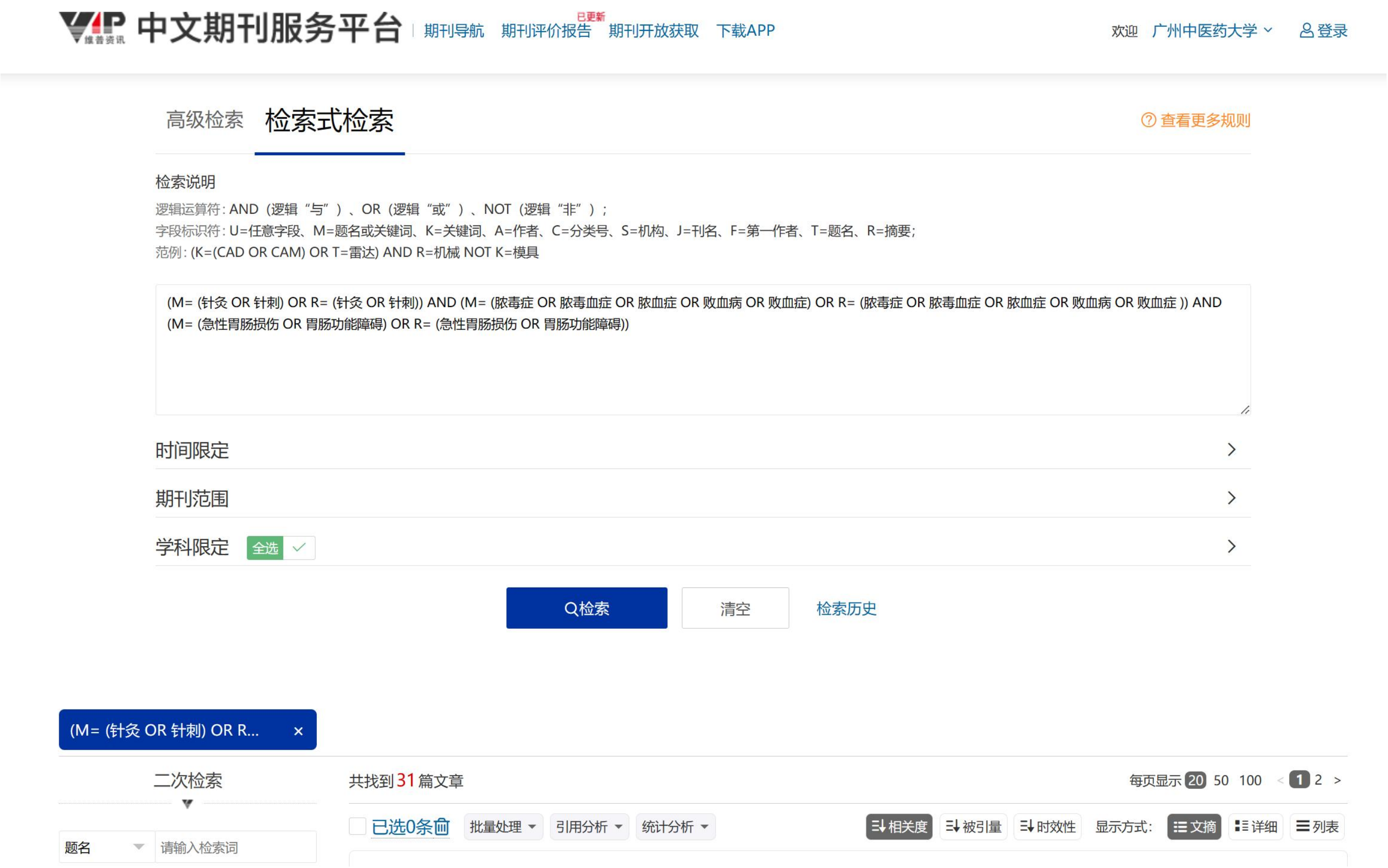

[1]王晶晶,孔祥照,张晓璇.针刺足三里对脓毒症胃肠功能障碍患者腹内压的影响[J].中国中医急症,2012,21(11):1834-1835.
[2]方军,李兰,李冰冰,赵娟,朱岩,王海云,池丽芳.针灸对严重脓毒症患者胃肠功能障碍及胃肠激素影响分析[J].湖南中医药大学学报,2016,36(A01):11-11.
[3]李亚莉,褚玉茹,于乃浩,阚建英.针灸改善脓毒症患者胃肠功能障碍的临床观察[J].天津中医药,2021,38(4):479-482.
[4]孙一凡,戴林峰,袁思成,王醒.针刺治疗脓毒症胃肠功能障碍临床研究进展[J].中国中医药图书情报杂志,2020,44(6):68-70.
[5]张小桐,蔡卫敏,刘书华.1 例脓毒症休克合并胃肠功能障碍患者的中医护理体会[J].中西医结合护理,2023,9(3):25-28.
[6]徐畅,梁建峰,李智勇,董海山,尹超群.老年脓毒症胃肠功能障碍的中医证素分布及其针刺疗效观察[J].世界中医药,2020,15(16):2499-2502.
[7]刘超,肖阳春,梁立新,郭玉红.针刺联合多潘立酮治疗脓毒症急性胃肠损伤疗效观察[J].上海针灸杂志,2020,39(1):6-10.
[8]连佳明,钱义明,钱风华,李璟,陈嘉琪,陈晓桐,丁纯蕾.针刺治疗脓毒症急性胃肠损伤的研究进展[J].中医药导报,2021,27(7):148-151.
[9]于佳琪,梁群,刘雨默,刘洋,李鹤然.中医药治疗脓毒症胃肠功能障碍的研究进展[J].中国中医急

症,2023,32(3):545-547+551.
[10]周建明,廖吕钊,王希,李雯静,潘思旭,徐娜菲,江荣林.脓毒症急性胃肠损伤下胃肠激素胃动素和Ghrelin 变化及针刺对其影响的研究进展[J].全科医学临床与教育,2019,17(3):252-254.
[11]刘凯,孙宏,李冀,尚莉莉,全爱君,蔡国锋.腹部电针对脓毒症胃肠功能障碍的影响[J].上海针灸杂志,2019,38(10):1118-1121.
[12]王婷玉,孟捷.针灸辅助治疗脓毒症胃肠功能障碍疗效 Meta 分析[J].辽宁中医药大学学报,2022,24(2):165-170.
[13]许永豪,罗艳霞,陈巧.大承气汤保留灌肠对脓毒症胃肠功能障碍患者炎症反应的影响[J].中西医结合研究,2021,13(6):398-401.
[14]蔡莉娟,丁学军,刘文兵,张艳,周三军,胡丹丹.电针对脓毒症患者胃肠功能障碍的干预作用[J].中国中医急症,2014,23(2):268-270.
[15]陈红,欧阳红莲,潘卫红,张广清.中医外治法在脓毒症胃肠功能障碍中的应用进展[J].当代临床医刊,2021,34(1):85-86+58.
[16]丁杰.中医药治疗脓毒症胃肠功能障碍概况[J].中外女性健康研究,2017(17):4-5+16.
[17]陈威,李海峰,胡国强.针刺夹脊穴对脓毒血症腹腔内压、胃腔残留量疗效评估[J].上海针灸杂志,2019,38(6):601-606.
[18]李海峰,胡国强,刘雯雯.针刺华佗夹脊穴治疗脓毒血症胃肠功能障碍临床疗效观察[J].针刺研究,2019,44(1):43-46.
[19]肖秋生.中药灌肠方+针刺联合常规疗法治疗 ICU 脓毒症胃肠功能障碍随机平行对照研究[J].实用中医内科杂志,2019,33(5):74-77.
[20]李海峰,胡国强,刘雯雯,陈威.针刺夹脊穴对脓毒症胃肠功能障碍炎性反应指标的影响[J].中国针灸,2019,39(10):1055-1058.
[21]陈嘉琪,钱义明,钱风华,李璟,连佳明,陈晓桐,林柏柏,丁纯蕾.基于脑肠肽探讨针刺治疗急性胃肠损伤的作用机制[J].实用临床医药杂志,2021,25(18):120-123.
[22]李莉,晏军,吴彩军,马林沁,郑广明,果海凤,王健,杨喆,牛丽强.针灸联合穴位贴敷法治疗急诊老年脓毒症机械通气患者继发肠功能障碍的疗效[J].中国医师杂志,2022,24(4):486-489.
[23]虞意华,金肖青,俞迈红,龚仕金,刘秉宇,李莉.针灸对老年严重脓毒症患者胃肠功能及胃肠激素影响的临床研究[J].中华中医药学刊,2015,33(8):1953-1956.
[24]高江宝,罗伟君,李炜明,史润霞,张用华.基于肺与大肠相表里探讨早期防治胃肠功能障碍对重症肺炎的影响[J].江西中医药大学学报,2021,33(6):29-32.
[25]张俭,黄竞,常卫东,张晓忠,张军,杨广,李健.中医综合疗法治疗脓毒症胃肠功能障碍临床研究[J].新中医,2019(6):154-158.
[26]王柳,李淑芳.中医药治疗脓毒症胃肠功能障碍研究进展[J].新中医,2021,53(13):1-4.
[27]张利娟,张广清,吴巧媚,彭鹿.脓毒症肠功能障碍的中医外治法研究进展[J].江西中医药,2015,46(11):67-69.
[28]连佳明,钱义明,李璟,钱风华,陈嘉琪,陈晓桐,丁纯蕾.益气健脾针法修复急性胃肠损伤肠道屏障的临床研究[J].中国中医急症,2021,30(12):2119-2122.
[29]张艳秋,马柯,董振飞.脓毒症胃肠功能损伤中医外治法近况[J].时珍国医国药,2018,29(6):1438-1439.
[30]彭晓洪,宋棠,黄永莲,黄壑霏,王评.针药结合疗法对脓毒症患者肠道屏障功能保护作用的临床研究[J].中国中西医结合急救杂志,2023,30(2):142-146.
[31]赵国桢,郭玉红,李博,胡晶,陈腾飞,狄浩然,邵飞,刘清泉.中医药防治脓毒症的研究进展[J].中国中药杂志,2017,42(8):1423-1429.

2.7 Chinese biomedical literature service system (Sinomed, http://www.sinomed.ac.cn/)

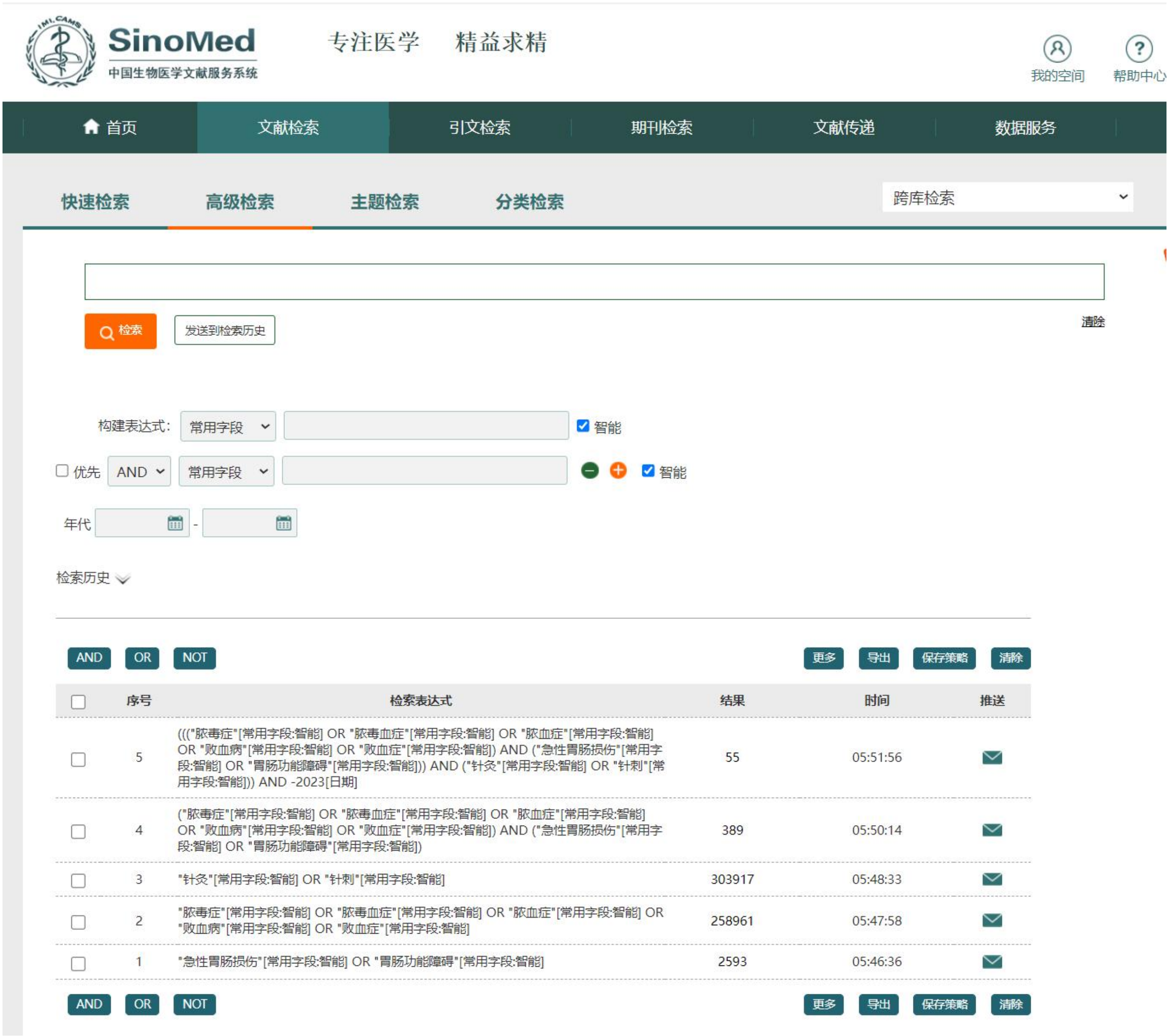

[1] 张萍萍,周逸丹,葛婷爱,等.四逆汤穴位贴敷恢复脓毒症休克患者胃肠功能的临床研究[J].辽宁中医杂志,2023,50(6):192-194.
[2] 葛旭,吴迪,王国兴,等.生大黄灌肠联合针灸治疗脓毒症患者胃肠功能的临床观察[J].世界中西医结合杂志,2023,18(7):1391-1395,1400.
[3] 彭晓洪,宋棠,黄永莲,等.针药结合疗法对脓毒症患者肠道屏障功能保护作用的临床研究[J].中国中西医结合急救杂志,2023,30(2):142-146.
[4] 牛丽强,杨喆,田丽,等.吴茱萸热敷联合耳穴压丸辅助治疗脓毒症胃肠功能障碍效果观察[J].北京中医药,2023,42(2):214-217.
[5] 陈文,曾奕云,何晓铭.温灸刮痧干预脓毒症胃肠功能障碍的疗效观察[J].中医外治杂志,2022,31(6):33-35.
[6] 伍万,江荣林,赵利娟,等.电针联合整肠生胶囊对胃肠功能障碍脓毒症患者早期肠内营养耐受性的影响[J].浙江临床医学,2022,24(11):1590-1592.
[7] 彭小菊,钟迪,何琪.早期肠内营养配合复元针法治疗脓毒症急性胃肠损伤的疗效观察[J].中国中西医结合消化杂志,2022,30(12):838-843.
[8] 叶颖颖,郭黄容,温正旺.中药穴位超声导入疗法治疗重症肺炎合并胃肠功能障碍的临床观察[J].中国中医药科技,2023,30(1):123-125.
[9] 袁红,徐培鑫,李航宇,等.穴位注射治疗急性胰腺炎胃肠功能障碍的研究进展[J].中外医学研

究,2022,20(36):165-168.
[10] 张萍萍,周逸丹,葛婷爱,等.四逆汤穴位贴敷联合双歧杆菌三联活菌散治疗脓毒症胃肠功能障碍临床研究[J].新中医,2022,54(21):43-46.
[11] 于佳琪,梁群,刘雨默,等.中医药治疗脓毒症胃肠功能障碍的研究进展[J].中国中医急症,2023,32(3):545-547,551.
[12] 杨洁,陈微,陈美华,等.整肠散穴位贴敷对脓毒症患者胃肠功能的影响[J].蛇志,2022,34(3):350-353.
[13] 王灿,高媛媛,刘春霞,等.中药膏摩联合穴位注射治疗脓毒症胃肠功能障碍疗效观察[J].现代中西医结合杂志,2022,31(14):1965-1968,1972.
[14] 周波巧,葛婷爱,冯晓菲.四逆汤穴位贴敷治疗脓毒症胃肠功能障碍 35 例[J].浙江中医杂志,2022,57(6):425,461.
[15] 林浩嘉,林海龙.电针联合隔药灸脐辅治脓毒症胃肠功能障碍的临床观察[J].中国中医急症,2022,31(4):666-669.
[16] 王婷玉,孟捷.针灸辅助治疗脓毒症胃肠功能障碍疗效 Meta 分析[J].辽宁中医药大学学报,2022,24(2):165-170.
[17] 连佳明,钱义明,李璟,等.益气健脾针法修复急性胃肠损伤肠道屏障的临床研究[J].中国中医急症,2021,30(12):2119-2122.
[18] 高江宝,罗伟君,李炜明,等.基于肺与大肠相表里探讨早期防治胃肠功能障碍对重症肺炎的影响[J].江西中医药大学学报,2021,33(6):29-32.
[19] 许永豪,罗艳霞,陈巧.大承气汤保留灌肠对脓毒症胃肠功能障碍患者炎症反应的影响[J].中西医结合研究,2021,13(6):398-401.
[20] 陈嘉琪,钱义明,钱风华,等.基于脑肠肽探讨针刺治疗急性胃肠损伤的作用机制[J].实用临床医药杂志,2021,25(18):120-123.
[21] 连佳明,钱义明,钱风华,等.针刺治疗脓毒症急性胃肠损伤的研究进展[J].中医药导报,2021,27(7):148-151.
[22] 王玉华.脓毒症胃肠功能障碍患者应用穴位电刺激的临床观察[J].实用中西医结合临床,2021,21(10):72-73.
[23] 王柳,李淑芳.中医药治疗脓毒症胃肠功能障碍研究进展[J].新中医,2021,53(13):1-4.
[24] 李仕业,李志亨,何朝.穴位电刺激改善脓毒症胃肠功能障碍患者早期肠内营养耐受性[J].世界最新医学信息文摘,2021,(26):255-256.
[25] 高天野,刘杰,黄丽英.壮医药线点灸治疗脓毒性休克急性胃肠损伤临床观察[J].中国中医药现代远程教育,2021,19(8):117-119.
[26] 李亚莉,褚玉茹,于乃浩,等.针灸改善脓毒症患者胃肠功能障碍的临床观察[J].天津中医药,2021,38(4):479-482.
[27] 陈红,欧阳红莲,潘卫红,等.中医外治法在脓毒症胃肠功能障碍中的应用进展[J].当代临床医刊,2021,34(1):85-86,58.
[28] 高天野,刘杰,黄丽英.壮医药线点灸对脓毒症急性胃肠损伤患者喂养耐受性和营养状态的影响[J].中医临床研究,2020,12(33):72-74.
[29] 孙一凡,戴林峰,袁思成,等.针刺治疗脓毒症胃肠功能障碍临床研究进展[J].中国中医药图书情报杂志,2020,44(6):68-70.
[30] 李明雁,沈云霞,万青,等.子午流注择时应用清瘟败毒饮合凉膈散加减通腑治疗脓毒症胃肠功能障碍(热毒炽盛证)的临床研究[J].中国中医急症,2020,29(8):1407-1410.
[31] 徐畅,梁建峰,李智勇,等.老年脓毒症胃肠功能障碍的中医证素分布及其针刺疗效观察[J].世界中医药,2020,15(16):2499-2502.
[32] 孙芳园,许开亮,袁林,等.电针结合穴位敷贴治疗脓毒症急性胃肠损伤的临床研究[J].中国中医急症,2020,29(7):1165-1167,1195.
[33] 容健伟,郑文诺,李春杏.药灸联合治疗脓毒症患者脾虚型胃肠功能障碍的临床疗效[J].内蒙古中医药,2020,39(2):88-90.

[34] 刘欢,朱瑾,倪海滨,等.穴位电刺激改善脓毒症胃肠功能障碍患者早期肠内营养耐受性:多中心随机对照研究[J].中国针灸,2020,40(3):229-233.
[35] 刘超,肖阳春,梁立新,等.针刺联合多潘立酮治疗脓毒症急性胃肠损伤疗效观察[J].上海针灸杂志,2020,39(1):6-10.
[36] 周建明,廖吕钊,王希,等.脓毒症急性胃肠损伤下胃肠激素胃动素和 Ghrelin 变化及针刺对其影响的研究进展[J].全科医学临床与教育,2019,(3):252-254.
[37] 李海峰,胡国强,刘雯雯,等.针刺夹脊穴对脓毒症胃肠功能障碍炎性反应指标的影响[J].中国针灸,2019,39(10):1055-1058.
[38] 刘凯,孙宏,李冀,等.腹部电针对脓毒症胃肠功能障碍的影响[J].上海针灸杂志,2019,38(10):1118-1121.
[39] 肖秋生.中药灌肠方+针刺联合常规疗法治疗 ICU 脓毒症胃肠功能障碍随机平行对照研究[J].实用中医内科杂志,2019,33(5):74-77.
[40] 张俭,黄竞,常卫东,等.中医综合疗法治疗脓毒症胃肠功能障碍临床研究[J].新中医,2019,(6):154-158.
[41] 王晓鹏,郭玉红,刘清泉.经腧穴“外治法”治疗脓毒症胃肠功能障碍的研究进展[J].中国中医急症,2019,28(6):1109-1111,1121.
[42] 陈威,李海峰,胡国强.针刺夹脊穴对脓毒血症腹腔内压、胃腔残留量疗效评估[J].上海针灸杂志,2019,38(6):601-606.
[43] 李海峰,胡国强,刘雯雯.针刺华佗夹脊穴治疗脓毒血症胃肠功能障碍临床疗效观察[J].针刺研究,2019,44(1):43-46.
[44] 张微,季春莲,许秀娟,等.电针联合足三里注射新斯的明对脓毒症胃肠功能障碍的影响[J].浙江中医杂志,2018,53(11):830.
[45] 张微,孟建标.电针联合芒硝外敷对脓毒症胃肠功能障碍的疗效[J].中国现代医生,2018,56(28):84-87.
[46] 张艳秋,马柯,董振飞.脓毒症胃肠功能损伤中医外治法近况[J].时珍国医国药,2018,29(6):1438-1439.
[47] 丁杰.中医药治疗脓毒症胃肠功能障碍概况[J].中外女性健康研究,2017,(17):4-5,16.
[48] 梁静,胡明丽,赵国良,等.穴位药物贴敷治疗脓毒症胃肠功能障碍 30 例[J].中国民间疗法,2017,25(6):16-17.
[49] 赵国桢,郭玉红,李博,等.中医药防治脓毒症的研究进展[J].中国中药杂志,2017,42(8):1423-1429.
[50] 方军,李兰,李冰冰,等.针灸对严重脓毒症患者胃肠功能障碍及胃肠激素影响分析[J].湖南中医药大学学报,2016,36(A01):11.
[51] 张利娟,张广清,吴巧媚,等.脓毒症肠功能障碍的中医外治法研究进展[J].江西中医药,2015,(11):67-69.
[52] 虞意华,金肖青,俞迈红,等.针灸对老年严重脓毒症患者胃肠功能及胃肠激素影响的临床研究[J].中华中医药学刊,2015,33(8):1953-1956.
[53] 王玲华,李艳娟,傅根连.中药鼻饲联合穴位外敷治疗机械通气患者胃肠功能障碍疗效观察[J].浙江中西医结合杂志,2015,25(1):44-46.
[54] 蔡莉娟,丁学军,刘文兵,等.电针对脓毒症患者胃肠功能障碍的干预作用[J].中国中医急症,2014,23(2):268-270.
[55] 王晶晶,孔祥照,张晓璇.针刺足三里对脓毒症胃肠功能障碍患者腹内压的影响[J].中国中医急症,2012,21(11):1834-1835.

2.8 ClinicalTrials.gov (https://clinicaltrials.gov/)
Search date:September 27, 2023 Beijing time
Filters:

Condition or disease	(Bloodstream Infection OR Bloodstream Infections OR Infection,

	Bloodstream OR Pyemia OR Pyemias OR Pyohemia OR Pyohemias OR Pyaemia OR Pyaemias OR Septicemia OR Septicemias OR Poisoning, Blood OR Blood Poisoning OR Blood Poisonings OR Poisonings, Blood OR Severe Sepsis OR Sepsis, Severe) AND (Disease, Gastrointestinal OR Diseases, Gastrointestinal OR Gastrointestinal Disease OR Gastrointestinal Disorders OR Gastrointestinal Disorder OR Functional Gastrointestinal Disorders OR Functional Gastrointestinal Disorder OR Gastrointestinal Disorder, Functional OR Gastrointestinal Disorders, Functional OR Cholera Infantum OR gastrointestinal dysfunction OR acute gastrointestinal injury)
Intervention/Treatment	Pharmacopuncture OR Acupuncture Treatment OR Acupuncture Treatments OR Treatment, Acupuncture OR Therapy, Acupuncture OR Pharmacoacupuncture Treatment OR Treatment, Pharmacoacupuncture OR Pharmacoacupuncture Therapy OR Therapy, Pharmacoacupuncture OR Acupotomy OR Acupotomies OR Acupuncture Therapy OR acupuncture

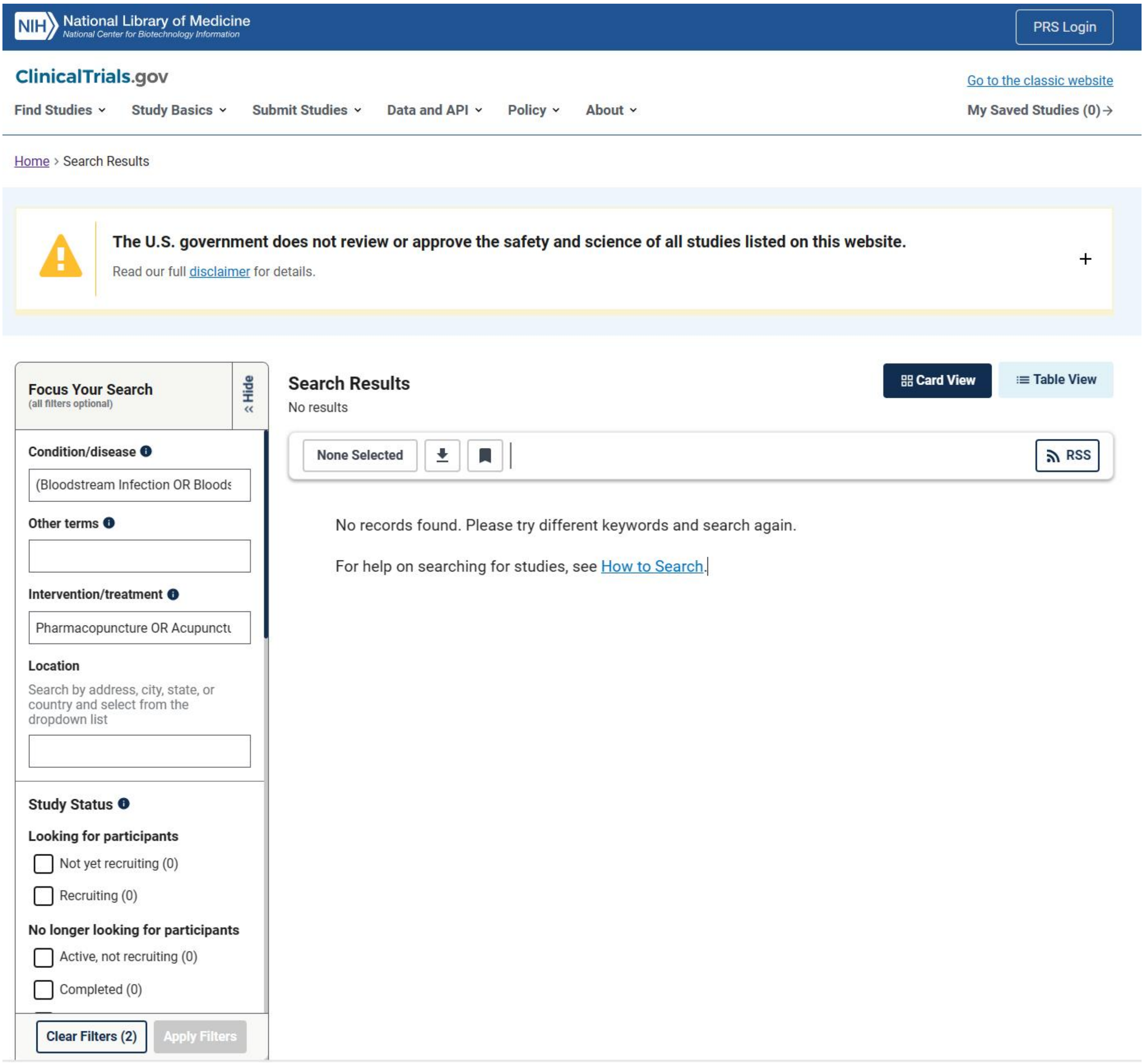

2.9 CHiCTR (http://www.chictr.org.cn)
Search date:September 27, 2023 Beijing time

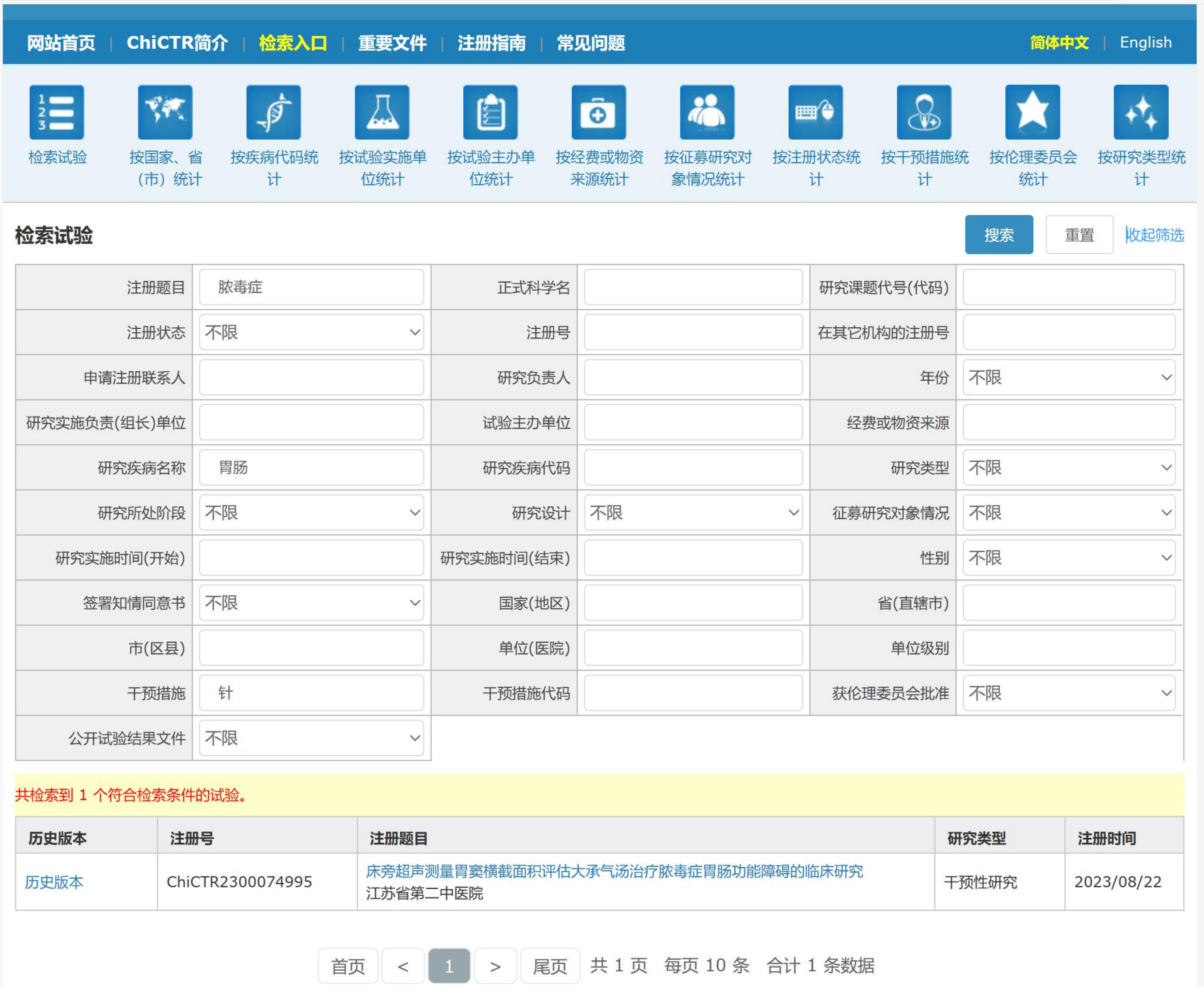

床旁超声测量胃窦横截面积评估大承气汤治疗脓毒症胃肠功能障碍的临床研究, ChiCTR2300074995, https://www.chictr.org.cn/showproj.html?proj=200323, 2023.

Supplementary File S3. A list of excluded studies by reading title and abstract.

Study design:

(1) Zhao F, Zeng J, Xian S, Lin X, Liu K, Lu L, Lin G, Wang S. Acupuncture improves paralytic ileus secondary to sepsis: a case report. Acupunct Med. 2019 Dec;37(6):372-374. doi: 10.1177/0964528419883279. Epub 2019 Nov 13. PMID: 31722545.
(2) Pacheco-Cerrato IM, Cobos-Moreno P, Castro-Avalos MA, Fernandez-la-Villa J, Morán-Cortés JF, Moran JM. Comment on "Electroacupuncture Improves Intestinal Dysfunction in Septic Patients: A Randomised Controlled Trial". Biomed Res Int. 2021 Feb 17;2021:6487272. doi: 10.1155/2021/6487272. PMID: 33688494; PMCID: PMC7914076.
(3) 床旁超声测量胃窦横截面积评估大承气汤治疗脓毒症胃肠功能障碍的临床研究, ChiCTR2300074995, https://www.chictr.org.cn/showproj.html?proj=200323, 2023.
(4) 周添奕,钱风华,黄馨云等.基于数据挖掘探究危重症患者急性胃肠损伤的中医针灸诊治规律[J].世界科学技术-中医药现代化,2019,21(08):1606-1614.

(5) 赵国桢,郭玉红,李博等.中医药防治脓毒症的研究进展[J].中国中药杂志,2017,42(08):1423-1429.DOI:10.19540/j.cnki.cjcmm.2017.0038.
(6) 张利娟,张广清,吴巧媚等.脓毒症肠功能障碍的中医外治法研究进展[J].江西中医药,2015,46(11):67-69.
(7) 袁红,徐培鑫,李航宇等.穴位注射治疗急性胰腺炎胃肠功能障碍的研究进展[J].中外医学研究,2022,20(36):165-168.DOI:10.14033/j.cnki.cfmr.2022.36.043.
(8) 于佳琪, 梁群, 刘雨默, 等. 中医药治疗脓毒症胃肠功能障碍的研究进展[J]. 中国中医急症,2023,32(3):545-547,551. DOI:10.3969/j.issn.1004-745X.2023.03.044.
(9) 杨金亮,齐文升,杨秀捷,等. 通调平衡针刺法治疗脓毒症胃肠功能障碍的理论研究[C]. //世界中医药学会联合会医院感染管理专业委员会第二届学术年会论文集. 2017:7-11.
(10)徐武兵,韩春林,泽让吉波等.中医外治法治疗脓毒症胃肠功能损伤进展[J].基层医学论坛,2020,24(11):1591-1593.DOI:10.19435/j.1672-1721.2020.11.073.
(11)王玉玉. 中医特色疗法治疗脓毒症胃肠功能障碍的研究进展[J]. 医药前沿,2017,7(32):6-8. DOI:10.3969/j.issn.2095-1752.2017.32.003.
(12)王婷玉,孟捷.针灸辅助治疗脓毒症胃肠功能障碍疗效 Meta 分析[J].辽宁中医药大学学报,2022,24(02):165-170.DOI:10.13194/j.issn.1673-842x.2022.02.037.
(13)王柳,李淑芳.中医药治疗脓毒症胃肠功能障碍研究进展[J].新中医,2021,53(13):1-4.DOI:10.13457/j.cnki.jncm.2021.13.001.
(14)王丽娟,李健,李小娟.针刺疗法治疗脓毒症临床疗效和安全性的 Meta 分析[J].中医药导报,2018,24(23):86-90.DOI:10.13862/j.cnki.cn43-1446/r.2018.23.027.
(15)田锴熙,刘毅. 针刺疗法治疗脓毒症胃肠功能障碍的研究进展[J]. 世界最新医学信息文摘（连续型电子期刊）,2023,23(53):91-97. DOI:10.3969/j.issn.1671-3141.2023.053.016.
(16)孙一凡,戴林峰,袁思成,王醒.针刺治疗脓毒症胃肠功能障碍临床研究进展[J].中国中医药图书情报杂志,2020,44(6):68-70.
(17)张小桐,蔡卫敏,刘书华.1 例脓毒症休克合并胃肠功能障碍患者的中医护理体会[J].中西医结合护理,2023,9(3):25-28.
(18)刘清泉,陈仁波,李博. 中医药补充抗生素治疗脓毒症的专家共识[C]//中国科学技术协会,吉林省人民政府.第十九届中国科协年会——分 12 标准引领中医药学术创新发展高峰论坛论文集.第十九届中国科协年会——分 12 标准引领中医药学术创新发展高峰论坛论文集,2017:1.
(19)连佳明,钱义明,钱风华等.针刺治疗脓毒症急性胃肠损伤的研究进展[J].中医药导报,2021,27(07):148-151.DOI:10.13862/j.cnki.cn43-1446/r.2021.07.027.
(20)周建明,廖吕钊,王希,李雯静,潘思旭,徐娜菲,江荣林.脓毒症急性胃肠损伤下胃肠激素胃动素和 Ghrelin 变化及针刺对其影响的研究进展[J].全科医学临床与教育,2019,17(3):252-254.
(21)黄展明. 宣肺利气法针刺对胃肠癌术后胃肠功能恢复的临床观察[D].广州中医药大学,2017.
(22)黄文婷. 穴位艾灸治疗脓毒症胃肠功能损伤的临床观察[D].北京中医药大学,2018.
(23)郭文辉,于秋香,孙玮婷等.中医外治法在 ICU 内的应用研究进展[J].世界科学技术-中医药现代化,2022,24(03):1278-1285.
(24)方军,李兰,李冰冰,等. 针灸对严重脓毒症患者胃肠功能障碍及胃肠激素影响分析[J]. 湖南中医药大学学报,2016,36(0):11.
(25)张艳秋, 马柯, 董振飞. 脓毒症胃肠功能损伤中医外治法近况[J]. 时珍国医国药,2018,29(6):1438-1439. DOI:10.3969/j.issn.1008-0805.2018.06.058.
(26)丁杰.中医药治疗脓毒症胃肠功能障碍概况[J].中外女性健康研究,2017(17):4-5+16.
(27)陈红,欧阳红莲,潘卫红,等. 中医外治法在脓毒症胃肠功能障碍中的应用进展[J]. 当代临床医刊,2021,34(1):85-86,58. DOI:10.3969/j.issn.2095-9559.2021.01.056.
(28)王晓鹏,郭玉红,刘清泉.经腧穴“外治法”治疗脓毒症胃肠功能障碍的研究进展[J].中国中医急症,2019,28(6):1109-1111,1121.
(29)梁静,胡明丽,赵国良,等.穴位药物贴敷治疗脓毒症胃肠功能障碍 30 例[J].中国民间疗

法,2017,25(6):16-17.

Intervention:

(1) Lv, Y., et al. (2021). "A study of the effect of combination of acupuncture and Chinese medicine (Ban Xia Xie Xin Decoction) on patients with sepsis-induced gastrointestinal dysfunction." Tropical Journal of Pharmaceutical Research 20(9): 1983-1989.
(2) Wang, Y., et al. (2017). "Early traditional Chinese medicine bundle therapy for the prevention of sepsis acute gastrointestinal injury in elderly patients with severe sepsis." Scientific reports 7: 46015.
(3) Tyler D. Martin, Michael S. Green, Malcolm T. Whitehead, Timothy P. Scheett, Michael J. Webster, and Geoffrey M. Hudson. 2019. Six weeks of oral Echinacea purpurea supplementation does not enhance the production of serum erythropoietin or erythropoietic status in recreationally active males with above-average aerobic fitness. Applied Physiology, Nutrition, and Metabolism. 44(7): 791-795. https://doi.org/10.1139/apnm-2018-0783.
(4) **张俭,黄竞,常卫东,张晓忠,张军,杨广,李健.中医综合疗法治疗脓毒症胃肠功能障碍临床研究[J].新中医,2019(6):154-158.**
(5) 袁金霞,赖芳,杜炯栋等.运用晁恩祥教授经验针药结合防治脓毒症胃肠功能障碍的临床探讨[J].中国中医急症,2019,28(04):664-667.
(6) 袁金霞. 针药结合法对脓毒症急性呼吸窘迫综合症患者胃肠功能影响的临床研究[D].广州中医药大学,2022.DOI:10.27044/d.cnki.ggzzu.2019.001287.
(7) 于文琦. 艾灸足三里穴缓解卒中后疲劳的临床研究[D].广州中医药大学,2018.
(8) 许永豪, 罗艳霞, 陈巧. 大承气汤保留灌肠对脓毒症胃肠功能障碍患者炎症反应的影响[J]. 中西医结合研究, 2021, 13(6):398-401. DOI:10.3969/j.issn.1674-4616.2021.06.010.
(9) **肖秋生.中药灌肠方+针刺联合常规疗法治疗 ICU 脓毒症胃肠功能障碍随机平行对照研究[J].实用中医内科杂志,2019,33(5).**
(10) 魏英凯. 针刺联合神阙穴贴敷治疗脓毒症胃肠功能障碍疗效观察[J]. 健康必读, 2021(34):86.
(11) 王益斐, 智屹惠, 江荣林, 等. 早期中医药集束化措施对预防脓毒症急性胃肠损伤的临床观察[C]. 中华医学会第 10 次全国重症医学大会论文集. 2016:349-349.
(12) **彭晓洪,宋棠,黄永莲,黄壑霏,王评.针药结合疗法对脓毒症患者肠道屏障功能保护作用的临床研究[J].中国中西医结合急救杂志,2023,30(2):142-146.**
(13) 王晶. 针刺联合大承气汤对脓毒症毒热内盛证肠道菌群的影响[D].山东中医药大学,2022.DOI:10.27282/d.cnki.gsdzu.2020.000320.
(14) **李莉,晏军,吴彩军,马林沁,郑广明,果海凤,王健,杨喆,牛丽强.针灸联合穴位贴敷法治疗急诊老年脓毒症机械通气患者继发肠功能障碍的疗效[J].中国医师杂志,2022,24(4):486-489.**
(15) 刘丹蕾. 非药物疗法对机械通气患者急性胃肠损伤的影响[D].南方医科大学,2019.
(16) 葛旭,吴迪,王国兴等.生大黄灌肠联合针灸治疗脓毒症患者胃肠功能的临床观察[J].世界中西医结合杂志,2023,18(07):1391-1395+1400.DOI:10.13935/j.cnki.sjzx.230720.
(17) 陈分乔, 中医集束化治疗防治脓毒症肠功能障碍的临床研究. 河北省,河北省中医院,2016-12-22.
(18) 常卫东. 中医综合疗法治疗脓毒症胃肠功能障碍的临床研究[D].广州中医药大学,2018.
(19) 周波巧,葛婷爱,冯晓菲.四逆汤穴位贴敷治疗脓毒症胃肠功能障碍 35 例[J].浙江中医杂志,2022,57(6):425,461.
(20) 张微,孟建标.电针联合芒硝外敷对脓毒症胃肠功能障碍的疗效[J].中国现代医生,2018,56(28):84-87.
(21) 张萍萍,周逸丹,葛婷爱,等.四逆汤穴位贴敷恢复脓毒症休克患者胃肠功能的临床研究[J].辽宁中医杂志,2023,50(6):192-194.
(22) 张萍萍,周逸丹,葛婷爱,等.四逆汤穴位贴敷联合双歧杆菌三联活菌散治疗脓毒症胃肠功能障碍临床研究[J].新中医,2022,54(21):43-46.

(23) 叶颖颖,郭黄容,温正旺.中药穴位超声导入疗法治疗重症肺炎合并胃肠功能障碍的临床观察[J].中国中医药科技,2023,30(1):123-125.
(24) 杨洁,陈微,陈美华,等.整肠散穴位贴敷对脓毒症患者胃肠功能的影响[J].蛇志,2022,34(3):350-353.
(25) 伍万,江荣林,赵利娟,等.电针联合整肠生胶囊对胃肠功能障碍脓毒症患者早期肠内营养耐受性的影响[J].浙江临床医学,2022,24(11):1590-1592.
(26) 王玉华.脓毒症胃肠功能障碍患者应用穴位电刺激的临床观察[J].实用中西医结合临床,2021,21(10):72-73.
(27) 王玲华,李艳娟,傅根连.中药鼻饲联合穴位外敷治疗机械通气患者胃肠功能障碍疗效观察[J].浙江中西医结合杂志,2015,25(1):44-46.
(28) 孙一凡,戴林峰,袁思成等.针刺治疗脓毒症胃肠功能障碍临床研究进展[J].中国中医药图书情报杂志,2020,44(06):68-70.
(29) 孙芳园,许开亮,袁林,等.电针结合穴位敷贴治疗脓毒症急性胃肠损伤的临床研究[J].中国中医急症,2020,29(7):1165-1167,1195.
(30) 容健伟,郑文诺,李春杏.药灸联合治疗脓毒症患者脾虚型胃肠功能障碍的临床疗效[J].内蒙古中医药,2020,39(2):88-90.
(31) 牛丽强,杨喆,田丽,等.吴茱萸热敷联合耳穴压丸辅助治疗脓毒症胃肠功能障碍效果观察[J].北京中医药,2023,42(2):214-217.
(32) 刘欢,朱瑾,倪海滨,等.穴位电刺激改善脓毒症胃肠功能障碍患者早期肠内营养耐受性:多中心随机对照研究[J].中国针灸,2020,40(3):229-233.
(33) 李明雁,沈云霞,万青,等.子午流注择时应用清瘟败毒饮合凉膈散加减通腑治疗脓毒症胃肠功能障碍(热毒炽盛证)的临床研究[J].中国中医急症,2020,29(8):1407-1410.
(34) 高天野,刘杰,黄丽英.壮医药线点灸治疗脓毒性休克急性胃肠损伤临床观察[J].中国中医药现代远程教育,2021,19(8):117-119.
(35) 陈文,曾奕云,何晓铭.温灸刮痧干预脓毒症胃肠功能障碍的疗效观察[J].中医外治杂志,2022,31(6):33-35.
(36) 李仕业,李志亨,何朝.穴位电刺激改善脓毒症胃肠功能障碍患者早期肠内营养耐受性[J].世界最新医学信息文摘,2021,(26):255-256.
(37) 张微,季春莲,许秀娟,等.电针联合足三里注射新斯的明对脓毒症胃肠功能障碍的影响[J].浙江中医杂志,2018,53(11):830.

Participant:

(1) Fan Q, Lei C, Wang Y, Yu N, Wang L, Fu J, Dong H, Lu Z, Xiong L. Transcutaneous Electrical Acupoint Stimulation Combined With Auricular Acupressure Reduces Postoperative Delirium Among Elderly Patients Following Major Abdominal Surgery: A Randomized Clinical Trial. Front Med (Lausanne). 2022 Jun 15;9:855296. doi: 10.3389/fmed.2022.855296. PMID: 35783617; PMCID: PMC9240658.
(2) 王晶晶,孔祥照,张晓璇.针刺足三里对脓毒症胃肠功能障碍患者腹内压的影响[J].中国中医急症,2012,21(11):1834-1835.
(3) 高江宝,罗伟君,李炜明等.基于肺与大肠相表里探讨早期防治胃肠功能障碍对重症肺炎的影响[J].江西中医药大学学报,2021,33(06):29-32.

Awaiting classification:

(1) 张笑言. 基于"肺与大肠相表里"理论针刺治疗脓毒症胃肠功能障碍的临床研究[D].黑龙江中医药大学,2023.DOI:10.27127/d.cnki.ghlzu.2023.000743.
(2) 虞意华,金肖青,俞迈红等.针灸对老年严重脓毒症患者胃肠功能及胃肠激素影响的临床研究[J].中华中医药学刊,2015,33(08):1953-1956.DOI:10.13193/j.issn.1673-7717.2015.08.048.
(3) 杨卉卉. 针刺治疗脓毒症患者脾胃虚弱型胃肠功能障碍的临床研究[D]. 山东:山东中医药大

学, 2018.
(4) 徐畅,梁建峰,李智勇,董海山,尹超群.老年脓毒症胃肠功能障碍的中医证素分布及其针刺疗效观察[J].世界中医药,2020,15(16):2499-2502.
(5) 韦志友. 复元针法治疗脓毒症患者急性胃肠损伤的临床观察[D].北京中医药大学,2017.
(6) 王晓鹏. 针刺辅助治疗脓毒症胃肠功能障碍的随机对照临床研究[D].北京中医药大学,2019.
(7) 王丽娟. 腹针治疗脓毒症急性胃肠损伤的临床研究[D].广州中医药大学,2020.DOI:10.27044/d.cnki.ggzzu.2019.000892.
(8) 孙一凡. 基于气机升降理论运用电针干预脓毒症患者急性胃肠损伤的临床研究[D].南京中医药大学,2022.DOI:10.27253/d.cnki.gnjzu.2021.000220.
(9) 孙德阳,杨洋,杜纯鹏等.电针治疗脓毒症胃肠功能障碍的临床研究[J].针灸临床杂志,2019,35(10):47-50.
(10) 彭小菊,钟迪,何琪.早期肠内营养配合复元针法治疗脓毒症急性胃肠损伤的疗效观察[J].中国中西医结合消化杂志,2022,30(12):838-843.
(11) 刘凯,孙宏,李冀等.腹部电针对脓毒症胃肠功能障碍的影响[J].上海针灸杂志,2019,38(10):1118-1121.DOI:10.13460/j.issn.1005-0957.2019.10.1118.
(12) 刘欢. 电针对重症患者胃肠功能障碍的疗效观察[D].南京中医药大学,2022.DOI:10.27253/d.cnki.gnjzu.2021.000724.
(13) 连佳明,钱义明,李璟,钱风华,陈嘉琪,陈晓桐,丁纯蕾.益气健脾针法修复急性胃肠损伤肠道屏障的临床研究[J].中国中医急症,2021,30(12):2119-2122.
(14) 李亚莉, 褚玉茹, 于乃浩, 等. 针灸改善脓毒症患者胃肠功能障碍的临床观察[J]. 天津中医药, 2021, 38(4):479-482. DOI:10.11656/j.issn.1672-1519.2021.04.15.
(15) 李海峰,胡国强,刘雯雯等.针刺夹脊穴对脓毒症胃肠功能障碍炎性反应指标的影响[J].中国针灸,2019,39(10):1055-1058.DOI:10.13703/j.0255-2930.2019.10.006.
(16) 李海峰,胡国强,刘雯雯. 针刺华佗夹脊穴治疗脓毒血症胃肠功能障碍临床疗效观察[J]. 针刺研究,2019,44(1):43-46. DOI:10.13702/j.1000-0607.170579.
(17) 胡国强, 脊神经定位结合针刺华佗夹脊穴取穴治疗脓毒血症胃肠功能障碍临床疗效研究. 浙江省,长兴县中医院,2020-10-10.
(18) 刘超, 肖阳春, 梁立新, 等. 针刺联合多潘立酮治疗脓毒症急性胃肠损伤疗效观察[J]. 上海针灸杂志, 2020, 39(1):6-10. DOI:10.13460/j.issn.1005-0957.2020.01.0006.
(19) 陈嘉琪,钱义明,钱风华等.基于脑肠肽探讨针刺治疗急性胃肠损伤的作用机制[J].实用临床医药杂志,2021,25(18):120-123.
(20) c 学,2022.DOI:10.26973/d.cnki.gbjzu.2021.000715.
(21) 陈威,李海峰,胡国强.针刺夹脊穴对脓毒血症腹腔内压、胃腔残留量疗效评估[J].上海针灸杂志,2019,38(06):601-606.DOI:10.13460/j.issn.1005-0957.2019.06.0601.
(22) 常乐. 脓毒症胃肠功能障碍证型分布及针刺疗效研究[D].天津中医药大学,2022.DOI:10.27368/d.cnki.gtzyy.2021.000307.
(23) 蔡莉娟,丁学军,刘文兵等.电针对脓毒症患者胃肠功能障碍的干预作用[J].中国中医急症,2014,23(02):268-270.
(24) Meng, J.B., et al., Electro-acupuncture attenuates inflammatory responses and intraabdominal pressure in septic patients. Medicine (United States), 2018. 97(17). https://doi.org/10.1097/MD.0000000000010555.
(25) L Ban, Y Pu, H Huang, B You, W Chen, Y Wang. Acupuncture Enhances Gastrointestinal Motility and Improves Autonomic Nervous Function in Patients with Septic Gastrointestinal Dysfunction. Computational and mathematical methods in medicine, 2022, 2022, 1653290.

Supplementary File S4. A list of excluded studies by reading full text.

Study design:

(1) 胡国强, 脊神经定位结合针刺华佗夹脊穴取穴治疗脓毒血症胃肠功能障碍临床疗效研究. 浙江省, 长兴县中医院,2020-10-10.

Participant:

(1) 虞意华,金肖青,俞迈红等.针灸对老年严重脓毒症患者胃肠功能及胃肠激素影响的临床研究[J].中华中医药学刊,2015,33(08):1953-1956.DOI:10.13193/j.issn.1673-7717.2015.08.048.
(2) 徐畅,梁建峰,李智勇,董海山,尹超群.老年脓毒症胃肠功能障碍的中医证素分布及其针刺疗效观察[J].世界中医药,2020,15(16):2499-2502.
(3) 孙德阳,杨洋,杜纯鹏等.电针治疗脓毒症胃肠功能障碍的临床研究[J].针灸临床杂志,2019,35(10):47-50.
(4) 刘凯,孙宏,李冀等.腹部电针对脓毒症胃肠功能障碍的影响[J].上海针灸杂志,2019,38(10):1118-1121.DOI:10.13460/j.issn.1005-0957.2019.10.1118.
(5) 刘欢. 电针对重症患者胃肠功能障碍的疗效观察[D].南京中医药大学,2022.DOI:10.27253/d.cnki.gnjzu.2021.000724.
(6) 张笑言. 基于"肺与大肠相表里"理论针刺治疗脓毒症胃肠功能障碍的临床研究[D].黑龙江中医药大学,2023.DOI:10.27127/d.cnki.ghlzu.2023.000743.
(7) 李海峰,胡国强,刘雯雯等.针刺夹脊穴对脓毒症胃肠功能障碍炎性反应指标的影响[J].中国针灸,2019,39(10):1055-1058.DOI:10.13703/j.0255-2930.2019.10.006.
(8) 李海峰,胡国强,刘雯雯. 针刺华佗夹脊穴治疗脓毒血症胃肠功能障碍临床疗效观察[J]. 针刺研究,2019,44(1):43-46. DOI:10.13702/j.1000-0607.170579.
(9) 陈嘉琪,钱义明,钱风华等.基于脑肠肽探讨针刺治疗急性胃肠损伤的作用机制[J].实用临床医药杂志,2021,25(18):120-123.
(10) 陈威,李海峰,胡国强.针刺夹脊穴对脓毒血症腹腔内压、胃腔残留量疗效评估[J].上海针灸杂志,2019,38(06):601-606.DOI:10.13460/j.issn.1005-0957.2019.06.0601.
(11) 蔡莉娟,丁学军,刘文兵等.电针对脓毒症患者胃肠功能障碍的干预作用[J].中国中医急症,2014,23(02):268-270.

Supplementary File S5. Acupuncture prescription in the included studies.

Study	Selection of Acupoints	Acupuncture therapy	Frequency of intervention	Duration of acupuncture treatment
Wei, 2017	Shangwan (CV13), Zhongwan (CV12), Xiawan (CV10), bilateral Tianshu (ST25) points, bilateral Zusanli (ST36) points, and bilateral Yanglingquan (GB34) points	The acupuncture session was sustained for a duration of 20 to 30 minutes after the application of specific techniques.	Qd	28 days, or until the patient either succumbs or reaches a state of recovery.
Meng 2018	Zusanli (ST36) points and Shangjuxu(ST37)	EA stimulation with a continuous wave, a frequency of 4Hz, and the intensity was adjusted to induce visible muscle twitching for the duration of the 20-min EA period.	Bid	5 days.
Yang, 2018	Zhongwan (CV 12), bilateral Tianshu (ST 25), Guanyuan (CV 4), bilateral Zusanli (ST 36), bilateral Shangjuxu (ST 37), bilateral Xiajuxu (ST 39) and bilateral Neiguan (PC 6)	The acupuncture session was sustained for a duration of 30 minutes after the application of specific techniques.	Bid	7 days
Wang XP, 2019	Shangwan (CV 13), Zhongwan (CV 12), Xiawan (CV 10), bilateral Tianshu (ST 25), bilateral Zusanli (ST 36) and bilateral Yanglingquan (GB 34)	The acupuncture session was sustained for a duration of 30 minutes after the application of specific techniques.	Qd	7 days
Liu et al., 2020	Shangwan (CV 13), Zhongwan (CV 12), Xiawan (CV 10), Qihai (CV 6), bilateral Tianshu (ST 25), Neiguan (PC 6) and Zusanli (ST 36)	The acupuncture session was sustained for a duration of 20 to 30 minutes after the application of specific techniques.	Qd	7 days
Wang LJ, 2019	Zhongwan (CV12), Xiawan (CV10), Qihai (CV6), Guanyuan (CV4), bilateral Huaroumen (ST24), Wailing (ST26), bilateral Daheng (SP15) and bilateral Tianshu (ST25)	The acupuncture session was sustained for a duration of 30 minutes without any specific techniques.	Qd	5 days
Li et al., 2021	Zhongwan (CV 12), Xiawan (CV 10), bilateral Tianshu (ST 25), Guanyuan (CV 4), Qihai (CV 6), bilateral Zusanli (ST 36) and	The acupuncture session was sustained for a duration of 20 minutes after the application of	Qd	7 days

	bilateral Gongsun (SP 6)	specific techniques.		
Lian et al., 2021	Shangwan (CV 13), Zhongwan (CV 12), Xiawan (CV 10), Qihai (CV 6), Guanyuan (CV 4), bilateral Zusanli (ST 36), Shangjuxu (ST 37),Xiajuxu (ST 39), bilateral Tianshu (ST 25) and Daheng (SP 15)	The acupuncture treatment involved inserting needles into specific acupoints, including Shangwan, Zhongwan, Xiawan, Qihai, Guanyuan, bilateral Zusanli points, Shangjuxu point and Xiajuxu point for 30 minutes. Electroacupuncture therapy utilized dense-disperse waves to stimulate bilateral Tianshu and Daheng points at a frequency of 2 Hz (output intensity adjusted based on patient tolerance), with the needles retained for 30 minutes.	Qd	7 days
Ban et al., 2022	Shangwan (CV 13), Zhongwan (CV 12), Xiawan(CV 10), bilateral Tianshu(ST 25), Guanyuan(CV 4), Qihai(CV 6), bilateral Zusanli(ST 36), and bilateral Gongsun（SP4）	The acupuncture session was sustained for a duration of 30 minutes without any specific techniques.	Qd	7 days
Chang et al., 2022	Key acupoints included Zusanli(ST 36), Zhongwan(CV 12), Neiguan(PC 6), and Tianshu(ST 25); additional acupoints were selected based on the specific condition of the disease: for constipation, shangjuxu(ST 37) and Zigou(SJ 6) points were recommended; for diarrhea, a combination of Shangjuxu(ST 37) and Yinlingquan(GB34) points were administrated; for phlegm-dampness affecting digestion, Fenglong(ST 40), Neiting,(ST 44) and Quchi(LI 11) points were combined; for stagnation of stomach heat qi,	The acupuncture session was sustained for a duration of 20 to 30 minutes after the application of specific techniques.	Qd	7 days

	Neiting(ST 44) and Danzhong(CV 17) points were used together; Xuehai(SP 10) point was selected in treating blood stasis; Qihai(CV 6) and Guanyuan(CV 4) points were combined for weakness of spleen and stomach.			
Cheng et al., 2022	Key acupoints included Zhongwan(CV12), Tianshu(ST25), Zusanli(ST36), Shangjuxu(ST37), and Xiajuxu(ST39); additional acupoints were selected based on the specific condition of the disease: for positive syndrome of TCM, Quchi(LI11) was chosen as a pairing point, while Qihai(CV6) was selected for deficiency syndrome. For the treatment of vomiting symptoms, Neiguan(PC6) acupoint was chosen; whereas for diarrhea symptoms, Yinlingquan(SP9) acupoint was selected.	The acupuncture session was sustained for a duration of 20 minutes after the application of specific techniques.	Qd	7 days
Peng et al., 2022	Shangwan (CV13), Zhongwan (CV12), Xiawan (CV10), bilateral Tianshu (ST25) points, bilateral Zusanli (ST36) points, and bilateral Yanglingquan (GB34) points	The acupuncture session was sustained for a duration of 25 minutes after the application of specific techniques.	Qd	14 days
Sun et al., 2022	Shangwan(CV13), Guanyuan (CV 4), Zhongwan (CV 12), Qihai (CV 6), bilateral Zusanli (ST 36), Tianshu (ST 25), Shangjuxu (ST37), Sanyinjiao (SP 6), Yinlingquan (SP 9), Hegu (LI 4), Taichong (LR 3)	After needle insertion, a medium-intensity dense and sparse wave electroacupuncture device was used for the treatment of Zusanli, Tianshu, and Sanyinjiao acupoints, with each session lasting 20 minutes.	Qd	14 days

Abbreviations: d,day; Qd, quaque die；EA, Electro-acupuncture; Bid, bis in die;

Supplementary File S6. Results of subgroup analysis

6.1 Subgroup analysis of the IAP according to the baseline of APACHE II score.

	Experimental			Control				Std. Mean Difference
Study or Subgroup	Mean	SD	Total	Mean	SD	Total	Weight	IV, Random, 95% CI
**3.1.1 APACHE II ≥22**								
Wei, 2017	12.4	4.7	20	11.2	2.2	21	8.1%	0.32 [-0.29, 0.94]
Yang, 2018	8.62	2.55	41	11.25	2.72	41	9.6%	-0.99 [-1.45, -0.53]
Wang XP, 2019	11.08	1.96	20	12.96	1.51	20	7.6%	-1.05 [-1.72, -0.39]
Sun et al., 2022	11.03	1.42	27	11.19	1.03	26	8.8%	-0.13 [-0.67, 0.41]
**Subtotal (95% CI)**			**108**			**108**	**34.1%**	**-0.47 [-1.11, 0.18]**
Heterogeneity: Tau² = 0.35; Chi² = 15.67, df = 3 (P = 0.001); I² = 81%								
Test for overall effect: Z = 1.42 (P = 0.16)								
**3.1.2 APACHE II <22**								
Meng 2018	8.62	2.55	41	11.25	2.72	41	9.6%	-0.99 [-1.45, -0.53]
Wang LJ, 2019	6.82	1.47	27	7.39	2.04	28	8.9%	-0.32 [-0.85, 0.22]
Li et al., 2021	11.06	1.55	28	12.06	2.09	29	8.9%	-0.53 [-1.06, -0.01]
Lian et al., 2021	9.11	3.23	35	12.82	2.68	35	9.0%	-1.24 [-1.75, -0.72]
Ban et al., 2022	10.63	1.62	37	12.48	2.11	36	9.3%	-0.97 [-1.46, -0.49]
Peng et al., 2022	4.13	0.89	65	5.63	1.14	65	10.2%	-1.46 [-1.85, -1.07]
**Subtotal (95% CI)**			**233**			**234**	**55.9%**	**-0.94 [-1.28, -0.59]**
Heterogeneity: Tau² = 0.13; Chi² = 15.50, df = 5 (P = 0.008); I² = 68%								
Test for overall effect: Z = 5.32 (P < 0.00001)								
**3.1.3 APACHE II not reported**								
Liu et al., 2020	8.21	1.55	49	9.47	2.04	49	10.0%	-0.69 [-1.10, -0.28]
**Subtotal (95% CI)**			**49**			**49**	**10.0%**	**-0.69 [-1.10, -0.28]**
Heterogeneity: Not applicable								
Test for overall effect: Z = 3.31 (P = 0.0009)								
**Total (95% CI)**			**390**			**391**	**100.0%**	**-0.75 [-1.05, -0.45]**
Heterogeneity: Tau² = 0.19; Chi² = 39.30, df = 10 (P < 0.0001); I² = 75%								
Test for overall effect: Z = 4.95 (P < 0.00001)								
Test for subgroup differences: Chi² = 1.91, df = 2 (P = 0.38), I² = 0%								

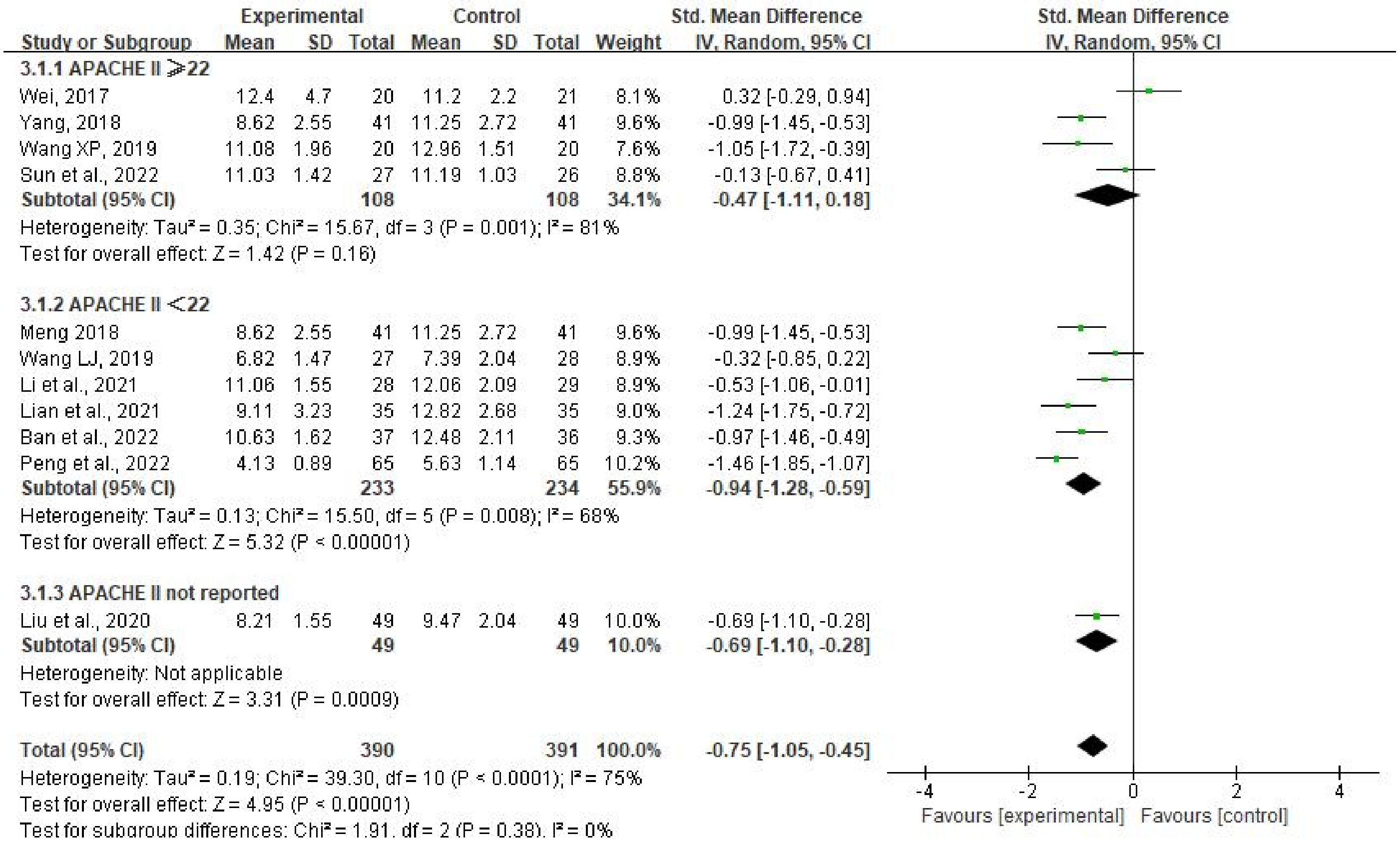

6.2 Subgroup analysis of the IAP according to the acupuncture duration.

Study or Subgroup	Experimental Mean	Experimental SD	Experimental Total	Control Mean	Control SD	Control Total	Weight	Std. Mean Difference IV, Random, 95% CI
**3.2.1 IAP: acupuncture duration = 5 days**								
Meng 2018	8.62	2.55	41	11.25	2.72	41	9.6%	-0.99 [-1.45, -0.53]
Wang LJ, 2019	6.82	1.47	27	7.39	2.04	28	8.9%	-0.32 [-0.85, 0.22]
**Subtotal (95% CI)**			**68**			**69**	**18.6%**	**-0.67 [-1.32, -0.01]**
Heterogeneity: Tau² = 0.16; Chi² = 3.52, df = 1 (P = 0.06); I² = 72%								
Test for overall effect: Z = 1.98 (P = 0.05)								
**3.2.2 IAP: acupuncture duraton =7 days**								
Ban et al., 2022	10.63	1.62	37	12.48	2.11	36	9.4%	-0.97 [-1.46, -0.49]
Yang, 2018	12.13	3.12	30	13.57	2.06	30	9.1%	-0.54 [-1.05, -0.02]
Wang XP, 2019	11.08	1.96	20	12.96	1.51	20	7.7%	-1.05 [-1.72, -0.39]
Liu et al., 2020	8.21	1.55	49	9.47	2.04	49	10.1%	-0.69 [-1.10, -0.28]
Li et al., 2021	6.82	1.47	27	7.39	2.04	28	8.9%	-0.32 [-0.85, 0.22]
Lian et al., 2021	11.06	1.55	28	12.06	2.09	29	9.0%	-0.53 [-1.06, -0.01]
**Subtotal (95% CI)**			**191**			**192**	**54.1%**	**-0.67 [-0.88, -0.46]**
Heterogeneity: Tau² = 0.00; Chi² = 5.00, df = 5 (P = 0.42); I² = 0%								
Test for overall effect: Z = 6.36 (P < 0.00001)								
**3.2.3 IAP: acupuncture duraton =14 days**								
Peng et al., 2022	4.13	0.89	65	5.63	1.14	65	10.3%	-1.46 [-1.85, -1.07]
Sun et al., 2022	11.03	1.42	27	11.19	1.03	26	8.9%	-0.13 [-0.67, 0.41]
**Subtotal (95% CI)**			**92**			**91**	**19.2%**	**-0.81 [-2.11, 0.50]**
Heterogeneity: Tau² = 0.83; Chi² = 15.43, df = 1 (P < 0.0001); I² = 94%								
Test for overall effect: Z = 1.21 (P = 0.23)								
**3.2.4 IAP: acupuncture duraton = 28 days**								
Wei, 2017	12.4	4.7	20	11.2	2.2	21	8.1%	0.32 [-0.29, 0.94]
**Subtotal (95% CI)**			**20**			**21**	**8.1%**	**0.32 [-0.29, 0.94]**
Heterogeneity: Not applicable								
Test for overall effect: Z = 1.03 (P = 0.30)								
**Total (95% CI)**			**371**			**373**	**100.0%**	**-0.63 [-0.92, -0.33]**
Heterogeneity: Tau² = 0.18; Chi² = 38.17, df = 10 (P < 0.0001); I² = 74%								
Test for overall effect: Z = 4.13 (P < 0.0001)								
Test for subgroup differences: Chi² = 9.17, df = 3 (P = 0.03), I² = 67.3%								

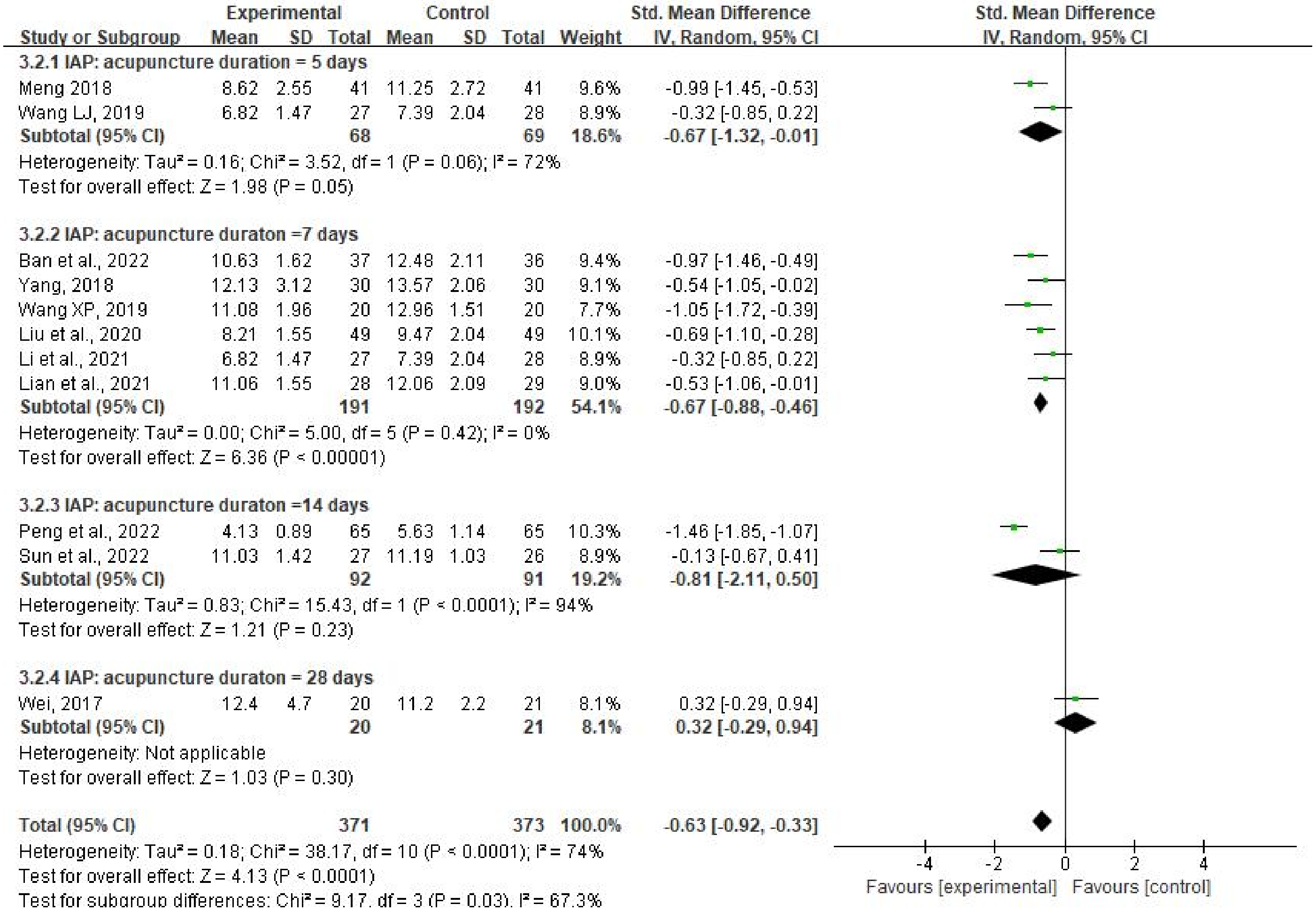

6.3 Subgroup analysis of the IAP according to the methods of acupuncture.

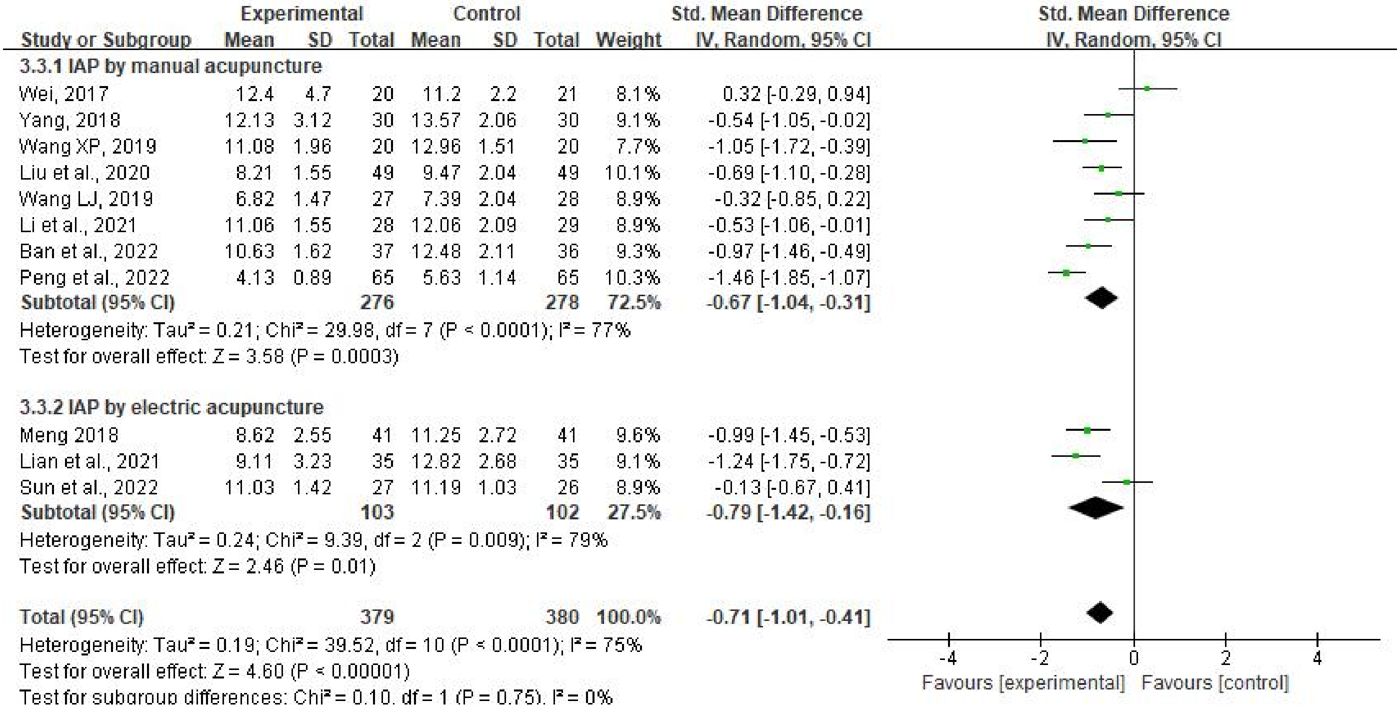

6.4 Subgroup analysis of the mortality at day 28 according to the baseline of APACHE II.

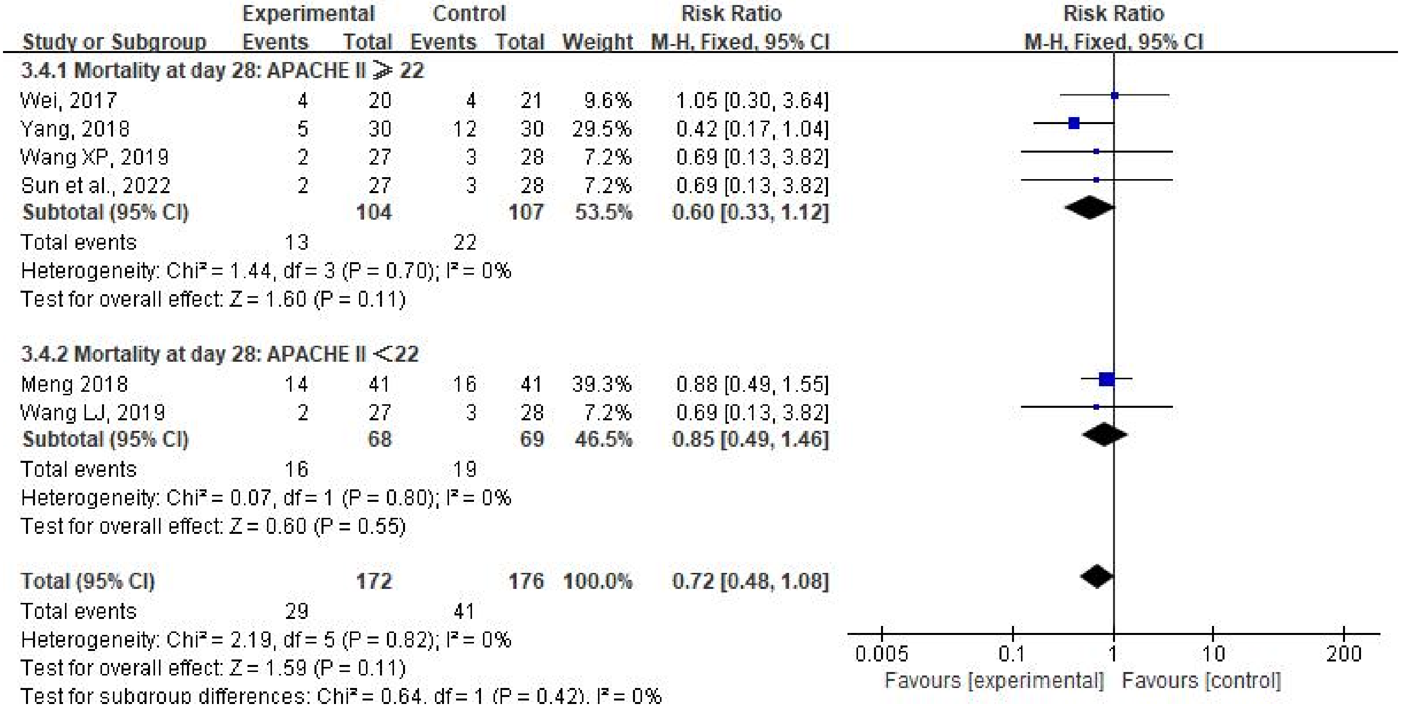

6.5 Subgroup analysis of the mortality at day 28 according to different duration of acupuncture

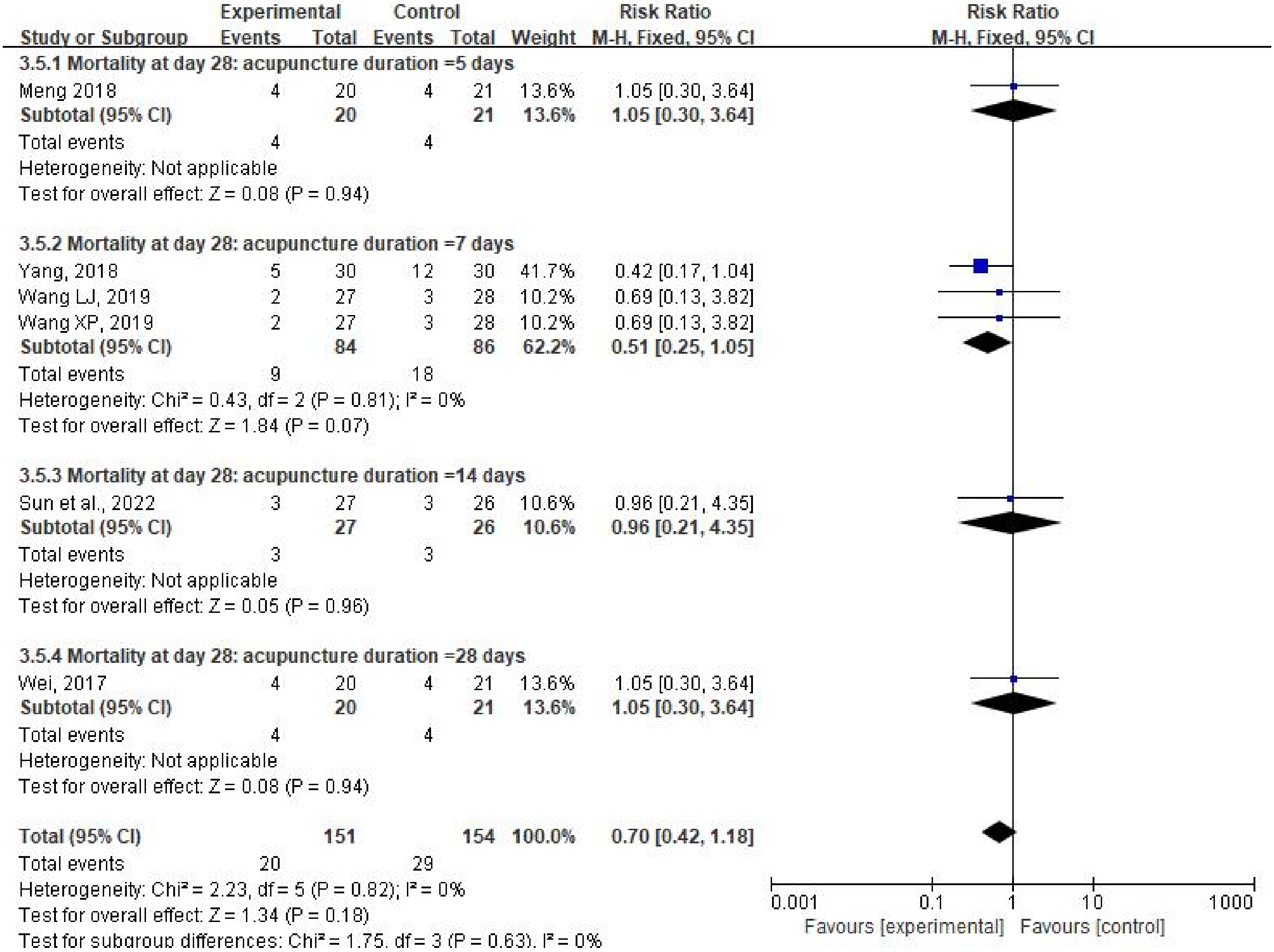

6.6 Subgroup analysis of the mortality at day 28 according to the different methods of acupuncture

Study or Subgroup	Experimental Events	Experimental Total	Control Events	Control Total	Weight	Risk Ratio M-H, Fixed, 95% CI
**3.6.1 Mortality at day 28: manual acupuncture**						
Wei, 2017	4	20	4	21	9.6%	1.05 [0.30, 3.64]
Yang, 2018	5	30	12	30	29.4%	0.42 [0.17, 1.04]
Wang XP, 2019	2	27	3	28	7.2%	0.69 [0.13, 3.82]
Wang LJ, 2019	2	27	3	28	7.2%	0.69 [0.13, 3.82]
**Subtotal (95% CI)**		**104**		**107**	**53.3%**	**0.60 [0.33, 1.12]**
Total events	13		22			
Heterogeneity: $Chi^2 = 1.44$, df = 3 (P = 0.70); $I^2 = 0\%$						
Test for overall effect: Z = 1.60 (P = 0.11)						
**3.6.2 Electro acupuncture**						
Meng 2018	14	41	16	41	39.2%	0.88 [0.49, 1.55]
Sun et al., 2022	3	27	3	26	7.5%	0.96 [0.21, 4.35]
**Subtotal (95% CI)**		**68**		**67**	**46.7%**	**0.89 [0.52, 1.52]**
Total events	17		19			
Heterogeneity: $Chi^2 = 0.01$, df = 1 (P = 0.91); $I^2 = 0\%$						
Test for overall effect: Z = 0.43 (P = 0.67)						
**Total (95% CI)**		**172**		**174**	**100.0%**	**0.74 [0.49, 1.11]**
Total events	30		41			
Heterogeneity: $Chi^2 = 2.29$, df = 5 (P = 0.81); $I^2 = 0\%$						
Test for overall effect: Z = 1.47 (P = 0.14)						
Test for subgroup differences: $Chi^2 = 0.85$, df = 1 (P = 0.36), $I^2 = 0\%$						

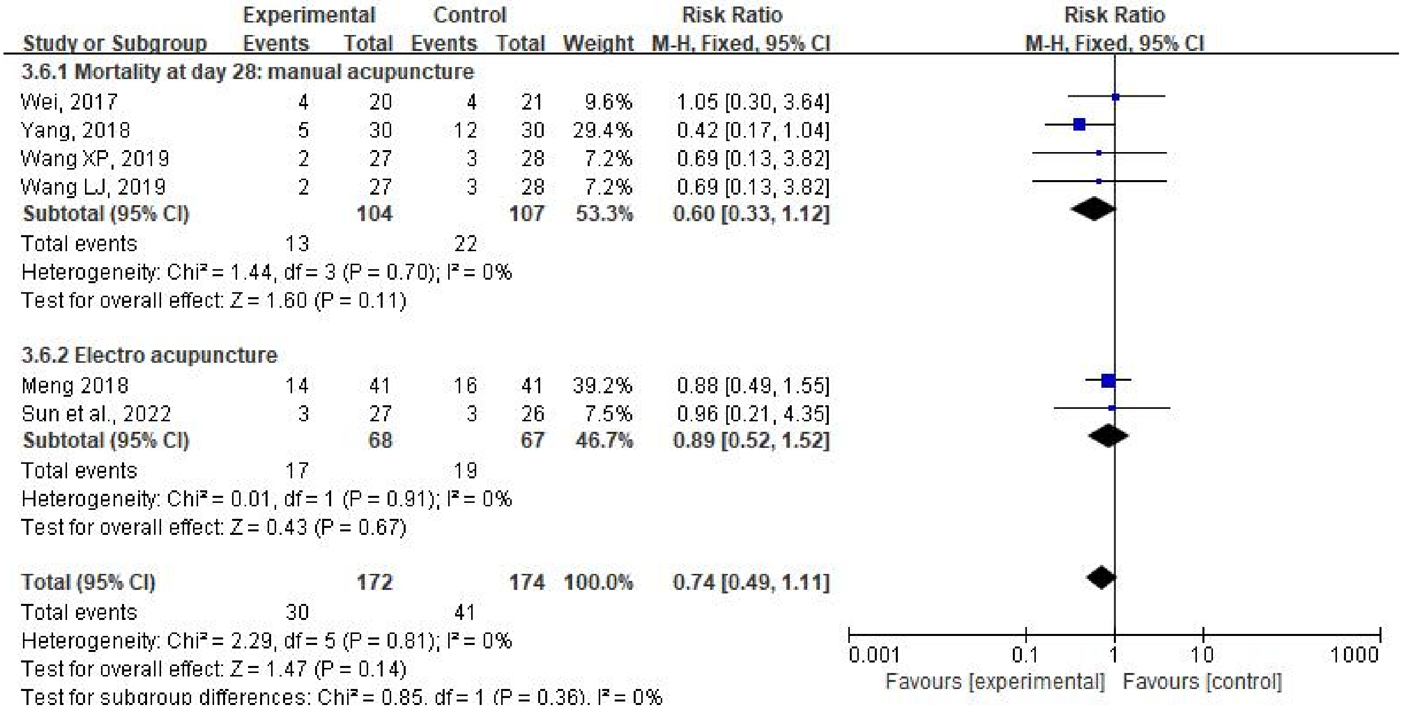

6.7 Subgroup analysis of the AGI grade according to the baseline of APACHE II

Study or Subgroup	Experimental Mean	Experimental SD	Experimental Total	Control Mean	Control SD	Control Total	Weight	Mean Difference IV, Random, 95% CI	Year
**3.7.1 AGI grade: APACHE II ⩾22**									
Wang XP, 2019	1.32	0.62	20	1.84	0.66	20	13.6%	-0.52 [-0.92, -0.12]	
Chang et al., 2022	1.85	0.59	20	2.6	0.88	20	11.5%	-0.75 [-1.21, -0.29]	2022
Cheng, 2022	1.38	0.56	29	1.96	0.55	27	17.6%	-0.58 [-0.87, -0.29]	2022
**Subtotal (95% CI)**			**69**			**67**	**42.7%**	**-0.60 [-0.81, -0.39]**	
Heterogeneity: $Tau^2 = 0.00$; $Chi^2 = 0.57$, df = 2 (P = 0.75); $I^2 = 0\%$									
Test for overall effect: Z = 5.60 (P < 0.00001)									
**3.7.2 AGI grade: APACHE II <22**									
Wang LJ, 2019	0.41	0.57	27	0.71	0.6	28	16.8%	-0.30 [-0.61, 0.01]	
Lian et al., 2021	1.54	0.74	35	2.09	0.85	35	14.4%	-0.55 [-0.92, -0.18]	2021
Peng et al., 2022	1.29	0.14	65	1.48	0.19	65	26.1%	-0.19 [-0.25, -0.13]	2022
**Subtotal (95% CI)**			**127**			**128**	**57.3%**	**-0.28 [-0.47, -0.09]**	
Heterogeneity: $Tau^2 = 0.01$; $Chi^2 = 3.89$, df = 2 (P = 0.14); $I^2 = 49\%$									
Test for overall effect: Z = 2.93 (P = 0.003)									
**Total (95% CI)**			**196**			**195**	**100.0%**	**-0.44 [-0.65, -0.23]**	
Heterogeneity: $Tau^2 = 0.04$; $Chi^2 = 17.31$, df = 5 (P = 0.004); $I^2 = 71\%$									
Test for overall effect: Z = 4.11 (P < 0.0001)									
Test for subgroup differences: $Chi^2 = 4.93$, df = 1 (P = 0.03), $I^2 = 79.7\%$									

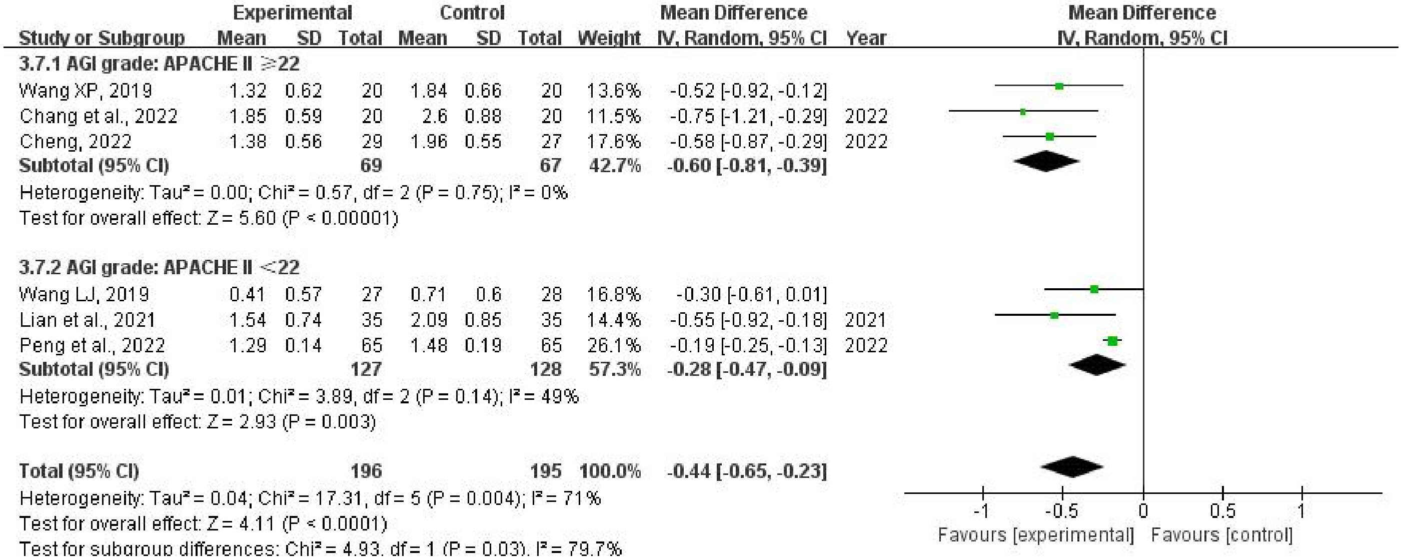

6.8 Subgroup analysis of the AGI grade according to the duration of acupuncture

Study or Subgroup	Experimental Mean	Experimental SD	Experimental Total	Control Mean	Control SD	Control Total	Weight	Mean Difference IV, Random, 95% CI	Year
**3.8.1 AGI grade: acupuncture duration =5 days**									
Wang LJ, 2019	0.41	0.57	27	0.71	0.6	28	16.8%	-0.30 [-0.61, 0.01]	
**Subtotal (95% CI)**			**27**			**28**	**16.8%**	**-0.30 [-0.61, 0.01]**	
Heterogeneity: Not applicable									
Test for overall effect: Z = 1.90 (P = 0.06)									
**3.8.2 AGI grade: acupuncture duration = 7 days**									
Wang XP, 2019	1.32	0.62	20	1.84	0.66	20	13.6%	-0.52 [-0.92, -0.12]	
Lian et al., 2021	1.54	0.74	35	2.09	0.85	35	14.4%	-0.55 [-0.92, -0.18]	2021
Chang et al., 2022	1.38	0.56	29	1.96	0.55	27	17.6%	-0.58 [-0.87, -0.29]	2022
Cheng, 2022	1.85	0.59	20	2.6	0.88	20	11.5%	-0.75 [-1.21, -0.29]	2022
**Subtotal (95% CI)**			**104**			**102**	**57.1%**	**-0.59 [-0.77, -0.40]**	
Heterogeneity: Tau² = 0.00; Chi² = 0.62, df = 3 (P = 0.89); I² = 0%									
Test for overall effect: Z = 6.29 (P < 0.00001)									
**3.8.3 AGI grade: acupuncuture duration = 14 days**									
Peng et al., 2022	1.29	0.14	65	1.48	0.19	65	26.1%	-0.19 [-0.25, -0.13]	2022
**Subtotal (95% CI)**			**65**			**65**	**26.1%**	**-0.19 [-0.25, -0.13]**	
Heterogeneity: Not applicable									
Test for overall effect: Z = 6.49 (P < 0.00001)									
**Total (95% CI)**			**196**			**195**	**100.0%**	**-0.44 [-0.65, -0.23]**	
Heterogeneity: Tau² = 0.04; Chi² = 17.31, df = 5 (P = 0.004); I² = 71%									
Test for overall effect: Z = 4.11 (P < 0.0001)									
Test for subgroup differences: Chi² = 16.69, df = 2 (P = 0.0002), I² = 88.0%									

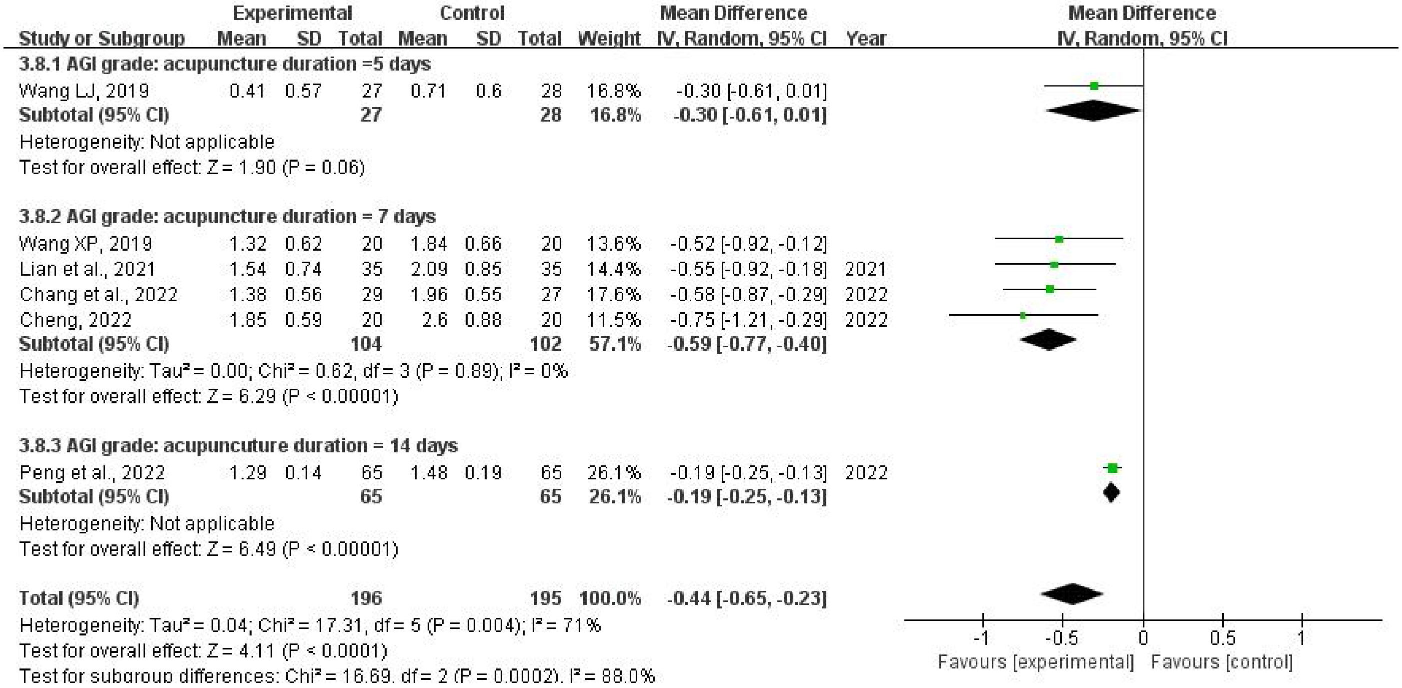

6.9 Subgroup analysis of the AGI grade according to different methods of acupuncture

Study or Subgroup	Experimental Mean	Experimental SD	Experimental Total	Control Mean	Control SD	Control Total	Weight	Mean Difference IV, Random, 95% CI	Year
**3.9.1 AGI grade: manual acupuncture**									
Wang XP, 2019	1.32	0.62	20	1.84	0.66	20	13.6%	-0.52 [-0.92, -0.12]	
Wang LJ, 2019	0.41	0.57	27	0.71	0.6	28	16.8%	-0.30 [-0.61, 0.01]	
Peng et al., 2022	1.29	0.14	65	1.48	0.19	65	26.1%	-0.19 [-0.25, -0.13]	2022
Chang et al., 2022	1.38	0.56	29	1.96	0.55	27	17.6%	-0.58 [-0.87, -0.29]	2022
Cheng, 2022	1.85	0.59	20	2.6	0.88	20	11.5%	-0.75 [-1.21, -0.29]	2022
**Subtotal (95% CI)**			**161**			**160**	**85.6%**	**-0.42 [-0.65, -0.19]**	
Heterogeneity: Tau² = 0.04; Chi² = 14.39, df = 4 (P = 0.006); I² = 72%									
Test for overall effect: Z = 3.62 (P = 0.0003)									
**3.9.2 AGI grade: electro acupuncture**									
Lian et al., 2021	1.54	0.74	35	2.09	0.85	35	14.4%	-0.55 [-0.92, -0.18]	2021
**Subtotal (95% CI)**			**35**			**35**	**14.4%**	**-0.55 [-0.92, -0.18]**	
Heterogeneity: Not applicable									
Test for overall effect: Z = 2.89 (P = 0.004)									
**Total (95% CI)**			**196**			**195**	**100.0%**	**-0.44 [-0.65, -0.23]**	
Heterogeneity: Tau² = 0.04; Chi² = 17.31, df = 5 (P = 0.004); I² = 71%									
Test for overall effect: Z = 4.11 (P < 0.0001)									
Test for subgroup differences: Chi² = 0.34, df = 1 (P = 0.56), I² = 0%									

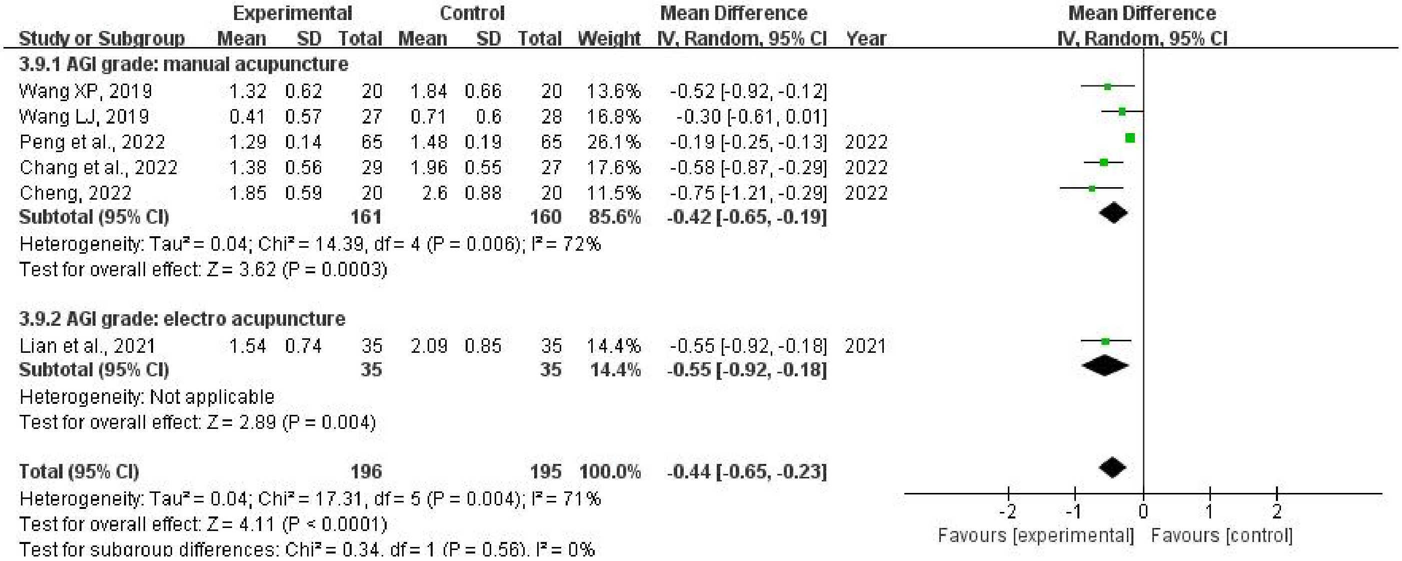

6.10 Subgroup analysis of APACHE-II according to the duration of acupuncture

Study or Subgroup	Experimental Mean	Experimental SD	Experimental Total	Control Mean	Control SD	Control Total	Weight	Mean Difference IV, Random, 95% CI	Year
**3.10.1 APACHE-II: acupuncture duration =5 days**									
Wang LJ, 2019	11.19	4.28	27	11.36	3.74	28	9.9%	-0.17 [-2.30, 1.96]	
**Subtotal (95% CI)**			**27**			**28**	**9.9%**	**-0.17 [-2.30, 1.96]**	
Heterogeneity: Not applicable									
Test for overall effect: Z = 0.16 (P = 0.88)									
**3.10.2 APACHE-II: acupuncture duration =7 days**									
Li et al., 2021	14.33	1.26	28	15.6	1.22	29	15.8%	-1.27 [-1.91, -0.63]	
Yang, 2018	15.13	5.73	30	18.8	5.95	30	7.1%	-3.67 [-6.63, -0.71]	
Wang XP, 2019	19.88	3.83	20	22.43	4.4	20	8.4%	-2.55 [-5.11, 0.01]	
Lian et al., 2021	14.29	3.66	35	15.34	4.81	35	10.4%	-1.05 [-3.05, 0.95]	2021
Cheng, 2022	11.96	5.84	31	15.56	7.27	32	6.4%	-3.60 [-6.85, -0.35]	2022
Ban et al., 2022	14.62	1.46	37	16.36	2.69	36	14.5%	-1.74 [-2.74, -0.74]	2022
**Subtotal (95% CI)**			**181**			**182**	**62.5%**	**-1.57 [-2.10, -1.04]**	
Heterogeneity: Tau² = 0.02; Chi² = 5.20, df = 5 (P = 0.39); I² = 4%									
Test for overall effect: Z = 5.79 (P < 0.00001)									
**3.10.3 APACHE-II: acupuncture duration =14 days**									
Sun et al., 2022	16.81	2.08	27	17.54	4.55	26	10.7%	-0.73 [-2.65, 1.19]	
Peng et al., 2022	11.06	2.05	65	14.96	1.97	65	15.6%	-3.90 [-4.59, -3.21]	2022
**Subtotal (95% CI)**			**92**			**91**	**26.3%**	**-2.45 [-5.54, 0.65]**	
Heterogeneity: Tau² = 4.48; Chi² = 9.30, df = 1 (P = 0.002); I² = 89%									
Test for overall effect: Z = 1.55 (P = 0.12)									
**3.10.4 APACHE-II: acupuncture duration =28 days**									
Wei, 2017	19.1	15.3	20	20.8	13.4	21	1.3%	-1.70 [-10.52, 7.12]	
**Subtotal (95% CI)**			**20**			**21**	**1.3%**	**-1.70 [-10.52, 7.12]**	
Heterogeneity: Not applicable									
Test for overall effect: Z = 0.38 (P = 0.71)									
**Total (95% CI)**			**320**			**322**	**100.0%**	**-1.99 [-3.04, -0.95]**	
Heterogeneity: Tau² = 1.69; Chi² = 40.83, df = 9 (P < 0.00001); I² = 78%									
Test for overall effect: Z = 3.74 (P = 0.0002)									
Test for subgroup differences: Chi² = 1.93, df = 3 (P = 0.59), I² = 0%									

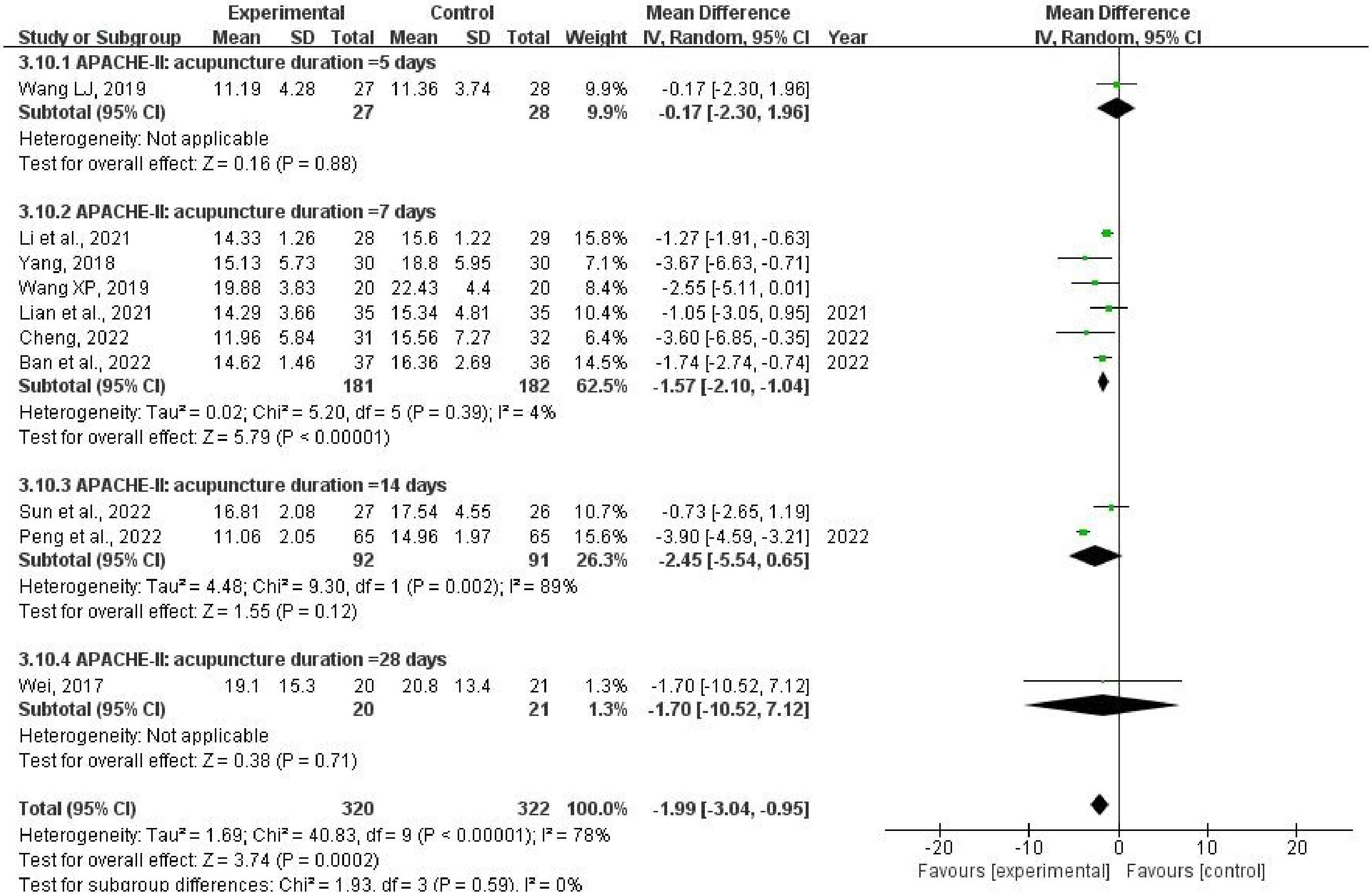

6.11 Subgroup analysis of APACHE-II according to different methods of acupuncture

Study or Subgroup	Experimental Mean	Experimental SD	Experimental Total	Control Mean	Control SD	Control Total	Weight	Mean Difference IV, Random, 95% CI
**3.11.1 APACHE-II: manual acupuncture**								
Wei, 2017	19.1	15.3	20	20.8	13.4	21	1.6%	-1.70 [-10.52, 7.12]
Yang, 2018	15.13	5.73	30	18.8	5.95	30	8.6%	-3.67 [-6.63, -0.71]
Wang XP, 2019	19.88	3.83	20	22.43	4.4	20	10.0%	-2.55 [-5.11, 0.01]
Wang LJ, 2019	11.19	4.28	27	11.36	3.74	28	11.8%	-0.17 [-2.30, 1.96]
Ban et al., 2022	14.62	1.46	37	16.36	2.69	36	17.0%	-1.74 [-2.74, -0.74]
Peng et al., 2022	11.06	2.05	65	14.96	1.97	65	18.2%	-3.90 [-4.59, -3.21]
Cheng, 2022	11.96	5.84	31	15.56	7.27	32	7.7%	-3.60 [-6.85, -0.35]
**Subtotal (95% CI)**			**230**			**232**	**74.9%**	**-2.54 [-3.84, -1.24]**
Heterogeneity: Tau² = 1.65; Chi² = 19.98, df = 6 (P = 0.003); I² = 70%								
Test for overall effect: Z = 3.83 (P = 0.0001)								
**3.11.2 APACHE-II: electro acupuncture**								
Lian et al., 2021	14.29	3.66	35	15.34	4.81	35	12.3%	-1.05 [-3.05, 0.95]
Sun et al., 2022	16.81	2.08	27	17.54	4.55	26	12.7%	-0.73 [-2.65, 1.19]
**Subtotal (95% CI)**			**62**			**61**	**25.1%**	**-0.88 [-2.27, 0.50]**
Heterogeneity: Tau² = 0.00; Chi² = 0.05, df = 1 (P = 0.82); I² = 0%								
Test for overall effect: Z = 1.25 (P = 0.21)								
**Total (95% CI)**			**292**			**293**	**100.0%**	**-2.12 [-3.29, -0.96]**
Heterogeneity: Tau² = 1.80; Chi² = 27.98, df = 8 (P = 0.0005); I² = 71%								
Test for overall effect: Z = 3.59 (P = 0.0003)								
Test for subgroup differences: Chi² = 2.93, df = 1 (P = 0.09), I² = 65.8%								

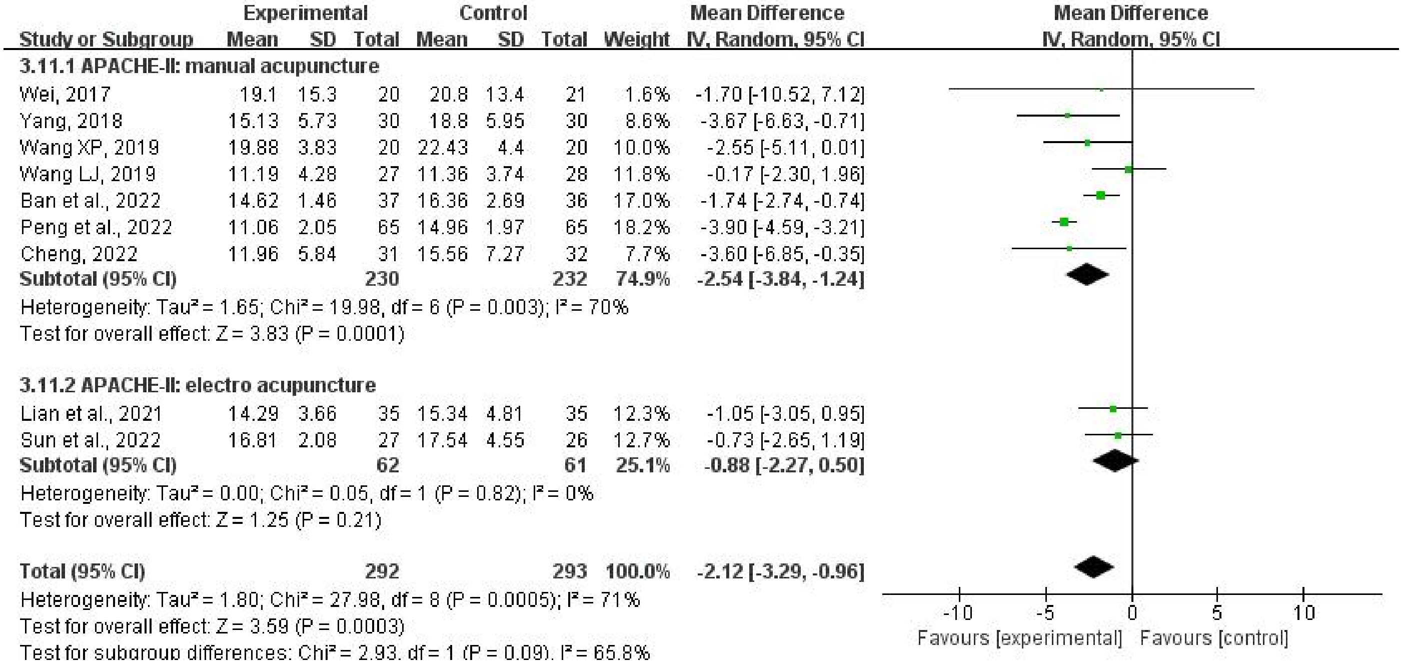

6.12 Subgroup analysis of frequency of borborygmus according to the baseline of APACHE II

Study or Subgroup	Experimental Mean	Experimental SD	Experimental Total	Control Mean	Control SD	Control Total	Weight	Mean Difference IV, Random, 95% CI
**3.12.1 Frequency of borborygmus per minute: APACHE II ⩾22**								
Wei, 2017	4.5	1.2	20	3.6	1.9	21	8.6%	0.90 [-0.07, 1.87]
Yang, 2018	3.87	1.22	30	3.07	1.17	30	14.1%	0.80 [0.20, 1.40]
Wang XP, 2019	3.47	0.75	20	2.76	0.69	20	17.4%	0.71 [0.26, 1.16]
Wang LJ, 2019	2.22	1.93	27	0.54	1.75	28	8.5%	1.68 [0.71, 2.65]
Liu et al., 2020	2.22	1.93	27	0.54	1.75	28	8.5%	1.68 [0.71, 2.65]
Peng et al., 2022	3.96	0.98	65	3.48	0.53	65	21.1%	0.48 [0.21, 0.75]
**Subtotal (95% CI)**			**189**			**192**	**78.2%**	**0.87 [0.51, 1.22]**
Heterogeneity: $Tau^2 = 0.09$; $Chi^2 = 10.55$, df = 5 (P = 0.06); $I^2 = 53\%$								
Test for overall effect: Z = 4.74 (P < 0.00001)								
**3.12.2 Frequency of borborygmus per minute: APACHE II <22**								
Wang LJ, 2019	2.22	1.93	27	0.54	1.75	28	8.5%	1.68 [0.71, 2.65]
**Subtotal (95% CI)**			**27**			**28**	**8.5%**	**1.68 [0.71, 2.65]**
Heterogeneity: Not applicable								
Test for overall effect: Z = 3.38 (P = 0.0007)								
**3.12.3 Frequency of borborygmus per minute: APACHE-II not reported**								
Liu et al., 2020	3.47	1.81	49	2.15	1.46	49	13.3%	1.32 [0.67, 1.97]
**Subtotal (95% CI)**			**49**			**49**	**13.3%**	**1.32 [0.67, 1.97]**
Heterogeneity: Not applicable								
Test for overall effect: Z = 3.97 (P < 0.0001)								
**Total (95% CI)**			**265**			**269**	**100.0%**	**1.02 [0.67, 1.37]**
Heterogeneity: $Tau^2 = 0.14$; $Chi^2 = 17.33$, df = 7 (P = 0.02); $I^2 = 60\%$								
Test for overall effect: Z = 5.64 (P < 0.00001)								
Test for subgroup differences: $Chi^2 = 3.27$, df = 2 (P = 0.20), $I^2 = 38.8\%$								

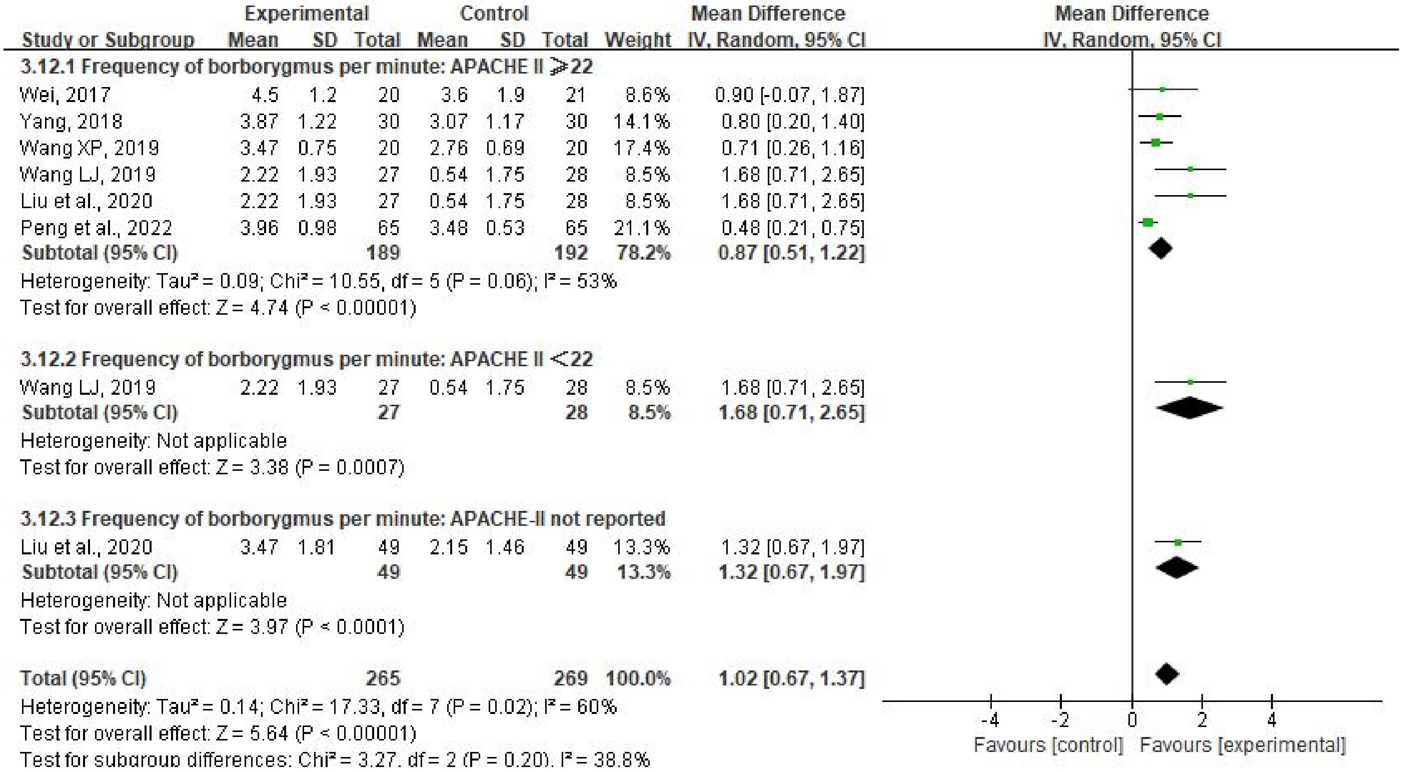

6.13 Subgroup analysis of frequency of borborygmus according to the duration of acupuncture

Study or Subgroup	Control Mean	Control SD	Control Total	Experimental Mean	Experimental SD	Experimental Total	Weight	Mean Difference IV, Random, 95% CI	Year
**3.13.1 Frequency of borborygmus per minute: acupuncture duration = 5 days**									
Wang LJ, 2019	2.22	1.93	27	0.54	1.75	28	8.7%	1.68 [0.71, 2.65]	
**Subtotal (95% CI)**			**27**			**28**	**8.7%**	**1.68 [0.71, 2.65]**	
Heterogeneity: Not applicable									
Test for overall effect: Z = 3.38 (P = 0.0007)									
**3.13.2 Frequency of borborygmus per minute: acupuncture duration = 7 days**									
Yang, 2018	3.87	1.22	30	3.07	1.17	30	16.4%	0.80 [0.20, 1.40]	
Wang XP, 2019	3.47	0.75	20	2.76	0.69	20	21.8%	0.71 [0.26, 1.16]	
Liu et al., 2020	3.47	1.81	49	2.15	1.46	49	15.1%	1.32 [0.67, 1.97]	
**Subtotal (95% CI)**			**99**			**99**	**53.3%**	**0.89 [0.54, 1.24]**	
Heterogeneity: $Tau^2 = 0.02$; $Chi^2 = 2.38$, df = 2 (P = 0.30); $I^2 = 16\%$									
Test for overall effect: Z = 5.01 (P < 0.00001)									
**3.13.4 Frequency of borborygmus per minute: acupuncture duration = 14 days**									
Peng et al., 2022	3.96	0.98	65	3.48	0.53	65	29.1%	0.48 [0.21, 0.75]	2022
**Subtotal (95% CI)**			**65**			**65**	**29.1%**	**0.48 [0.21, 0.75]**	
Heterogeneity: Not applicable									
Test for overall effect: Z = 3.47 (P = 0.0005)									
**3.13.5 Frequency of borborygmus per minute: acupuncture duration = 28 days**									
Wei, 2017	4.5	1.2	20	3.6	1.9	21	8.8%	0.90 [-0.07, 1.87]	
**Subtotal (95% CI)**			**20**			**21**	**8.8%**	**0.90 [-0.07, 1.87]**	
Heterogeneity: Not applicable									
Test for overall effect: Z = 1.82 (P = 0.07)									
**Total (95% CI)**			**211**			**213**	**100.0%**	**0.85 [0.52, 1.18]**	
Heterogeneity: $Tau^2 = 0.08$; $Chi^2 = 10.17$, df = 5 (P = 0.07); $I^2 = 51\%$									
Test for overall effect: Z = 5.04 (P < 0.00001)									
Test for subgroup differences: $Chi^2 = 7.70$, df = 3 (P = 0.05), $I^2 = 61.0\%$									

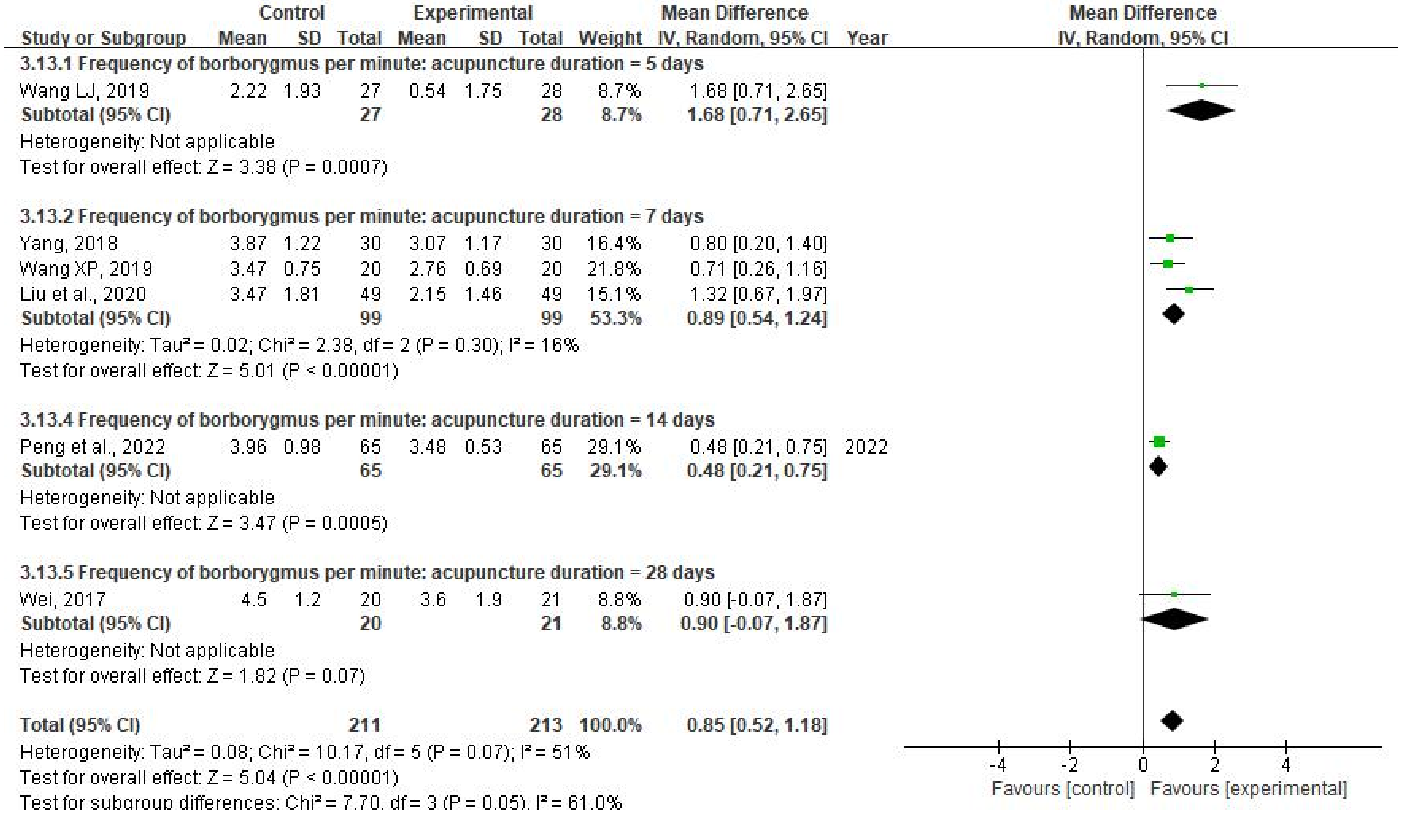

Supplementary File S7. Quality of evidence

Outcomes	Anticipated absolute effects* (95% CI)		Relative effect (95% CI)	№ of participants (studies)	Certainty of the evidence (GRADE)	Comments
	**Risk with control group**	**Risk with intervention group**				
Intra-abdominal pressure (IAP)	-	SMD **0.71 lower** (1.01 lower to 0.41 lower)	-	759 (11 RCTs)	⊕◯◯◯ Very low[a,b]	Risk of bias (-1)[a]; Inconsistency (-2)[b]
Acute gastrointestinal injury		MD **0.44 lower** (0.65 lower to 0.23 lower)	-	391 (6 RCTs)	⊕⊕⊕◯ Moderate[a]	Risk of bias (-1)[a]
APACHE- II		MD **1.99 lower** (3.04 lower to 0.95 lower)	-	642 (10 RCTs)	⊕◯◯◯ Very low[a,b]	Risk of bias (-1)[a];Inconsistency (-2)[b]
Frequency of borborygmus		MD **0.85 higher** (0.52 higher to 1.18 higher)	-	424 (6 RCTs)	⊕⊕◯◯ Low[a,c]	Risk of bias (-1)[a];Inconsistency (-1)[c]
Abdominal premeter		MD **2.24 lower** (3.49 lower to 1 lower)	-	401 (6 RCTs)	⊕⊕⊕◯ Moderate[a]	Risk of bias (-1)[a]
Mortality at day 28	188 per 1,000	**122 per 1,000** (70 to 216)	**RR 0.65** (0.37 to 1.15)	264 (5 RCTs)	⊕⊕⊕◯ Moderate[a]	Risk of bias (-1)[a]

***The risk in the intervention group** (and its 95% confidence interval) is based on the assumed risk in the comparison group and the **relative effect** of the intervention (and its 95% CI).

CI: confidence interval; **MD:** mean difference; **SMD:** standardised mean difference; **RR:** risk ratio

GRADE Working Group grades of evidence
High certainty: we are very confident that the true effect lies close to that of the estimate of the effect.
Moderate certainty: we are moderately confident in the effect estimate: the true effect is likely to be close to the estimate of the effect, but there is a possibility that it is substantially different.
Low certainty: our confidence in the effect estimate is limited: the true effect may be substantially different from the estimate of the effect.
Very low certainty: we have very little confidence in the effect estimate: the true effect is likely to be substantially different from the estimate of effect.

Explanations

a. Majority of the data is derived from studies with low risk or some concerns, and there are potential limitations that may reduce certainty in determining the effect.

b. The direction of the effect is different and $I^2 \geq 75\%$.

c. The direction of the effect is different and $50 < I^2 < 75\%$
